# Age-specific trends in colorectal, appendiceal, and anal tumour incidence by histological subtype in Australia from 1990 to 2020: a population-based time-series analysis

**DOI:** 10.1016/j.lanwpc.2025.101728

**Published:** 2025-10-30

**Authors:** Aaron L. Meyers, James G. Dowty, Khalid Mahmood, Finlay A. Macrae, Christophe Rosty, Daniel D. Buchanan, Mark A. Jenkins

**Affiliations:** a Centre for Epidemiology and Biostatistics, Melbourne School of Population and Global Health, University of Melbourne, Parkville, Victoria, Australia; b Collaborative Centre for Genomic Cancer Medicine, Victorian Comprehensive Cancer Centre, Melbourne, Victoria, Australia; c Colorectal Oncogenomics Group, Department of Clinical Pathology, Melbourne Medical School, University of Melbourne, Parkville, Victoria, Australia; d Melbourne Bioinformatics, University of Melbourne, Parkville, Victoria, Australia; e Colorectal Medicine and Genetics, Royal Melbourne Hospital, Parkville, Victoria, Australia; f Genomic Medicine and Family Cancer Clinic, Royal Melbourne Hospital, Parkville, Victoria, Australia; g Department of Medicine, University of Melbourne, Parkville, Victoria, Australia; h Envoi Specialist Pathologists, Brisbane, Queensland, Australia; i University of Queensland, Brisbane, Queensland, Australia

**Keywords:** Colorectal cancer, Appendiceal cancer, Anal cancer, Histological subtype, Early-onset, Incidence

## Abstract

**Background:**

Early-onset bowel cancer incidence (age <50 years) has increased worldwide and is highest in Australia, but how trends vary across age, histology, and anatomical site remains unclear. We investigated appendiceal, proximal colon, distal colon, rectal, and anal cancer incidence trends by age and histology in Australia.

**Methods:**

Cancer incidence rate data were obtained from nation-wide cancer registries (1990–2020). Birth cohort-specific incidence rate ratios (IRRs) and annual percentage change were estimated using age-period-cohort modelling and joinpoint regression.

**Findings:**

Combining all malignant tumour histologies, early-onset incidence rose 5–9% annually, yielding 4072 excess cases (1·5 per 100,000 person-years; 15% appendix, 28% colon, 48% rectum, 9% anus). Trends varied by site, period, and age: appendiceal cancer rose from 1990 to 2020 in 30–49-year-olds; colorectal cancers rose from around 1990–2010 in 20–29-year-olds and from 2010 to 2020 in 30–39-year-olds; anal cancer rose from 1990 to 2009 in 40–49-year-olds. Across sites, IRRs increased with successive birth cohorts since 1960. Adenocarcinoma incidence in the 1990s versus 1950s cohort was 2–3-fold for colorectum and 7-fold for appendix. The greatest subtype-specific increases occurred for appendiceal mucinous adenocarcinoma, colorectal non-mucinous adenocarcinoma, and anal squamous cell carcinoma. Colorectal neuroendocrine neoplasms, squamous cell carcinomas, and signet ring cell carcinomas rose across early-onset and later-onset strata.

**Interpretation:**

Appendiceal, colorectal, and anal cancer incidence is rising in Australia, with variation across age and histology. These generational shifts suggest evolving risk factor profiles and early-life exposures, highlighting the need to identify drivers of this growing burden.

**Funding:**

Australian Government.

**Research in context**

**Evidence before this study**

We searched PubMed for studies in English on bowel cancer trends published until April 10th, 2025, using the terms (“colorectal cancer” OR “colon cancer” OR “rectal cancer” OR “bowel cancer”) AND (“incidence”) AND (“trend”) AND (“age” OR “early-onset” OR “young-onset”). Multiple studies have reported rising early-onset (age <50 years) anal and colorectal (including appendix) cancer incidence in high-income countries, with rates highest in Australia. In many countries, the rise is exclusive to distal colorectal cancers, and a portion of the increase in the USA has been ascribed to appendiceal tumour and neuroendocrine neoplasm reclassification. Compared to later-onset cases, early-onset colorectal cancers are more likely to occur distally and harbour mucinous or signet ring cell histology – subtypes which exhibit aetiological heterogeneity. However, few time-series studies have analysed incidence trends according to anatomical subsite within the colon; separated the appendix from colon; and/or stratified tumours by histological subtype.

**Added value of this study**

This study is the first to systematically examine trends of appendiceal, proximal colon, distal colon, rectal, and anal cancer incidence by histological subtype in Australia across age, period, and birth cohort. After excluding neuroendocrine neoplasms, early-onset cancer incidence rose across all sites since 1990; for the first time, we ascribe 4072 excess cases (1·5 per 100,000 person-years) to this increase. Rising incidence of early-onset colorectal cancer, alongside reductions in older adults, is a well-established pattern that we reveal is specific to conventional adenocarcinoma, likely reflecting the opposing influence of risk factors and population screening. We find similar patterns and magnitudes of birth cohort trends for distal colon, proximal colon, and rectal cancers, suggesting increasing prevalence of risk factors in Australia that indiscriminately promote colorectal carcinogenesis. Incidence of colonic signet ring cell carcinoma increased across early-onset and later-onset strata, while colorectal mucinous adenocarcinoma decreased across all age groups, despite the widely-recognised enrichment of these subtypes within early-onset cases. A portion of the increase in rectal cancer was attributable to human papillomavirus-related squamous cell carcinoma. Increases were most pronounced for appendiceal cancer (irrespective of histology) and neuroendocrine neoplasms (irrespective of site), although the role of shifting risk factor profiles versus diagnostic scrutiny remains to be determined.

**Implications of all the available evidence**

The generational shift in gastrointestinal cancer risk reflects earlier risk factor exposure among generations born after 1960. This foreshadows future disease burden as young cohorts carry elevated risk into older age, and may tip the benefit-to-harm ratio of screening younger populations. Early-onset tumour subtypes increasing in incidence are different from those enriched in case-series, suggesting that factors driving the increase are not the same as those underlying early-onset colorectal cancer itself. To uncover causes for these concerning temporal trends, lifecourse studies are needed to identify cohort-specific exposures and trajectories of susceptibility to tumour subtypes that are increasing in incidence. Meanwhile, healthcare providers and policymakers must emphasise prevention and early detection by improving awareness about known risk factors, signs, and symptoms.

## Introduction

Colorectal cancer is the third most common cancer and second leading cause of cancer-related deaths worldwide, accounting for almost 2 million new cases and 1 million deaths in 2022.^1^ Incidence of early-onset colorectal cancer (diagnosed before age 50 years) is increasing worldwide, with rates highest in Australia.^2^ This contrasts stabilising or declining incidence among Australians above age 50, a phenomenon attributed to population-based screening available to people aged 50–74.^2^ The cause of increasing early-onset incidence is unknown, although increasing risk factor exposure among younger generations is hypothesised.^2^^,^^3^ This is supported by reports of a birth cohort effect, with colorectal cancer incidence 3–4-fold higher among Australians born in the 1980s versus 1950s.^3^ Nevertheless, establishing drivers of the growing disease burden remains elusive, complicated by the aetiological variability of colorectal cancer.

Colorectal cancers encompass a range of subtypes with extensive risk factor heterogeneity. Diabetes more strongly associates with proximal (right-sided) than distal (left-sided) colon cancer.^4^ Conversely, physical activity and obesity more strongly associate with colon cancer, while aspirin and smoking more strongly associate with rectal cancer.^5^ These patterns could be due to risk factor associations varying by clinicopathological features. For example, proximal colon cancers are more likely to develop from serrated polyps and harbour mucinous or signet ring cell differentiation.^6^ Tumour pathology can also reflect aetiology that varies by age, with early-onset colorectal cancers preferentially localising to the distal colorectum and displaying aggressive histology, including poor differentiation and mucinous or signet ring morphology, compared with later-onset cases.^7^ Such differences suggest that risk factors may differ for early-onset disease.

Few studies have examined which subtypes drive the rising incidence, with inconsistent findings. In Europe and Australia, the rise appears more pronounced for colon than rectal cancer, while the opposite is reported for North America.^2^ This could reflect inconsistent classification, with appendix defined as colon cancer by European and Australian but not North American registries.^2^^,^^3^ An alternative explanation is an artefactual increase from reclassifying low-grade neuroendocrine neoplasms as malignant in early 2000s, which could upwardly bias the rectal-to-colon cancer ratio.^8^ This underscores the importance of analysing trends by anatomical and histological subtype.

Anal cancer incidence has also increased among young adults aged 40–49 years,^9^ suggesting that causes for rising early-onset cancer may affect risk across the lower gastrointestinal tract. However, no study has explored temporal and age-specific dynamics of appendiceal, colorectal, and anal cancer histotypes in Australia. Such an analysis is crucial to highlight shared or distinct risk factors driving recent trends, identify subtypes warranting further aetiological investigation, and inform discourse around lowering the age for population screening, which targets colorectal adenocarcinoma. To address these gaps, we present for the first time an analysis of variations in appendiceal, proximal colon, distal colon, rectal, and anal cancer incidence by histological subtype in Australia across age, period, and birth cohort.

## Methods

### Study design and data sources

This nationwide time-series included data on all incident appendiceal, colorectal, and anal cancers diagnosed in Australia from 1990 to 2020. Cases were defined by anatomical site of the primary diagnosis. Aggregated cancer records for each histology, topography, sex, diagnosis year, and 10-year age group (20–29 to 90+ years) were obtained from the Australian Institute of Health and Welfare, which collates population-based data from all state and territory cancer registries. Cancer is a notifiable condition and Australian cancer registry data is reported as high quality.^3^ Corresponding population figures were obtained from the Australian Bureau of Statistics. Ethical approval was not required as no patient-level data was accessed or used.

### Tumour subtyping

The following malignant subsite categories were included: appendix (International Classification of Diseases for Oncology, 3rd revision: C18.1), proximal colon (cecum, ascending colon, hepatic flexure, transverse colon, splenic flexure; C18.0, C18.2–C18.5), distal colon (descending and sigmoid colon; C18.6, C18.7), overlapping/unspecified colon (C18.8, C18.9), rectum (C19.9, C20.9), anus (C21). Tumours were further classified into non-overlapping histological subtypes: adenocarcinoma not otherwise specified (NOS); adenocarcinoma in a polyp; mucinous adenocarcinoma; signet ring cell carcinoma; squamous cell carcinoma; carcinoma NOS; neuroendocrine neoplasm; melanoma; sarcoma; other (all remaining codes, including unknown) (Appendix Table S1).

### Statistical analysis

The period-specific, sex-specific, and age-specific (20–49, ≥50 years) percentage of all histotypes was calculated using the sum of all new cases within each tumour site as the denominator. To adjust for age differences, 10-year age-specific histotype proportions were combined using direct age-standardisation to the corresponding sex-specific and tumour site-specific Australian case population from the median (2005) year. We selected age strata to define early-onset (age 20–49 years) and later-onset (age ≥50 years) cases based on previous studies to capture changing histotype distributions across age.^7^

Annual age-specific and site-specific incidence rates (IRs) per 100,000 person-years were estimated overall and also estimated after stratifying by sex and histology. To quantify overall disease burden, our primary analysis considered all histological subtypes combined, excluding neuroendocrine neoplasms to avoid confounding from reclassification of indolent subtypes to malignant that occurred across early 2000s.^8^ Secondary analyses of all histological subtypes combined were undertaken with neuroendocrine neoplasms included to assess the impact of the reclassification on results.^8^

To evaluate temporal trends that may have occurred across both historical and recent time periods, joinpoint regression models were fitted to identify inflection years for changes in the direction and magnitude of IR trends, which may reflect changes in clinical or public health practice.^10^ The optimal joinpoint number was based a weighted Bayesian information criterion (WBIC), which combines classical BIC and BIC_3_ using the partial $R^{2}$ to determine the weight between BIC and BIC_3_ as:

$$WBIC=\log\left(\frac{{RSS}_{k}}{n}\right)+\left(2+R_{\max}^{2}\left(k\right)\right)\frac{k\;\log\;n}{n}$$

where ${RSS}_{k}$ denotes the residual sum of squares for the model fit with k joinpoints, n is the number of observations, and $R_{\max}^{2}\left(k\right)=\max_{i=1,\ldots ,k}R_{i,i+1}^{2}$.^10^ The magnitude of rate changes was expressed as annual percentage change (APC), quantified using the gradient of each segmented period. Average APC (AAPC) was based on a weighted geometric mean across segments.^10^ Non-parametric confidence intervals (CIs) were computed using the empirical quantile method with 5000 resamples.^10^ This approach computes resampled residuals by applying the inverse of the empirical distribution function of the original residuals to uniform random values over (0,1), and adds resampled residuals to the original fit. The model is fit to each resampled dataset, and 95% CIs are defined at the 2·5th and 97·5th percentiles of the resampled APC estimates. To obtain stable estimates, we truncated periods with 0 cases and limited subgroup analyses to common histotypes (>3%) (Appendix pp 10–14, 25–30).

Age-period-cohort models were fitted to evaluate birth cohort trends, which help identify generational shifts in susceptibility due to elevated or earlier risk factor exposure.^11^ We assumed Poisson-distributed counts and overdispersion parameters for excess-Poisson variation.^11^ Ten-year intervals were used to define eight age groups (20–29 to 90–99), three diagnosis periods (1990–1999, 2000–2009, 2010–2019), and 10 birth cohorts for each decade from 1900 to 1999. We computed incidence rate ratios (IRRs) comparing net IR in each cohort to the 1950 (middle) cohort. To examine period effects across subtypes, including rarer groups that could not be reliably modelled using joinpoint regression due to small numbers, we estimated AAPC in rates from 1990 to 2019 using model-based local drifts; i.e., gradients of IRR curves within each age stratum. This approach provides a comprehensive estimate of temporal trends by accounting for both period and cohort effects within each age group. Wald tests were used to assess non-linear components and identify heterogeneity of local drifts compared to age-standardised AAPC (net drift).

Excess early-onset cases attributable to rising incidence were estimated per annum as the difference between observed and expected numbers had there been no rate increases. Given the overall increasing IR during the study period, annual excess cases were estimated relative to a counterfactual AAPC of 0%. This was done by multiplying the population size by the difference between the model-based IR (reflecting the estimated AAPC) and the intercept from log-linear joinpoint models (i.e., model-based estimates of the baseline IR in 1990). Colon subsites were combined due to comparable rates and trends. Expected cases were presented as the difference between recorded and excess cases. To avoid implausible estimates, excess cases were capped at the recorded number. P-scores were defined as the ratio of excess to expected cases, expressed as a percentage, where negative values correspond to fewer cases than expected and positive values correspond to increasing relative excess cases.^12^ Absolute excess risk (AER) was defined as the number of excess cases per 100,000 person-years. Uncertainty intervals (UI) were derived from the CI of the AAPC. Hypothesis tests were two-sided and tested at alpha = 0·05. Statistical analyses were undertaken using Joinpoint Regression Program (version 5.2) and R (version 4.2.2).

### Role of the funding source

The funders of the study had no role in study design, data collection, data analysis, data interpretation, or writing of the report.

## Results

**Fig. 1:**
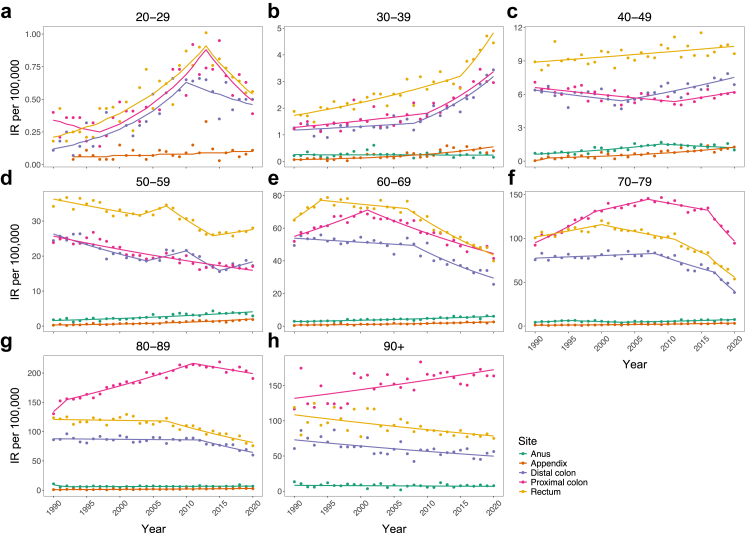
Period trends in cancer incidence by anatomical site and age from 1990 to 2020. Joinpoint regression was used to fit a series of joined straight lines to cancer rates per 100,000 Australians aged **a**: 20–29; **b**: 30–39; **c**: 40–49; **d**: 50–59; **e**: 60–69; **f**: 70–79; **g**: 80–89; and **h**: 90+ years. The optimal number of segments was based on the weighted Bayesian information criterion method. Annual percentage changes and 95% confidence intervals for each segment are given in Table 1. Data exclude neuroendocrine neoplasms. Results for all histological subtypes combined and by sex and histology are presented in the Appendix pp 3–7 and 10–14. IR, incidence rate. Note: the scale of Y-axes differ.

Between 1990 and 2020, a total of 413,691 appendiceal, colorectal, and anal tumours were diagnosed among Australians aged ≥20 years (appendix, 1·9%; proximal colon, 35·2%; distal colon, 22·5%; overlapping/unspecified colon, 5·3%; rectum, 32·6%; anus, 2·4%) (Table 1, Appendix Table S2). Of these, 34,907 (8·4%) occurred before age 50 (early-onset), which were evenly distributed between sexes (50·1% men). Most early-onset tumours were localised to the rectum (37·6%), followed by proximal colon (22·4%), distal colon (22·2%), appendix (10·6%), and anus (3·9%). After excluding neuroendocrine neoplasms, IRs were highest for rectal cancer among 20–69-year-olds, whereas proximal colon cancer rates were highest in those aged ≥70 (Fig. 1). Trends were consistent between men and women (Appendix Fig. S2–S5).

**Table 1:** Temporal trends in tumour incidence rates by age, anatomical site, and calendar period of diagnosis.

Age, years	*n*	Trend 1		Trend 2		Trend 3		Trend 4	
		Period	APC (95% CI)	Period	APC (95% CI)	Period	APC (95% CI)	Period	APC (95% CI)
**Appendix**									
20–29	81	1993–2020	2·2 (−0·4, 4·8)	–	–	–	–	–	–
30–39	244	1990–2020	7·4 (5·1, 9·7)	–	–	–	–	–	–
40–49	611	1990–1992	123·7 (19·3, 270·4)	1992–2020	5·4 (2·9, 7·0)	–	–	–	–
50–59	798	1990–2020	6·3 (5·1, 7·5)	–	–	–	–	–	–
60–69	910	1990–2020	4·7 (3·8, 5·6)	–	–	–	–	–	–
70–79	788	1990–2020	4·5 (3·5, 5·5)	–	–	–	–	–	–
80–89	368	1990–2020	3·9 (2·0, 5·9)	–	–	–	–	–	–
90+	50	–	–	–	–	–	–	–	–
**Proximal colon**									
20–29	482	1990–1997	−4·6 (−24·7, 3·3)	1997–2013	8·3 (5·8, 28·9)	2013–2020	−8·0 (−21·6, 0·0)	–	–
30–39	1849	1990–2010	1·8 (−6·6, 3·3)	2010–2020	6·4 (2·8, 24·6)	–	–	–	–
40–49	5314	1990–2011	−1·0 (−3·4, −0·5)	2011–2020	1·7 (−0·2, 10·0)	–	–	–	–
50–59	14,983	1990–2020	−1·6 (−1·9, −1·3)	–	–	–	–	–	–
60–69	32,936	1990–2001	2·4 (1·5, 3·5)	2001–2020	−2·4 (−2·9, −2·1)	–	–	–	–
70–79	48,249	1990–1999	3·6 (2·7, 6·2)	1999–2007	1·3 (−0·3, 2·9)	2007–2016	−1·0 (−2·7, 0·1)	2016–2020	−7·5 (−10·6, −5·6)
80–89	35,015	1990–1992	7·3 (1·9, 12·1)	1992–2011	1·8 (−3·0, 2·1)	2011–2020	−0·9 (−2·6, 1·4)	–	–
90+	5311	1990–2020	0·9 (0·4, 1·4)	–	–	–	–	–	–
**Distal colon**									
20–29	384	1990–2010	8·6 (6·1, 20·5)	2010–2020	−3·1 (−25·3, 3·7)	–	–	–	–
30–39	1657	1990–2008	1·1 (−2·1, 2·4)	2008–2020	6·9 (4·4, 15·0)	–	–	–	–
40–49	5633	1990–2003	−1·3 (−4·7, 0·0)	2003–2020	2·0 (1·1, 4·2)	–	–	–	–
50–59	15,019	1990–2004	−2·4 (−3·4, −1·8)	2004–2010	2·4 (0·2, 7·6)	2010–2015	−5·8 (−10·8, −3·1)	2015–2020	2·9 (0·2, 9·3)
60–69	25,644	1990–2008	−0·5 (−1·0, 0·2)	2008–2020	−4·2 (−5·6, −3·3)	–	–	–	–
70–79	27,593	1990–2008	0·4 (0·0, 0·9)	2008–2017	−3·3 (−4·7, −1·8)	2017–2020	−13·4 (−19·6, −9·2)	–	–
80–89	14,858	1990–2012	−0·1 (−0·5, 0·5)	2012–2020	−3·5 (−7·1, −1·9)	–	–	–	–
90+	1971	1990–2020	−1·3 (−1·8, −0·7)	–	–	–	–	–	–
**Rectum**									
20–29	519	1990–2013	6·7 (5·2, 9·4)	2013–2020	−7·2 (−25·6, 0·7)	–	–	–	–
30–39	2618	1990–2015	2·5 (−0·1, 3·2)	2015–2020	8·6 (3·1, 23·8)	–	–	–	–
40–49	8686	1990–2020	0·5 (0·2, 0·8)	–	–	–	–	–	–
50–59	23,261	1990–2003	−1·0 (−2·6, −0·6)	2003–2007	2·0 (−0·4, 4·5)	2007–2014	−4·0 (−7·3, −2·7)	2014–2020	1·1 (−0·4, 4·3)
60–69	36,621	1990–1994	4·1 (1·0, 10·2)	1994–2007	−0·5 (−1·7, 0·1)	2007–2020	−3·8 (−4·6, −3·2)	–	–
70–79	36,670	1990–2000	1·3 (−2·2, 5·3)	2000–2011	−1·4 (−4·5, 4·8)	2011–2016	−4·0 (−8·8, 1·0)	2016–2020	−8·8 (−13·3, −5·3)
80–89	19,414	1990–2007	−0·1 (−0·7, 0·6)	2007–2020	−2·8 (−4·0, −2·1)	–	–	–	–
90+	3020	1990–2020	−1·1 (−1·6, −0·6)	–	–	–	–	–	–
**Anus**									
20–29	36	–	–	–	–	–	–	–	–
30–39	251	1990–2020	−0·2 (−1·8, 1·4)	–	–	–	–	–	–
40–49	1055	1990–2009	4·8 (3·5, 7·2)	2009–2020	−2·2 (−7·4, 0·6)	–	–	–	–
50–59	2153	1990–2020	3·2 (2·3, 4·0)	–	–	–	–	–	–
60–69	2574	1990–2020	2·5 (2·1, 3·0)	–	–	–	–	–	–
70–79	2186	1990–1995	7·6 (0·3, 22·6)	1995–2003	−4·6 (−15·3, −0·4)	2003–2020	3·2 (1·7, 5·7)	–	–
80–89	1198	1990–1992	−19·9 (−30·0, 0·0)	1992–2020	0·6 (−0·2, 3·1)	–	–	–	–
90+	267	1990–2020	−0·6 (−2·5, 1·3)	–	–	–	–	–	–

Note: Excludes neuroendocrine neoplasms. Estimates for all histotypes combined and by sex and histology are provided in Appendix Tables S3–S10.

APC, annual percentage change; CI, confidence interval; *n*, number of cases.

Since 1990, multiple periods of increasing early-onset proximal and distal colon cancer were detected after excluding neuroendocrine neoplasms in the primary analysis, with increases most pronounced at younger ages (Fig. 1, Table 1). For ages 20–29 years, increases occurred from 1997 to 2013 for proximal colon (APC 8·3% [95% CI: 5·8–28·9]) and from 1990 to 2010 for distal colon cancer (8·6% [6·1–20·5]). A 6–7% per annum increase began over a decade later for 30–39-year-olds. A 2% (95% CI: 1·1–4·2) increase in distal colon cancer rates was detected among 40–49-year-olds since 2003, but no increases were observed for proximal colon cancer. Among those aged ≥50, multiple periods of decreasing APC were found. Qualitative inspection of the point estimates and 95% CIs suggested that decreases may be larger for distal than proximal colon cancer across comparable periods, though no formal test of a statistical difference was performed because the estimates refer to different trend periods identified by joinpoint modelling. Since 1990, increasing AAPC resulted in 415 (95% UI: 243–646) and 1262 (95% UI: 844–1761) excess colon cancers among adults aged 20–29 and 30–39 years, respectively, but estimates overlapped 0 for those aged 40–49 years (Table 2). These estimates correspond to 0·4 and 1·3 cases per 100,000 person-years or a 79·2% and 51·3% relative increase in observed compared to expected numbers under no rate increase. Results were similar between men and women and in secondary analyses that included neuroendocrine neoplasms (Appendix Tables S3–S5, S11–S13).

**Table 2:** Cumulative excess early-onset cancers attributable to rising incidence rates between 1990 and 2020.

Age, years	AAPC (95% CI)	Recorded cases	Expected cases (95% UI)^a^	Excess cases (95% UI)	AER (95% UI)^b^	P-score (%, 95% UI)
**Appendix**						
20–29	4·4 (−0·3, 9·5)	81	49 (0, 82)	32 (−1, 81)	0·0 (0·0, 0·1)	66·1 (−1·8, Inf)
30–39	7·4 (5·1, 9·7)	244	62 (0, 149)	181 (95, 244)	0·2 (0·1, 0·3)	290·5 (63·9, Inf)
40–49	6·8 (4·7, 9·1)	611	187 (0, 389)	424 (222, 611)	0·5 (0·2, 0·7)	226·4 (57·2, Inf)
Total	–	936	298 (0, 620)	638 (316, 936)	0·2 (0·1, 0·3)	213·6 (51·0, Inf)
**Colon**						
20–29	3·5 (2·4, 4·7)	939	524 (293, 696)	415 (243, 646)	0·4 (0·3, 0·7)	79·2 (34·9, 220·2)
30–39	2·6 (1·9, 3·3)	3725	2463 (1964, 2881)	1262 (844, 1761)	1·3 (0·9, 1·8)	51·3 (29·3, 89·7)
40–49	−0·3 (−0·7, 0·2)	11,778	12,326 (11,414, 13,112)	−548 (−1334, 364)	−0·6 (−1·5, 0·4)	−4·4 (−10·2, 3·2)
Total	–	16,442	15,313 (13,671, 16,689)	1129 (−247, 2771)	0·4 (−0·1, 1·0)	7·4 (−1·5, 20·3)
**Rectum**						
20–29	4·5 (2·9, 6·2)	519	244 (53, 369)	275 (150, 466)	0·3 (0·2, 0·5)	113·0 (40·5, 878·4)
30–39	3·0 (2·5, 3·5)	2618	1605 (1358, 1822)	1013 (796, 1260)	1·1 (0·8, 1·3)	63·1 (43·7, 92·8)
40–49	0·5 (0·2, 0·8)	8686	8025 (7527, 8455)	661 (231, 1159)	0·7 (0·3, 1·3)	8·2 (2·7, 15·4)
Total	–	11,823	9874 (8938, 10,646)	1949 (1177, 2885)	0·7 (0·4, 1·0)	19·7 (11·1, 32·3)
**Anus**						
30–39	−0·2 (−1·8, 1·4)	251	259 (186, 309)	−8 (−58, 65)	0·0 (−0·1, 0·1)	−3·2 (−18·8, 34·9)
40–49	2·6 (1·6, 3·6)	1055	691 (484, 852)	364 (203, 570)	0·4 (0·2, 0·6)	52·7 (23·9, 117·6)
Total	–	1306	950 (670, 1161)	356 (145, 635)	0·2 (0·1, 0·3)	37·5 (12·5, 94·6)

Note: Excludes neuroendocrine neoplasms. Estimates for all histotypes combined and by sex are provided in Appendix Table S11–S13.

CI, confidence interval; AAPC, average annual percentage change; UI, uncertainty interval; AER, absolute excess risk.

a Expected cases with an AAPC of 0% from 1990.

b Per 100,000 persons-years.

For rectal cancer, IRs increased per annum among 20–29-year-olds until 2013 (APC 6·7% [95% CI: 5·2–9·4]) (Fig. 1, Table 1). Similar increases were detected in 30–39-year-olds from 2015. A moderate linear increase was observed among 40–49-year-olds since 1990 (APC 0·5% [95% CI: 0·2–0·8]). Conversely, rates generally decreased by approximately 4% per annum over the past decade among those aged ≥50. Since 1990, increasing incidence caused 1949 (95% UI: 1177–2885) excess early-onset cases, equivalent to 0·7 per 100,000 person-years or a 19·7% relative increase. Results were similar between men and women but inflated when including neuroendocrine neoplasms (Appendix Tables S3–S5, S11–S13).

Appendiceal cancer (excluding neuroendocrine neoplasms) displayed linear increases for all age groups except 20–29-year-olds, where increases were only observed when including neuroendocrine neoplasms (Table 1, Appendix Table S3). Since 1990, increasing rates resulted in 2348 (95% UI: 1667–3256) and 638 (316–936) excess early-onset tumours with and without including neuroendocrine neoplasms, equivalent to 0·8 and 0·2 cases per 100,000 person-years or a 191·2% and 213·6% relative increase, respectively (Table 2, Appendix Table S12). Results were similar between men and women (Appendix Tables S4, S5, S11, S13).

Anal cancer incidence increased for all age groups 40–79 years, but no trends were detected among other ages (Fig. 1, Table 1). Since 1990, the AAPC in early-onset anal cancer rates was only positive among 40–49-year-olds (2·6% [95% CI: 1·6–3·6]) (Table 2). This resulted in 364 (95% UI: 203–570) excess cases, equivalent to 0·4 per 100,000 person-years (52·7% relative increase). Results were similar between men and women, although point estimates for the AER were approximately 2-fold higher in women compared to men (Appendix Tables S4, S5, S11, S13).

The age-standardised proportion of histotypes among cases by tumour site and early-onset versus later-onset (age ≥50 years) diagnosis is presented in Figs. S6 and S7. Only few tumours were classified as unknown histology, and their relative contribution remained stable over the study period. Heterogeneity of known histological subtypes by age was most noticeable for appendiceal tumours, with neuroendocrine neoplasms accounting for most early-onset but only about half of later-onset cases. For colorectal tumours, signet ring cell carcinomas and neuroendocrine neoplasms were more frequent in early-onset cases. For anal cancers, adenocarcinomas comprised a larger share of older-onset cases. Joinpoint trends were consistent when restricting to dominant histotypes, but early-onset colorectal mucinous adenocarcinoma incidence declined or stabilised (Appendix pp 10–14 and 25–30).

**Fig. 2:**
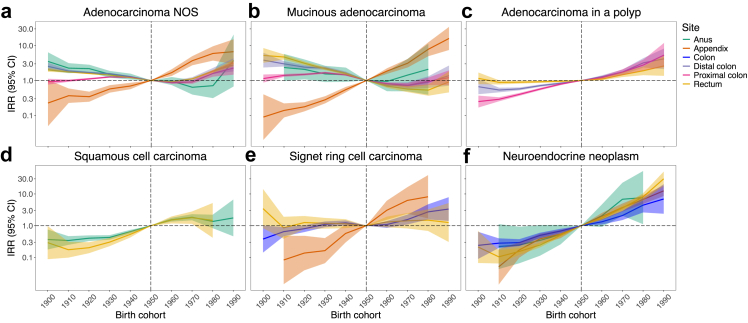
Birth cohort trends in tumour incidence by anatomical site and histology from 1900 to 1990. For each cancer site, age-period-cohort models were fitted to incidence rates from 1990 to 2019 of mutually-exclusive histological subtypes **a**: adenocarcinoma NOS; **b**: mucinous adenocarcinoma; **c**: adenocarcinoma in a polyp; **d**: squamous cell carcinoma; **e**: signet ring cell carcinoma; and **f**: neuroendocrine neoplasm. Shaded areas indicate 95% CIs. The vertical line represents the referent 1950 birth cohort. Neuroendocrine neoplasms and signet ring cell carcinomas of distal, proximal, and overlapping/unspecified colon sites were combined into a single ‘Colon’ category due to small case numbers. Sex-specific results are given in Appendix pp 34–35. NOS, not otherwise specified; IRR, incidence rate ratio; CI, confidence interval.

Fig. 2 summarises birth cohort trends for tumour histotypes. IRRs overall and by sex are in Appendix pp 34–37. Compared to those born in the 1950s, IRRs increased with birth cohort for all subtypes except anal adenocarcinoma, colorectal mucinous adenocarcinoma, and rectal signet ring cell carcinoma. Appendiceal tumours exhibited the steepest increases. For adenocarcinoma NOS, IRs in the 1990s versus 1950s were 6·7-fold for appendix and 2–3-fold for colorectum; similar trends appeared for colorectal adenocarcinoma in a polyp. Mucinous adenocarcinoma only increased within appendix; signet ring cell carcinoma increased within appendix and colon. Squamous cell carcinoma rose similarly across rectum and anus. Neuroendocrine neoplasms exhibited the largest increase across sites. Trends were consistent between men and women.

**Fig. 3:**
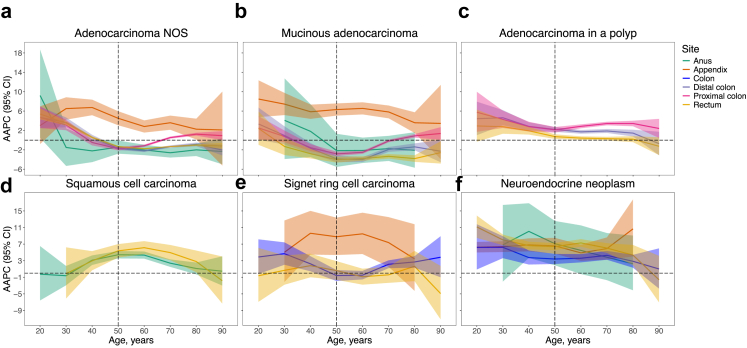
Age-specific AAPC in tumour incidence by anatomical site and histology from 1990 to 2019. For each cancer site, age-period-cohort models were fitted to age-specific incidence rates, from which the AAPC was estimated using the slope of the incidence rate ratio curves for mutually-exclusive histological subtypes **a**: adenocarcinoma NOS; **b**: mucinous adenocarcinoma; **c**: adenocarcinoma in a polyp; **d**: squamous cell carcinoma; **e**: signet ring cell carcinoma; and **f**: neuroendocrine neoplasm. Shaded areas indicate 95% CIs. The vertical line at 50 years indicates the cut-off for young adults. Neuroendocrine neoplasms and signet ring cell carcinomas of distal, proximal, and overlapping/unspecified colon sites were combined into a single ‘Colon’ category due to small case numbers. Sex-specific results are given in the Appendix pp 37–39. NOS, not otherwise specified; AAPC, average annual percentage change; CI, confidence interval.

The age-specific AAPC for histological subtypes estimated from the slope of the IRR curves is given in Fig. 3, and Appendix pp 37–40. Trends in increasing incidence were greatest for appendiceal tumours across all ages. Other subtype-specific trends mirrored birth cohort IRR patterns, with subtypes showing rising incidence since the 1950's birth cohort also exhibiting positive AAPCs among adults under age 40 years from 1990 to 2020. Tumours that increased in young and older age groups and did not conflict with recent joinpoint trends included appendiceal tumours, neuroendocrine neoplasms (all sites), rectal and anal squamous cell carcinoma, and colon signet ring cell carcinoma. For early-onset colorectal adenocarcinoma NOS, trends were similar across subsites and highest among adults aged <40 (AAPC 3–5%). Only colorectal adenocarcinoma NOS, colorectal mucinous adenocarcinoma, and anal adenocarcinoma rates declined among populations older than 50 (AAPC −1 to −4%).

## Discussion

This population-based time-series offers the most comprehensive and up-to-date estimates of appendiceal, colorectal, and anal cancer incidence trends in Australia. While colorectal adenocarcinoma rates declined or stabilised in adults over age 50, they continue to rise in younger adults, although this pattern is not observed for other histotypes. Anal and rectal squamous cell carcinomas exhibit similar increases that peak around middle age. Our findings of birth cohort effects highlight increasing risks for most histotypes since 1960. Across all ages, increases were most pronounced for neuroendocrine neoplasms and appendiceal tumours. Despite large relative increases, the absolute impact remains modest, with 1–2 excess cases per 100,000 person-years from 1990 to 2020 primarily driven by rectal cancer.

The age-related colorectal cancer trends in this study corroborate previous findings in Australia.^2^^,^^3^ We further demonstrate that divergent trends between early-onset and later-onset cases are specific to conventional adenocarcinoma (non-mucinous, non-signet ring cell). This has also been reported in North America,^13^^,^^14^ Germany,^15^ and Norway.^16^ This has implications for screening, as adenocarcinomas arise from precancerous polyps through well-described natural history and are the target of population screening, introduced for ages 50–74 in early 2000s.^2^^,^^3^ This likely explains declining rates among older adults, but it remains unclear whether early-onset adenocarcinomas represent a continuum of older-onset disease driven by earlier risk factor exposure.

Enrichment of mucinous and signet ring cell histology among early-onset colorectal adenocarcinoma is well-documented.^7^^,^^17^ This has fuelled conjecture that these are hallmarks of the rising incidence, and that causes for the increase could be causes of mucinous and signet ring cell differentiation.^7^^,^^17^ Our findings challenge this by notion demonstrating that rates of early-onset colorectal mucinous adenocarcinoma have stabilised since 1990, and both younger and older adults exhibit similar increases in colonic signet ring cell carcinoma. The former observation is also reported in Norway^16^ and Canada.^13^ This suggests that causes of the rising incidence are unlikely to be the same as causes of early-onset colorectal cancer *per se*. Increasing signet ring cell carcinoma of the colon, but not rectum, is also reported in South Korea,^18^ while stable rates are reported in USA.^19^ Reasons behind these trends remain uncertain but warrant further investigation.

Our findings also challenge the notion that the rise in early-onset colorectal cancer is driven by distal, but not proximal, colorectal cancer.^7^ Although this trend is reported in Norway,^16^ Germany,^15^ and North America,^13^ data from England and Australia^20^ suggest increases of similar magnitude across subsites at younger ages, consistent with our findings. Since these geographical discrepancies are observed after using homogenous subsite classifications,^20^ one could postulate differing prevalence of risk factors that indiscriminately promote carcinogenesis across countries.

Our study illustrates the importance of stratifying by appendiceal involvement, given the heterogeneity in incidence. After excluding appendix, our estimates of early-onset colon cancer rates were smaller than previous studies that classified appendix as colon.^2^^,^^3^ Within appendiceal cancer, there is heterogeneity in the frequency and clinical behaviour of histotypes, from indolent neuroendocrine neoplasms in young adults, to advanced older-onset adenocarcinomas and rare but aggressive signet ring cell carcinomas. We found that all of these subtypes are increasing at magnitudes higher than any other anatomical site. This may reflect a global phenomenon, with the same observations reported in North America^19^^,^^21^ and Europe.^15^

Whether the rise in appendiceal cancer represents an aetiological phenomenon is unknown. Appendiceal tumours are typically incidental discoveries in 2% of appendectomy specimens for suspected appendicitis.^22^ From 2000 to 2013, Australia doubled availability to computerised tomography scans, and rates of appendectomy rose 25%,^23^ which suggests that a portion of our estimates may reflect overdiagnosis rather than changes in underlying tumour biology. Although we could not assess this, increasing appendiceal cancer rates are reported for all disease stages in North America, after accounting for appendectomies.^21^ Well-differentiated appendiceal neuroendocrine neoplasms were reclassified as malignant by cancer registries around 2010; however, since rates were increasing from 1990, an aetiological explanation seems plausible. One hypothesis is a shift toward managing appendicitis with antibiotics instead of appendectomy, which may promote carcinogenesis via chronic inflammation.^22^

Neuroendocrine neoplasms exhibited steep increases across all ages and tumour sites, and their inclusion inflated appendiceal and rectal cancer trends. Therefore, increases in early-onset colorectal cancer reported by previous studies that pooled histological subtypes were likely driven, in part, by neuroendocrine neoplasms.^2^^,^^3^^,^^8^ Given the absence of age-specific patterning, the increase is unlikely to reflect overdiagnosis: colorectal neuroendocrine neoplasms are typically incidental discoveries on colonoscopy^14^^,^^16^; therefore, one would expect the rise to be exclusive to older populations if driven by screening alone. Rather, the rise may reflect changing classification. In 2000/2004, the World Health Organisation introduced a new classification system for gastrointestinal neuroendocrine neoplasms, and the 2010 edition resulted in most carcinoids being upgraded to grade 1 neuroendocrine neoplasms, which may be artefactually inflating rates.^8^

The rising incidence of anal squamous cell carcinoma but not adenocarcinoma mirrors trends across high-income countries, following an inverse U-shaped distribution peaking around middle age.^9^ Although site misclassification cannot be excluded, the similar trajectory for rectal squamous cell carcinoma might indicate shared aetiology. Human papillomavirus (HPV) is present in 80–90% of anorectal squamous cell carcinomas, but few adenocarcinomas.^24^ Over the last 20 years, incidence of other HPV-related anogenital cancers has increased, and risk factors for anorectal HPV infection have become more prevalent, including lower mean age at first intercourse, receptive anal intercourse, and greater number of sexual partners.^24^

The generational shift in gastrointestinal cancer risk, suggested by birth cohort trends, is consistent with earlier risk factor exposure among generations born after 1960. Such factors are likely to selectively increase susceptibility to tumour subtypes rising in incidence. Sex-related factors may not be critical, as we and others found no prominent difference in trends between men and women.^3^ Non-communicable diseases like inflammatory bowel disease, diabetes, metabolic syndrome, and childhood obesity are suspected, given strong associations with early-onset colorectal cancer risk and increasing prevalence in Australia.^17^^,^^25^, ^26^, ^27^, ^28^ Meanwhile, lifestyle risk factors for tumour subtypes with increasing incidence include physical inactivity, alcohol abuse, low fruit intake, and high fast food consumption, all of which have risen in Australia over time.^17^^,^^26^^,^^29^

The effect of other suspected early-life risk factors, and trends in their prevalence, remains poorly understood. The constellation of gastrointestinal tract cancers increasing in recent birth cohorts highlights potential involvement of an altered gut microbiome, supported by changes to dietary patterns and increased antibiotic use in childhood.^17^^,^^26^ Perfluoroalkyl and polyfluoroalkyl substances (PFAS) are man-made fluorinated chemical carcinogens widely used in consumer products since 1950,^30^ a clear inflection point for birth cohort trends in the present study. PFAS are present at unsafe levels in tap water across Australia and have been linked to various gastrointestinal cancers.^30^ Other suspected early-life risk factors based on case–control evidence include bottle feeding, caesarean section, vitamin A, and maternal obesity.^17^^,^^26^ Moving forward, lifecourse studies are required to understand birth cohort-specific risk factor exposures and age-specific trajectories of susceptibility to tumour subtypes.

Our findings have several public health implications. Increasing incidence in younger generations foreshadow future disease burden as young cohorts carry elevated risk into older age. In future, this may tip the benefit-to-harm ratio of screening younger people, which is not currently justified due to low yield. Thus, improving awareness about risk factors, signs, and symptoms remains the primary opportunity for prevention. Moreover, the rising incidence of squamous cell carcinoma, signet ring cell carcinoma, and neuroendocrine neoplasms has implications for screening as incidence continues to rise across both young and older groups. In particular, further research should explore whether these trends identify disease subtypes that warrant prioritisation in subgroup analyses of screening effectiveness, risk quantification, and modelling studies. Screening policy decisions ultimately require integration of incidence trends with evidence on mortality, cost-effectiveness, participation, and risk-benefit balance; our results highlight areas where such analyses may be valuable.

The strengths of this study include nationally representative and complete longitudinal cancer records; systematic evaluation of multiple anatomical sites, including the separation of appendiceal and proximal colon cancers; and stratification by histological subtype. In addition, birth cohort analyses provide more robust assessment of trends that may be related to changing risk factor prevalence. Collectively, this enabled detection of age-related differences not previously apparent.

Several limitations should be considered. We could not disaggregate the impact of changing diagnostic practices, and we lacked information about clinical contexts surrounding diagnosis. Therefore, further research assessing trends by stage and detection method is needed to help disentangle the effect of changing generational risk versus diagnostic scrutiny. Additionally, country-level analyses could mask disparate trends across sociodemographic groups, necessitating further studies to identify high-risk subpopulations. Our findings are based on IR trends, and we did not estimate absolute lifetime or 10-year risks or conduct projections; therefore, the results cannot be used to directly inform screening recommendations. Lastly, small case numbers for rare histological subtypes, combined with the large number of 10-year age-specific categories, produced unstable estimates in some analyses and made interpretation more challenging, necessitating careful consideration of results.

In conclusion, early-onset appendiceal, colorectal, and anal cancer incidence is rising in Australia with variation across age and histology. Overall, the largest number of excess early-onset cases occur in the rectum. Conventional colorectal adenocarcinoma rates have declined or stabilised in adults over age 50 but continue to rise in younger adults, potentially due to opposing influences of risk factors and screening. Anal and rectal squamous cell carcinomas exhibit similar increases that peak around middle age, likely due to HPV infection. Across all ages, increases are most pronounced for neuroendocrine neoplasms and appendiceal tumours, although the role of changing diagnostic practices and risk factor exposure remains unknown. Each successive generation born during the second half of the 20th century experienced elevated risk of lower gastrointestinal tumour subtypes with potential distinct and overlapping aetiologies, highlighting the importance of stratifying future studies by site and histology. Extensive efforts are needed to identify factors responsible for these trends to inform prevention strategies.

## Contributors

A.L. Meyers: Conceptualization, Methodology, Software, Formal analysis, Resources, Data curation, Writing – original draft, Writing – review & editing; J.G. Dowty: Supervision, Writing – review & editing; K. Mahmood: Supervision, Writing – review & editing; F.A. Macrae: Methodology, Writing – review & editing; C. Rosty: Methodology, Writing – review & editing; D.D. Buchanan: Conceptualization, Methodology, Supervision, Writing – review & editing; M.A. Jenkins: Conceptualization, Methodology, Resources, Supervision, Writing – review & editing. All authors had full access to all study data and had final responsibility for the decision to submit for publication. A.L. Meyers and M.A. Jenkins have accessed and verified the data.

## Data sharing statement

National population figures can be extracted via the Australian Bureau of Statistics website (https://www.abs.gov.au/statistics/people/population). All other study data and related documents are available upon request from the corresponding author, Professor Mark Jenkins, via email at m.jenkins@unimelb.edu.au, with support from the Australian Institute of Health and Welfare.

## Declaration of interests

Mark Jenkins has a fellowship that includes his salary and research support paid to the University of Melbourne. All other authors declare that they have no competing interests, financial or otherwise, concerning this work.
