# Supplementary material for: Age-specific trends in colorectal, appendiceal, and anal tumour incidence by histological subtype in Australia from 1990 to 2020: a population-based time-series analysis
Source: Lancet Reg Health West Pac. 2025 Oct 30;64:101728. doi: 10.1016/j.lanwpc.2025.101728 (PMC12641218; doi:10.1016/j.lanwpc.2025.101728)
Supplement: Supplementary File [file mmc1.pdf]

Supplementary appendix

Age-specific trends in colorectal, appendiceal, and anal tumour incidence by histological subtype in Australia from 1990 to 2020: a population-based time-series analysis

Table of contents

Supplementary figures ... 3

Figure S1: Period trends in tumour incidence for all histological subtypes combined by anatomical site and age from 1990 to 2020 ... 3

Figure S2: Period trends in tumour incidence for all histological subtypes combined in males by anatomical site and age from 1990 to 2020 ... 4

Figure S3: Period trends in cancer incidence for all histological subtypes combined excluding neuroendocrine neoplasms in males by anatomical site and age from 1990 to 2020 ... 5

Figure S4: Period trends in tumour incidence for all histological subtypes combined in females by anatomical site and age from 1990 to 2020 ... 6

Figure S5: Period trends in cancer incidence for all histological subtypes combined excluding neuroendocrine neoplasms in females by anatomical site and age from 1990 to 2020 ... 7

Figure S6: Age-standardised proportion of cases of each histological subtype among appendiceal and large bowel tumour cases in males by anatomical site and age from 1990 to 2020 ... 8

Figure S7: Age-standardised proportion of cases of each histological subtype among appendiceal and large bowel tumour cases in females by anatomical site and age from 1990 to 2020 ... 9

Figure S8: Period trends in appendiceal tumour incidence by histology and age from 1990 to 2020 ... 10

Figure S9: Period trends in proximal colon tumour incidence by histology and age from 1990 to 2020 ... 11

Figure S10: Period trends in distal colon tumour incidence by histology and age from 1990 to 2020 ... 12

Figure S11: Period trends in rectal tumour incidence by histology and age from 1990 to 2020 ... 13

Figure S12: Period trends in anal tumour incidence by histology and age from 1990 to 2020 ... 14

Supplementary tables ... 15

Table S1: Classification scheme for tumour histological subtypes. ... 15

Table S2: Characteristics of cancer registry data per annum. ... 15

Table S3: Temporal trends in tumour incidence rates by age, anatomical site, and calendar period of diagnosis for all histological subtypes combined. ... 18

Table S4: Temporal trends in cancer incidence rates by age, anatomical site, sex, and calendar period of diagnosis after excluding neuroendocrine neoplasms. ... 19

Table S5: Temporal trends in tumour incidence rates by age, anatomical site, sex, and calendar period of diagnosis for all histological subtypes combined. ... 22

Table S6: Temporal trends in appendiceal tumour incidence rates by age, histology, and calendar period of diagnosis. ... 25

Table S7: Temporal trends in proximal colon tumour incidence rates by age, histology, and calendar period of diagnosis. ... 26

Table S8: Temporal trends in distal colon tumour incidence rates by age, histology, and calendar period of diagnosis. ... 27

Table S9: Temporal trends in rectal tumour incidence rates by age, histology, and calendar period of diagnosis. ... 28

Table S10: Temporal trends in anal tumour incidence rates by age, histology, and calendar period of diagnosis. ... 30

Table S11: Cumulative excess early-onset cancers attributable to rising incidence rates between 1990 and 2020, by sex and after excluding neuroendocrine neoplasms. 30

Table S12: Cumulative excess early-onset tumours attributable to rising incidence rates between 1990 and 2020, for all histological subtypes combined. 32

Table S13: Cumulative excess early-onset tumours attributable to rising incidence rates between 1990 and 2020, by sex and for all histological subtypes combined. 33

Table S14: Ratio of age-specific cancer incidence rates between 1990 and 2019, by site, sex, and birth cohort after excluding neuroendocrine neoplasms. 34

Table S15: Ratio of age-specific tumour incidence rates between 1990 and 2019, by site, sex, and birth cohort for all histological subtypes combined. 34

Table S16: Ratio of age-specific tumour incidence rates between 1990 and 2019, by site, histology, and birth cohort. 35

Table S17: Average annual percentage change of expected age-specific cancer incidence rates between 1990 and 2019, by sex and site after excluding neuroendocrine neoplasms. 37

Table S18: Average annual percentage change of expected age-specific tumour incidence rates between 1990 and 2019, by sex and site for all histological subtypes combined. 38

Table S19: Average annual percentage change of expected age-specific tumour incidence rates between 1990 and 2019, by histology and site. 39

Supplementary figures

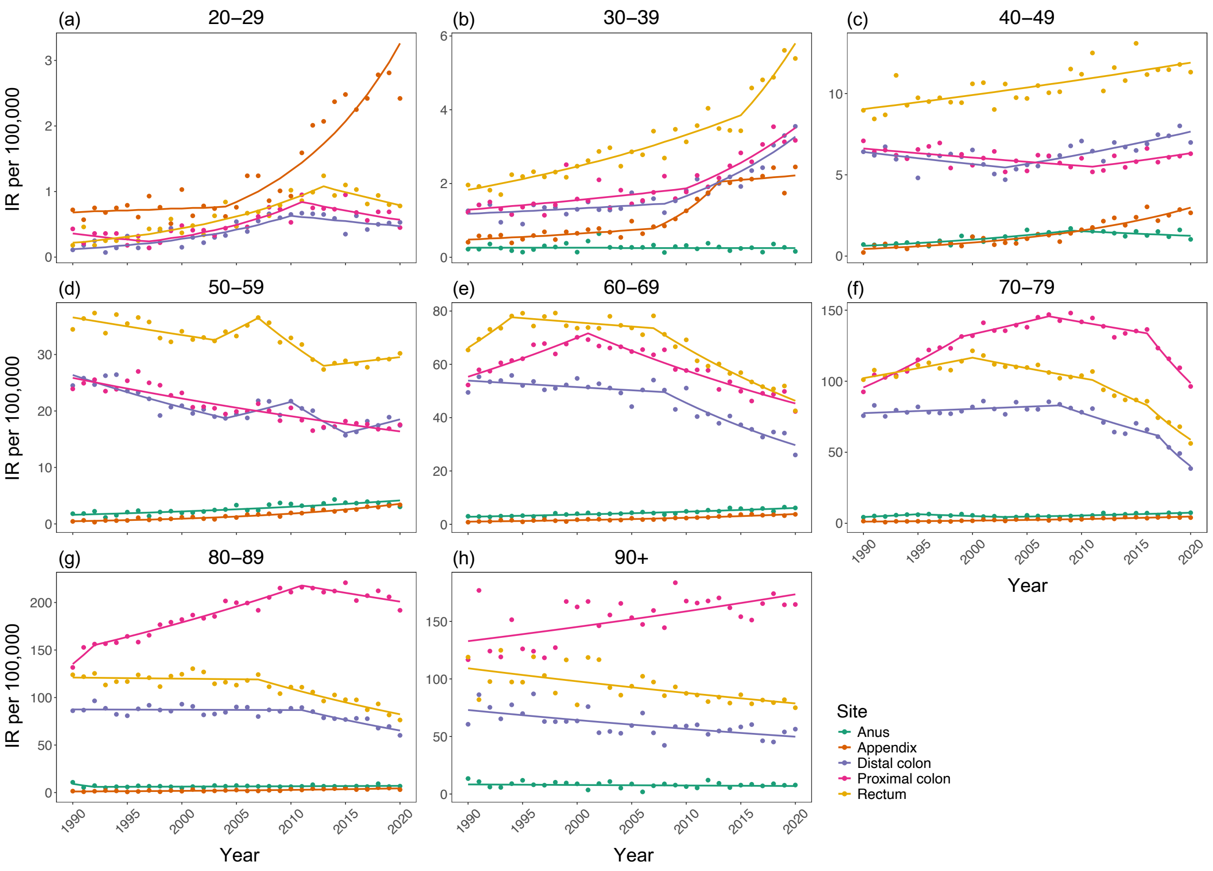

Figure S1: Period trends in tumour incidence for all histological subtypes combined by anatomical site and age from 1990 to 2020. Joinpoint regression was used to fit a series of joined straight lines to tumour rates per 100,000 Australians aged **a:** 20-29; **b:** 30-39; **c:** 40-49; **d:** 50-59; **e:** 60-69; **f:** 70-79; **g:** 80-89; and **h:** 90+ years. The optimal number of segments was based on the weighted Bayesian information criterion method and set at a maximum of five. Annual percentage changes and 95% confidence intervals in each segment are given in Table S3. IR, incidence rate.

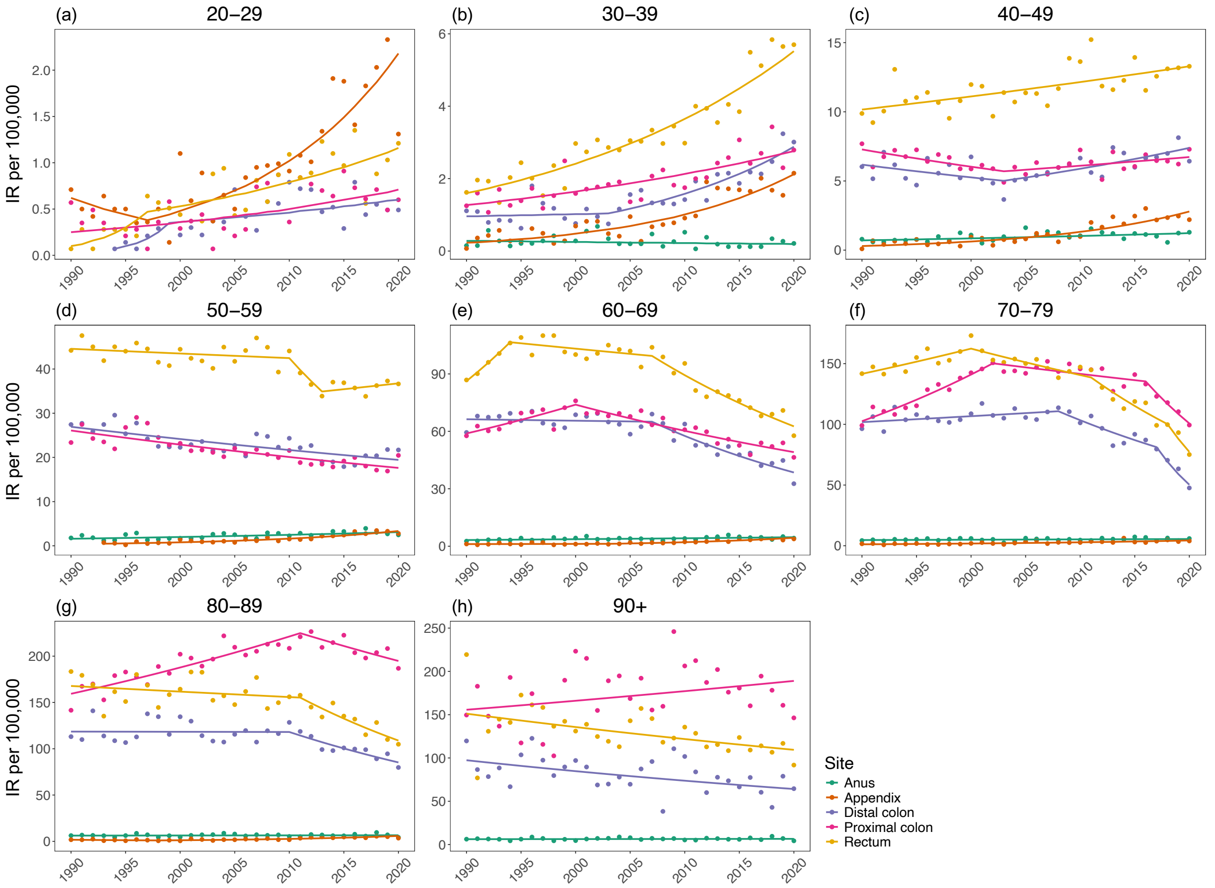

Figure S2: Period trends in tumour incidence for all histological subtypes combined in males by anatomical site and age from 1990 to 2020. Joinpoint regression was used to fit a series of joined straight lines to tumour rates per 100,000 Australian males aged **a:** 20-29; **b:** 30-39; **c:** 40-49; **d:** 50-59; **e:** 60-69; **f:** 70-79; **g:** 80-89; and **h:** 90+ years. The optimal number of segments was based on the weighted Bayesian information criterion method and set at a maximum of five. Annual percentage changes and 95% confidence intervals in each segment are presented in Table S5. IR, incidence rate.

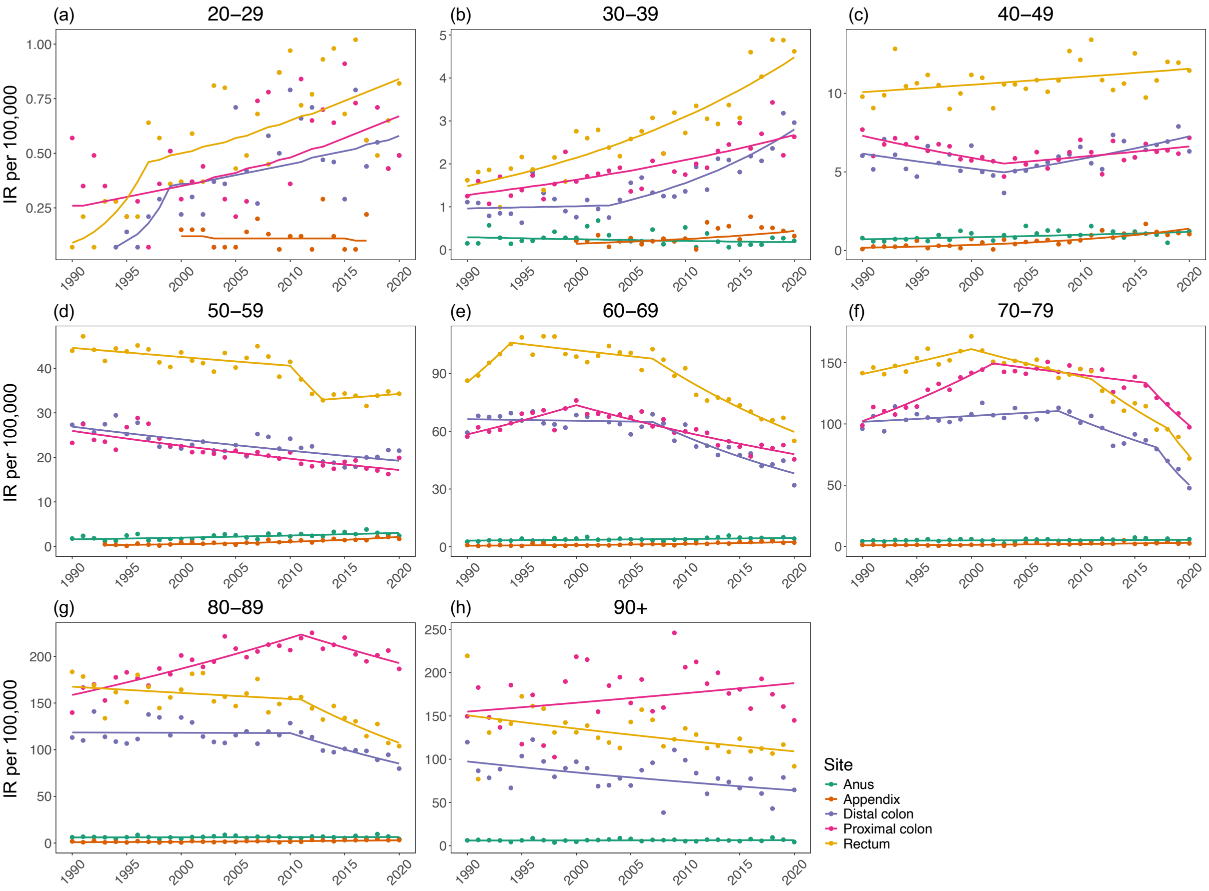

Figure S3: Period trends in cancer incidence for all histological subtypes combined excluding neuroendocrine neoplasms in males by anatomical site and age from 1990 to 2020. Joinpoint regression was used to fit a series of joined straight lines to cancer rates per 100,000 Australian males aged **a:** 20-29; **b:** 30-39; **c:** 40-49; **d:** 50-59; **e:** 60-69; **f:** 70-79; **g:** 80-89; and **h:** 90+ years. The optimal number of segments was based on the weighted Bayesian information criterion method and set at a maximum of five. Annual percentage changes and 95% confidence intervals in each segment are presented in Table S4. IR, incidence rate.

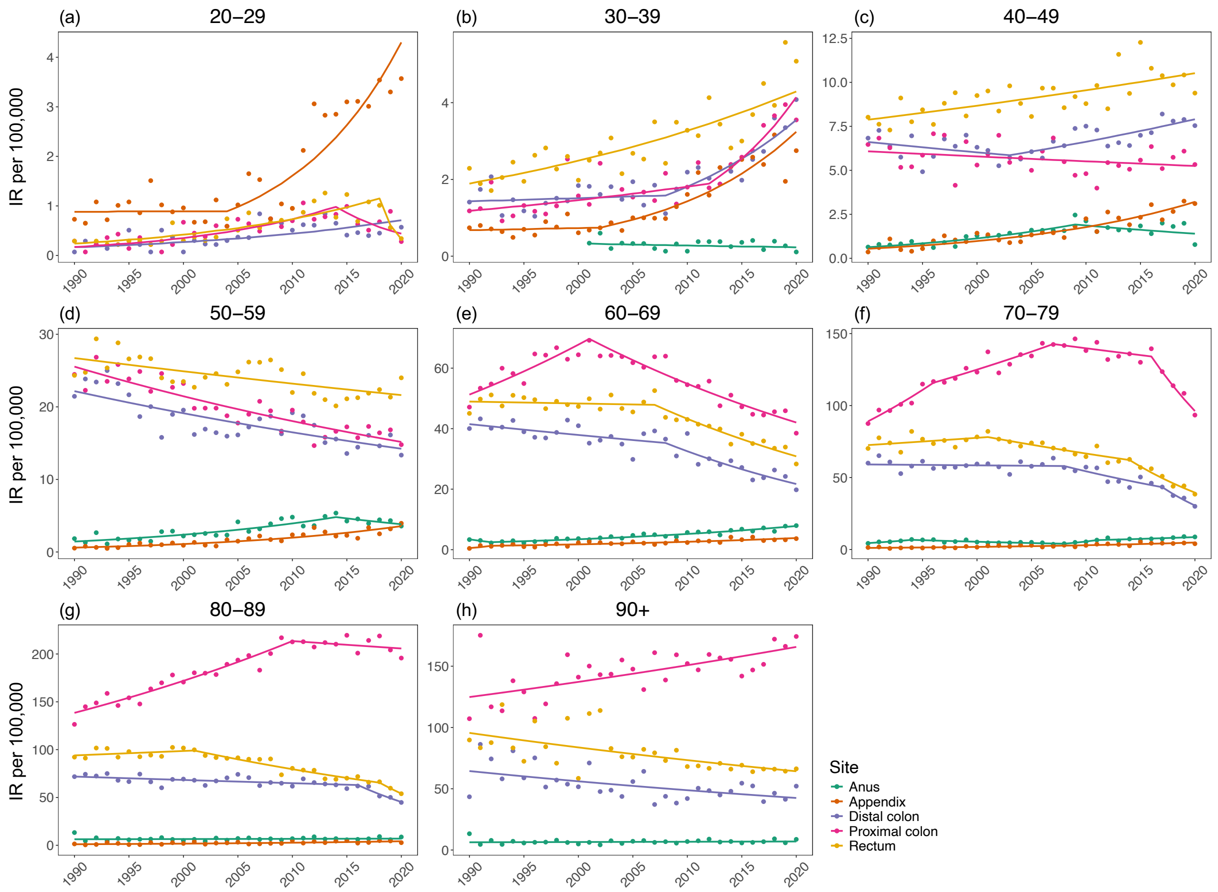

Figure S4: Period trends in tumour incidence for all histological subtypes combined in females by anatomical site and age from 1990 to 2020. Joinpoint regression was used to fit a series of joined straight lines to tumour rates per 100,000 Australian females aged **a:** 20-29; **b:** 30-39; **c:** 40-49; **d:** 50-59; **e:** 60-69; **f:** 70-79; **g:** 80-89; and **h:** 90+ years. The optimal number of segments was based on the weighted Bayesian information criterion method and set at a maximum of five. Annual percentage changes and 95% confidence intervals in each segment are presented in Table S5. IR, incidence rate.

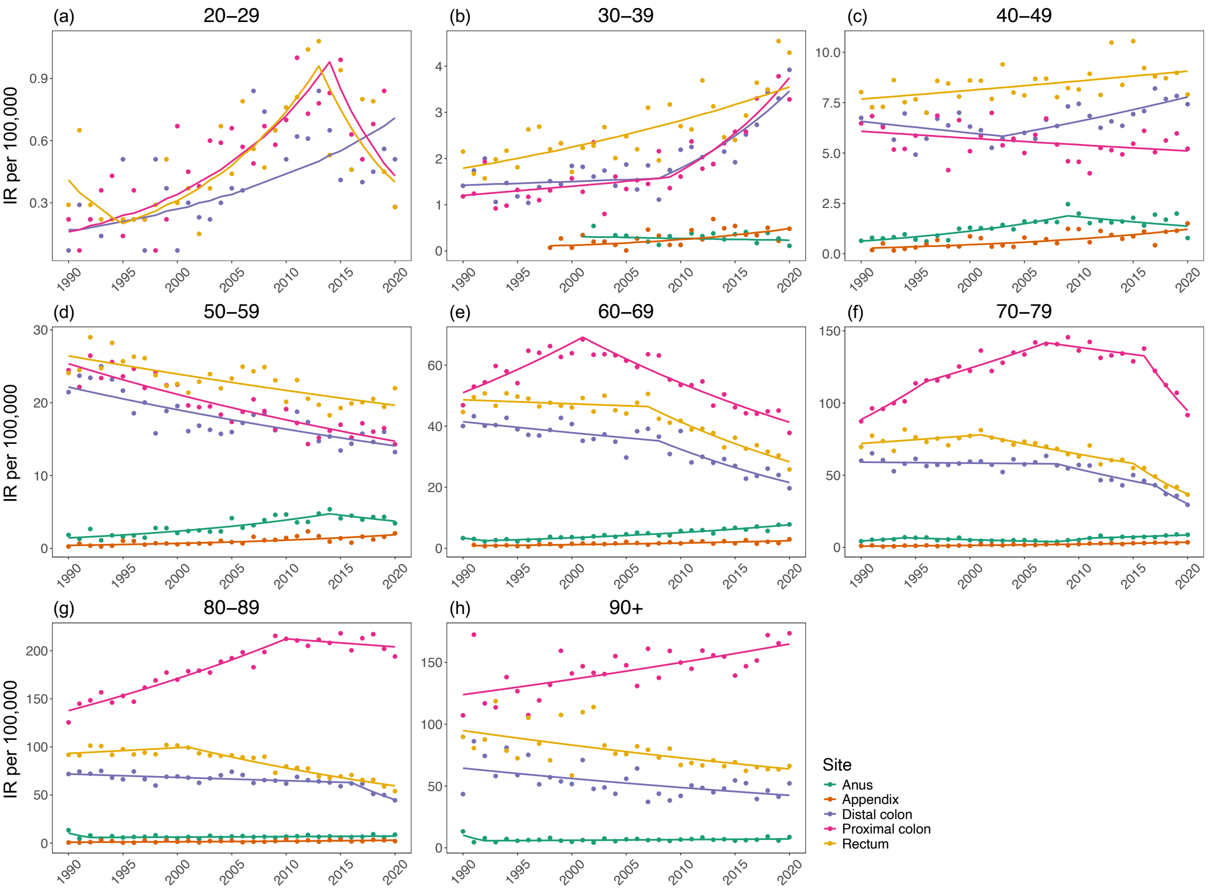

Figure S5: Period trends in cancer incidence for all histological subtypes combined excluding neuroendocrine neoplasms in females by anatomical site and age from 1990 to 2020. Joinpoint regression was used to fit a series of joined straight lines to cancer rates per 100,000 Australian males aged **a:** 20-29; **b:** 30-39; **c:** 40-49; **d:** 50-59; **e:** 60-69; **f:** 70-79; **g:** 80-89; and **h:** 90+ years. The optimal number of segments was based on the weighted Bayesian information criterion method and set at a maximum of five. Annual percentage changes and 95% confidence intervals in each segment are presented in Table S4. IR, incidence rate.

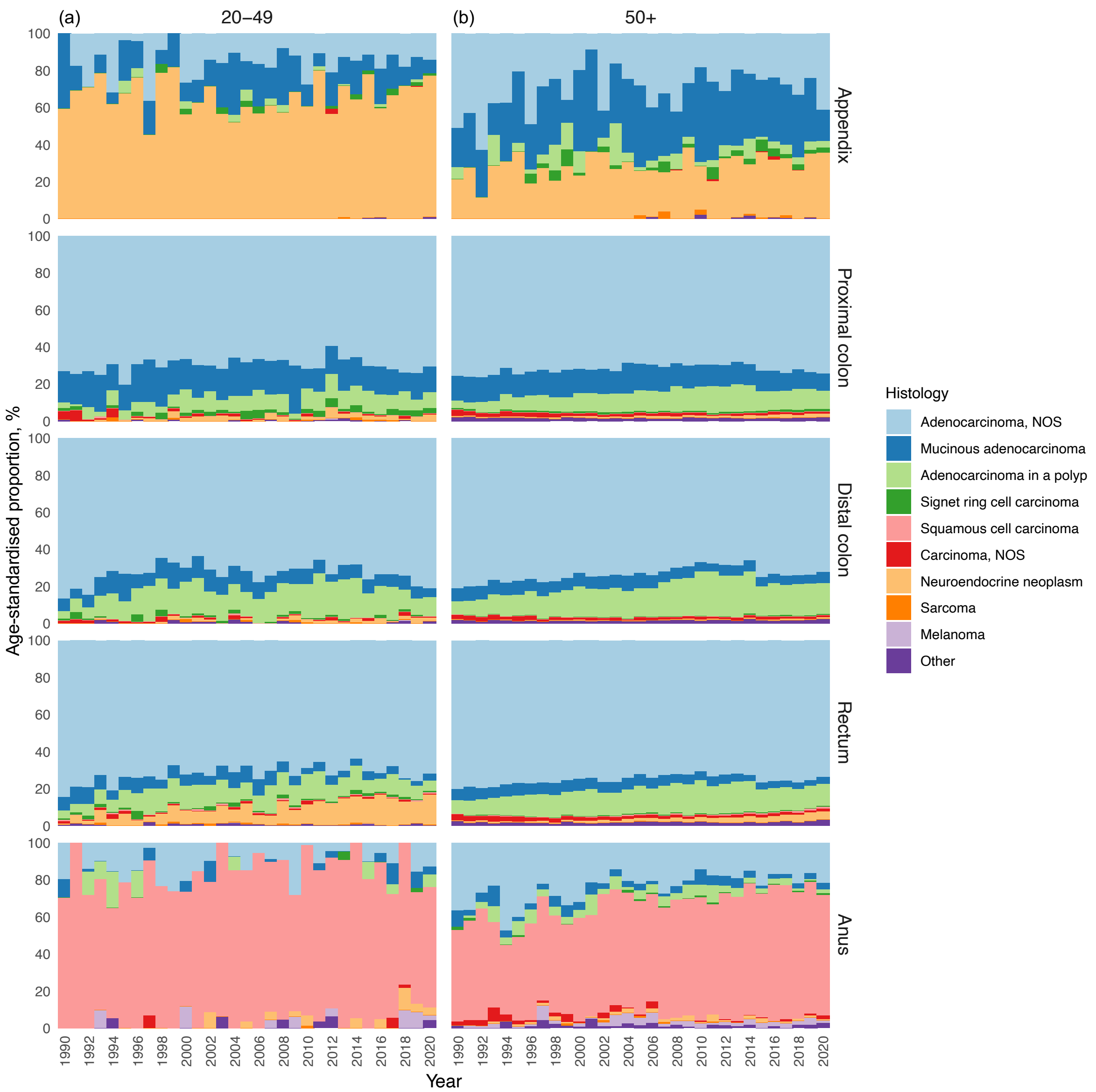

Figure S6: Age-standardised proportion of cases of each histological subtype among appendiceal and large bowel tumour cases in males by anatomical site and age from 1990 to 2020. Data are presented for **a:** early-onset (age 20-49 years) and **b:** later-onset (age 50+ years) cases, with direct age-standardisation to the corresponding 2005 case population. 'Other' includes unknown histologies.

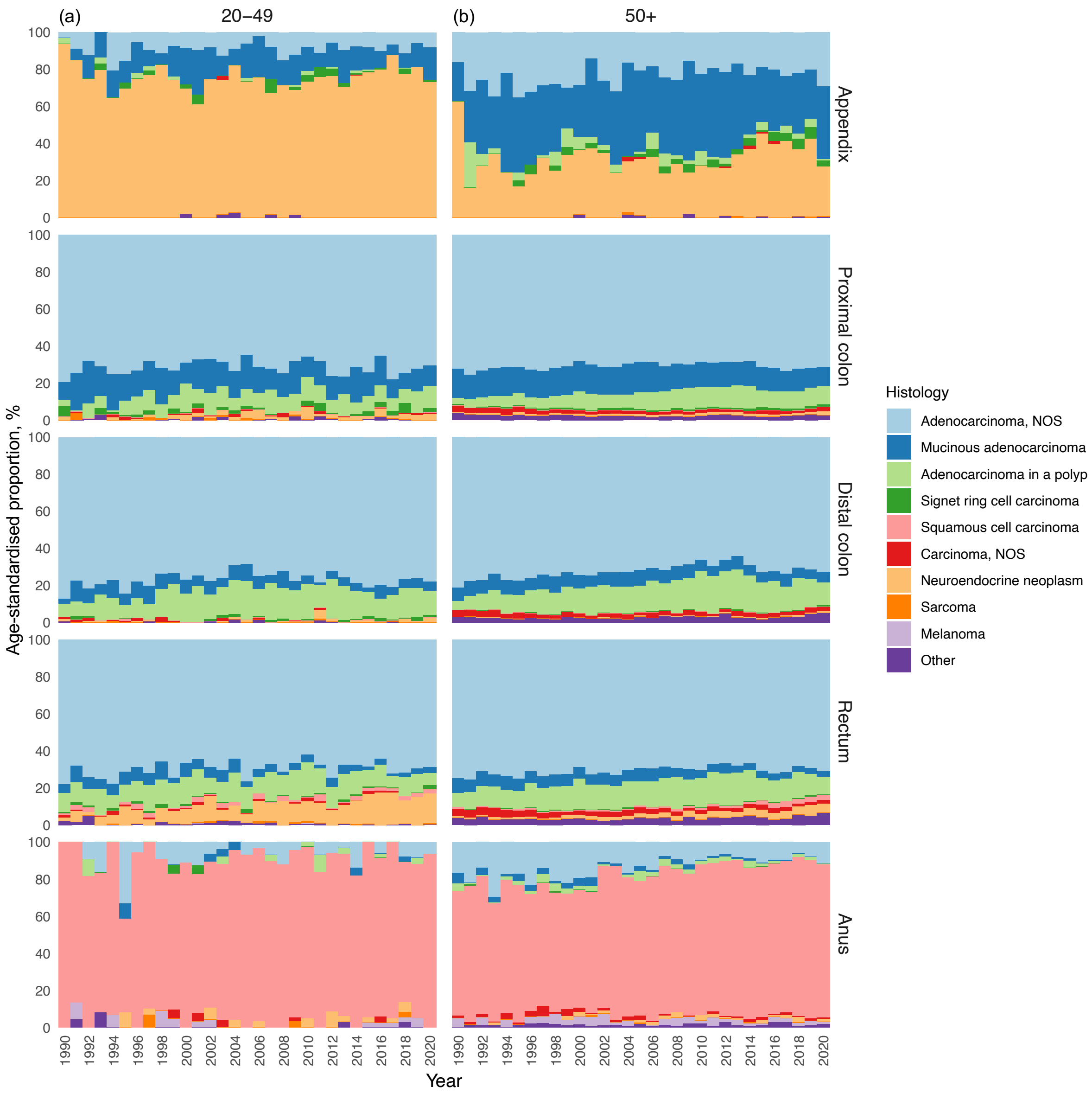

Figure S7: Age-standardised proportion of cases of each histological subtype among appendiceal and large bowel tumour cases in females by anatomical site and age from 1990 to 2020. Data are presented for **a:** early-onset (age 20-49 years) and **b:** later-onset (age 50+ years) cases, with direct age-standardisation to the corresponding 2005 case population. 'Other' includes unknown histologies.

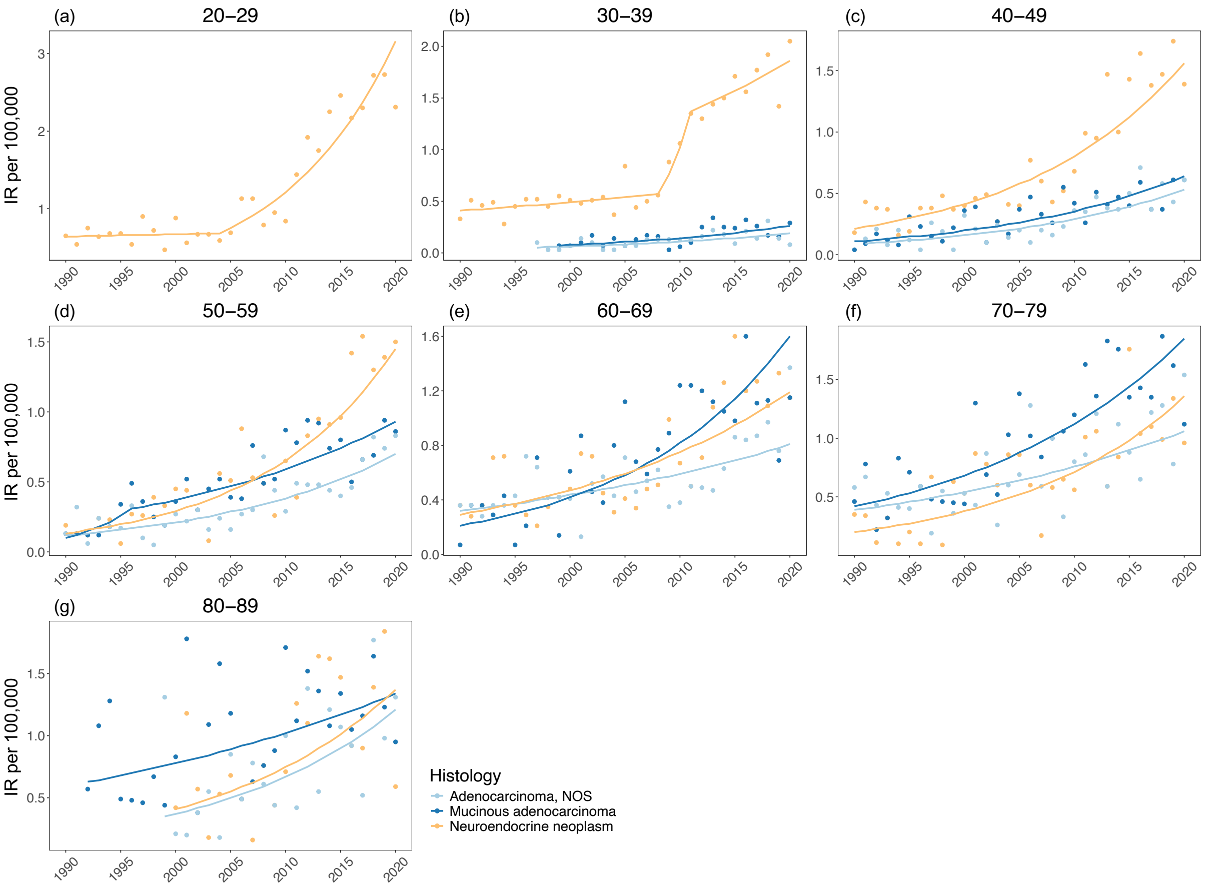

Figure S8: Period trends in appendiceal tumour incidence by histology and age from 1990 to 2020. Joinpoint regression was used to fit a series of joined straight lines to tumour rates per 100,000 Australians aged **a:** 20-29; **b:** 30-39; **c:** 40-49; **d:** 50-59; **e:** 60-69; **f:** 70-79; and **g:** 80-89 years. The optimal number of segments was based on the weighted Bayesian information criterion method and set at a maximum of five. Annual percentage changes and 95% confidence intervals in each segment are presented in Table S6. IR, incidence rate.

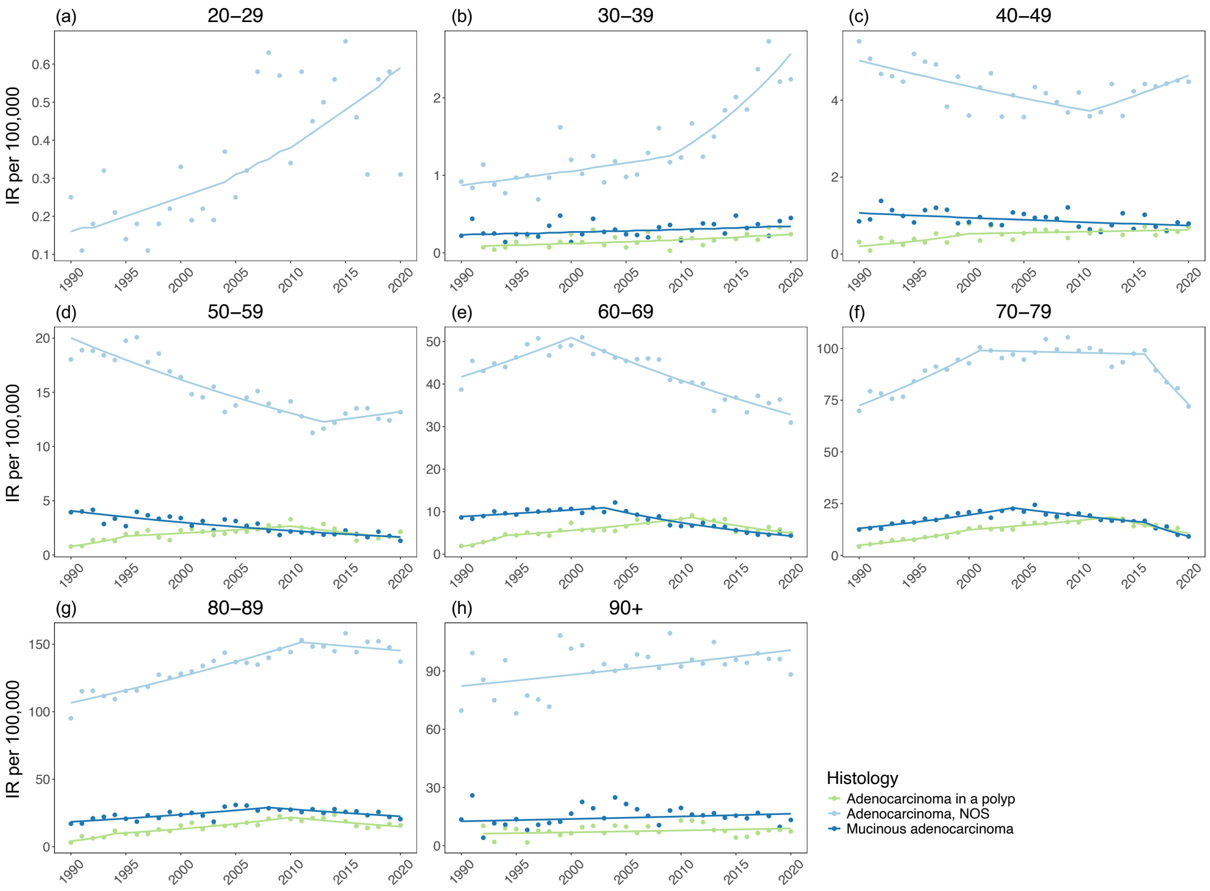

Figure S9: Period trends in proximal colon tumour incidence by histology and age from 1990 to 2020. Joinpoint regression was used to fit a series of joined straight lines to tumour rates per 100,000 Australians aged **a:** 20-29; **b:** 30-39; **c:** 40-49; **d:** 50-59; **e:** 60-69; **f:** 70-79; **g:** 80-89; and **h:** 90+ years. The optimal number of segments was based on the weighted Bayesian information criterion method and set at a maximum of five. Annual percentage changes and 95% confidence intervals in each segment are presented in Table S7. IR, incidence rate.

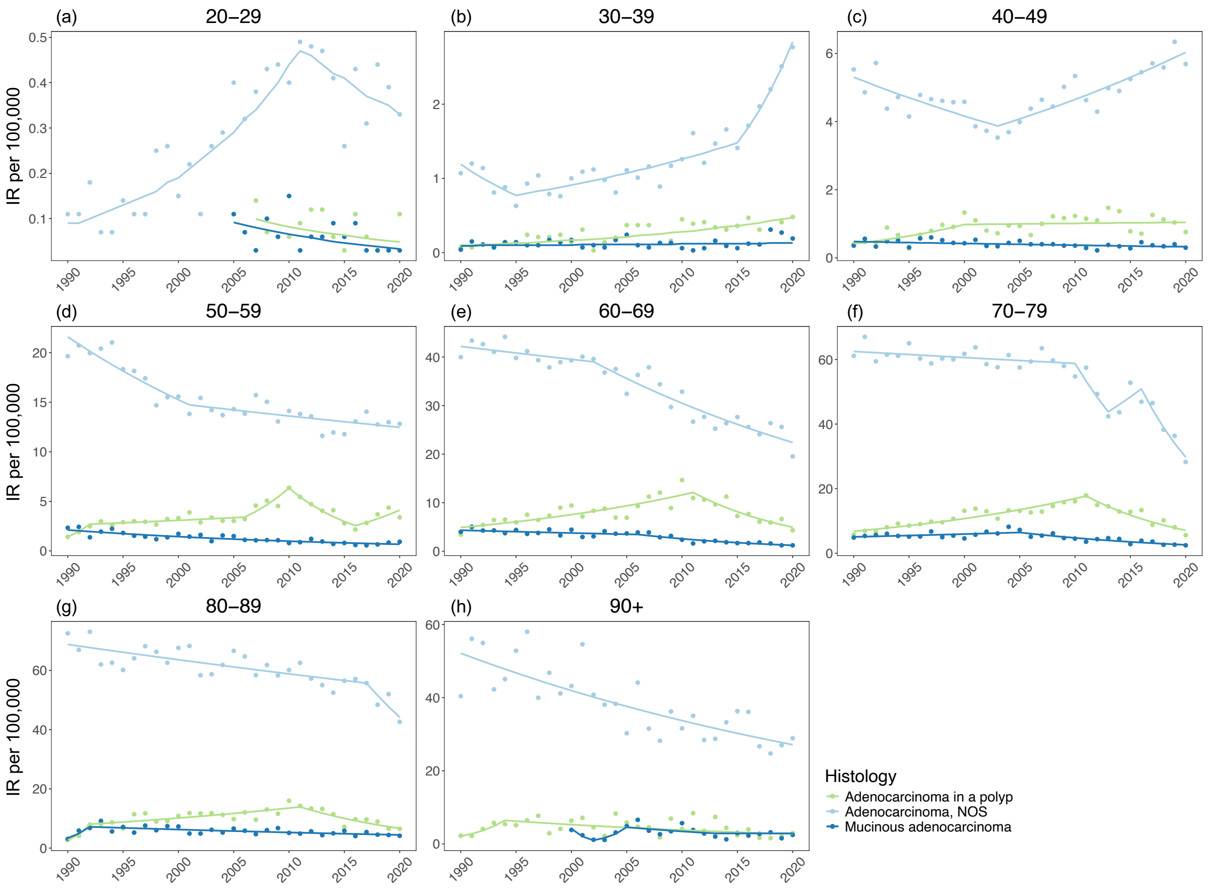

Figure S10: Period trends in distal colon tumour incidence by histology and age from 1990 to 2020. Joinpoint regression was used to fit a series of joined straight lines to tumour rates per 100,000 Australians aged **a:** 20-29; **b:** 30-39; **c:** 40-49; **d:** 50-59; **e:** 60-69; **f:** 70-79; **g:** 80-89; and **h:** 90+ years. The optimal number of segments was based on the weighted Bayesian information criterion method and set at a maximum of five. Annual percentage changes and 95% confidence intervals in each segment are presented in Table S8. IR, incidence rate.

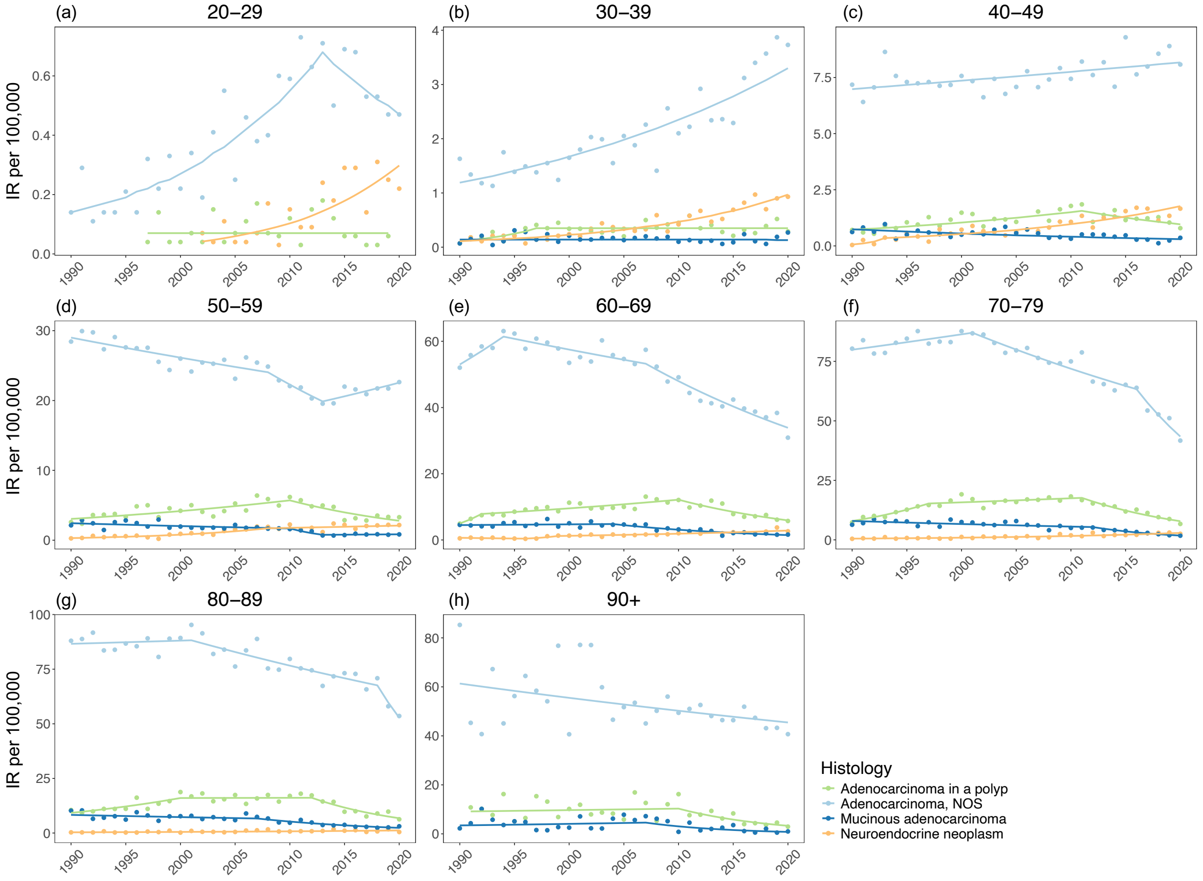

Figure S11: Period trends in rectal tumour incidence by histology and age from 1990 to 2020. Joinpoint regression was used to fit a series of joined straight lines to tumour rates per 100,000 Australians aged **a:** 20-29; **b:** 30-39; **c:** 40-49; **d:** 50-59; **e:** 60-69; **f:** 70-79; **g:** 80-89; and **h:** 90+ years. The optimal number of segments was based on the weighted Bayesian information criterion method and set at a maximum of five. Annual percentage changes and 95% confidence intervals in each segment are presented in Table S9. IR, incidence rate.

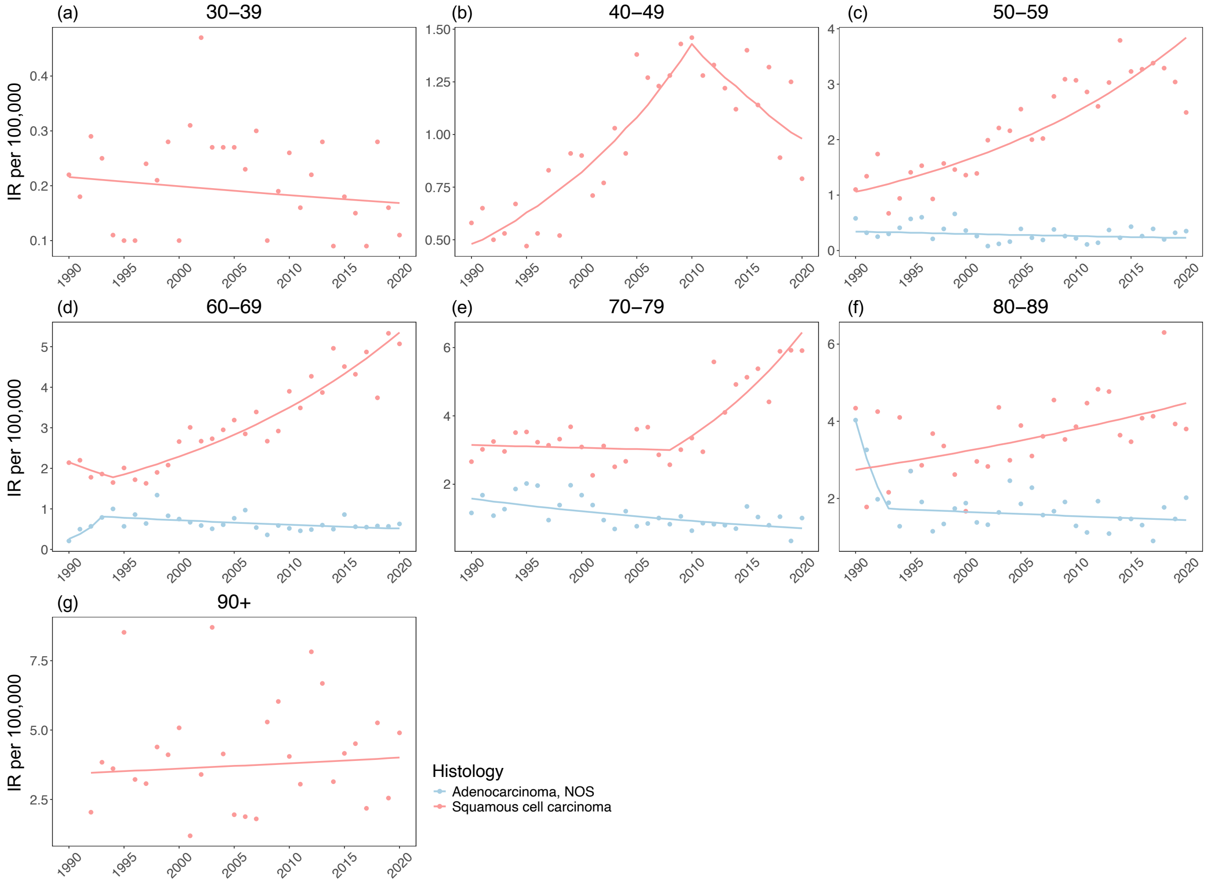

Figure S12: Period trends in anal tumour incidence by histology and age from 1990 to 2020. Joinpoint regression was used to fit a series of joined straight lines to tumour rates per 100,000 Australians aged **a:** 30-39; **b:** 40-49; **c:** 50-59; **d:** 60-69; **e:** 70-79; **f:** 80-89; and **g:** 90+ years. The optimal number of segments was based on the weighted Bayesian information criterion method and set at a maximum of five. Annual percentage changes and 95% confidence intervals in each segment are presented in Table S10. IR, incidence rate.

Supplementary tables

Table S1: Classification scheme for tumour histological subtypes.

Histological subtype	ICD-O-3 code
Adenocarcinoma NOS	8020, 8033, 8140-5, 8190, 8201, 8211, 8213, 8215, 8243, 8255, 8260, 8262, 8265, 8310, 8323, 8380, 8440, 8441, 8450, 8510, 8550, 8560, 8562, 8570, 8573, 8575
Mucinous adenocarcinoma	8470, 8472, 8480, 8481
Adenocarcinoma in a polyp	8210, 8221, 8261, 8263
Squamous cell carcinoma	8051, 8052, 8070-6, 8082, 8083, 8085, 8086, 8123, 8124
Signet ring cell carcinoma	8490
Neuroendocrine neoplasm	8013, 8041, 8044, 8045, 8154, 8240-2, 8244-6, 8249, 8574
Carcinoma NOS	8010, 8012, 8014, 8021, 8022, 8030-2, 8046, 8050, 8054, 8090, 8094, 8120, 8130, 8200, 8230, 8231, 8390, 8430, 8460, 8980, 9070, 9071, 9100
Sarcoma	8714, 8800-2, 8805, 8806, 8810, 8811, 8815, 8825, 8830, 8850, 8851, 8858, 8890, 8891, 8896, 8900, 8912, 8920, 8936, 8963, 9040, 9041, 9120, 9220, 9364, 9540, 9580
Melanoma	8720, 8721, 8730, 8743, 8746, 8770-2

NOS, not otherwise specified

Table S2: Characteristics of cancer registry data per annum.

Year	Age <50 years	Age ≥50 years

	Appendix	Proximal colon	Unknown / overlapping colon	Distal colon	Rectum	Anus	Appendix	Proximal colon	Unknown / overlapping colon	Distal colon	Rectum	Anus
**Persons**												
1990	36 (4.59)	204 (25.99)	82 (10.45)	181 (23.06)	259 (32.99)	23 (2.93)	36 (0.43)	2378 (28.73)	910 (10.99)	2031 (24.53)	2775 (33.52)	148 (1.79)
1991	47 (5.99)	194 (24.74)	71 (9.06)	188 (23.98)	262 (33.42)	22 (2.81)	44 (0.49)	2741 (30.38)	871 (9.66)	2261 (25.06)	2965 (32.87)	139 (1.54)
1992	55 (6.67)	208 (25.21)	63 (7.64)	208 (25.21)	264 (32.00)	27 (3.27)	33 (0.36)	2778 (30.60)	827 (9.11)	2220 (24.45)	3072 (33.84)	148 (1.63)
1993	50 (5.99)	200 (23.95)	52 (6.23)	176 (21.08)	329 (39.40)	28 (3.35)	53 (0.57)	2862 (30.99)	831 (9.00)	2303 (24.94)	3047 (33.00)	138 (1.49)
1994	43 (5.29)	194 (23.86)	53 (6.52)	190 (23.37)	305 (37.52)	28 (3.44)	52 (0.54)	2999 (30.91)	857 (8.83)	2350 (24.22)	3287 (33.88)	157 (1.62)
1995	53 (6.42)	213 (25.82)	54 (6.55)	159 (19.27)	322 (39.03)	24 (2.91)	41 (0.41)	3197 (32.17)	837 (8.42)	2324 (23.38)	3366 (33.87)	173 (1.74)
1996	52 (5.90)	229 (25.99)	42 (4.77)	209 (23.72)	325 (36.89)	24 (2.72)	58 (0.56)	3423 (33.34)	748 (7.29)	2397 (23.35)	3456 (33.66)	185 (1.80)
1997	69 (7.56)	222 (24.32)	46 (5.04)	207 (22.67)	334 (36.58)	35 (3.83)	65 (0.62)	3535 (33.85)	766 (7.34)	2396 (22.94)	3530 (33.80)	151 (1.45)
1998	57 (6.44)	193 (21.81)	54 (6.10)	219 (24.75)	334 (37.74)	28 (3.16)	57 (0.54)	3618 (34.30)	771 (7.31)	2382 (22.58)	3521 (33.38)	198 (1.88)
1999	51 (5.40)	255 (27.01)	42 (4.45)	213 (22.56)	337 (35.70)	46 (4.87)	64 (0.58)	3866 (35.02)	715 (6.48)	2510 (22.74)	3675 (33.29)	208 (1.88)
2000	80 (8.22)	214 (21.99)	43 (4.42)	223 (22.92)	376 (38.64)	37 (3.80)	78 (0.67)	4037 (34.81)	736 (6.35)	2688 (23.18)	3865 (33.32)	194 (1.67)
2001	70 (7.14)	226 (23.04)	45 (4.59)	211 (21.51)	391 (39.86)	38 (3.87)	105 (0.87)	4220 (35.09)	799 (6.64)	2731 (22.71)	3971 (33.02)	199 (1.65)
2002	64 (6.77)	258 (27.27)	33 (3.49)	189 (19.98)	353 (37.32)	49 (5.18)	86 (0.73)	4182 (35.34)	690 (5.83)	2697 (22.79)	3975 (33.59)	203 (1.72)
2003	64 (6.76)	200 (21.12)	42 (4.44)	183 (19.32)	414 (43.72)	44 (4.65)	77 (0.64)	4272 (35.73)	723 (6.05)	2661 (22.25)	4009 (33.53)	215 (1.80)
2004	56 (5.85)	239 (24.97)	32 (3.34)	206 (21.53)	384 (40.13)	40 (4.18)	105 (0.85)	4496 (36.30)	684 (5.52)	2790 (22.53)	4086 (32.99)	223 (1.80)
2005	82 (8.25)	212 (21.33)	23 (2.31)	234 (23.54)	387 (38.93)	56 (5.63)	123 (0.98)	4547 (36.30)	678 (5.41)	2769 (22.10)	4134 (33.00)	276 (2.20)
2006	94 (9.00)	245 (23.44)	20 (1.91)	222 (21.24)	416 (39.81)	48 (4.59)	120 (0.92)	4746 (36.45)	668 (5.13)	2937 (22.56)	4300 (33.03)	248 (1.90)
2007	100 (8.99)	257 (23.11)	25 (2.25)	249 (22.39)	426 (38.31)	55 (4.95)	114 (0.84)	4883 (35.82)	654 (4.80)	3162 (23.20)	4571 (33.53)	248 (1.82)
2008	79 (7.36)	264 (24.60)	28 (2.61)	245 (22.83)	408 (38.02)	49 (4.57)	137 (1.01)	5045 (37.01)	593 (4.35)	3189 (23.40)	4398 (32.27)	268 (1.97)
2009	107 (8.92)	240 (20.02)	30 (2.50)	274 (22.85)	485 (40.45)	63 (5.25)	127 (0.94)	5167 (38.21)	588 (4.35)	3042 (22.49)	4303 (31.82)	297 (2.20)
2010	119 (9.65)	258 (20.92)	25 (2.03)	296 (24.01)	476 (38.61)	59 (4.79)	166 (1.17)	5245 (36.96)	558 (3.93)	3351 (23.61)	4556 (32.10)	315 (2.22)
2011	156 (11.87)	262 (19.94)	18 (1.37)	290 (22.07)	530 (40.33)	58 (4.41)	180 (1.27)	5407 (38.10)	554 (3.90)	3218 (22.67)	4517 (31.83)	317 (2.23)
2012	190 (14.87)	252 (19.72)	30 (2.35)	261 (20.42)	482 (37.72)	63 (4.93)	211 (1.50)	5410 (38.50)	611 (4.35)	3141 (22.35)	4300 (30.60)	379 (2.70)
2013	210 (14.80)	291 (20.51)	29 (2.04)	309 (21.78)	524 (36.93)	56 (3.95)	214 (1.57)	5270 (38.74)	580 (4.26)	2917 (21.44)	4261 (31.32)	363 (2.67)
2014	208 (15.40)	281 (20.80)	20 (1.48)	306 (22.65)	490 (36.27)	46 (3.40)	241 (1.71)	5519 (39.19)	537 (3.81)	3044 (21.61)	4319 (30.67)	423 (3.00)
2015	233 (15.66)	314 (21.10)	24 (1.61)	285 (19.15)	573 (38.51)	59 (3.97)	263 (1.82)	5681 (39.30)	576 (3.99)	3098 (21.43)	4418 (30.57)	418 (2.89)
2016	249 (16.14)	326 (21.13)	39 (2.53)	325 (21.06)	553 (35.84)	51 (3.31)	254 (1.77)	5602 (39.00)	589 (4.10)	3100 (21.58)	4397 (30.61)	421 (2.93)
2017	234 (15.23)	315 (20.51)	17 (1.11)	343 (22.33)	570 (37.11)	57 (3.71)	279 (1.97)	5706 (40.21)	522 (3.68)	3065 (21.60)	4190 (29.53)	427 (3.01)
2018	269 (16.39)	351 (21.39)	15 (0.91)	369 (22.49)	584 (35.59)	53 (3.23)	301 (2.11)	5768 (40.35)	497 (3.48)	2965 (20.74)	4309 (30.14)	456 (3.19)
2019	260 (15.14)	342 (19.92)	27 (1.57)	403 (23.47)	622 (36.23)	63 (3.67)	300 (2.09)	5792 (40.27)	482 (3.35)	3036 (21.11)	4309 (29.96)	465 (3.23)
2020	267 (15.99)	343 (20.54)	33 (1.98)	383 (22.93)	604 (36.17)	40 (2.40)	311 (2.34)	5456 (41.03)	514 (3.86)	2596 (19.52)	3949 (29.69)	473 (3.56)
Total	3704 (10.61)	7802 (22.35)	1157 (3.31)	7756 (22.22)	13119 (37.58)	1369 (3.92)	4295 (1.13)	137848 (36.39)	20966 (5.54)	85371 (22.54)	121831 (32.16)	8473 (2.24)
**Males**												
1990	12 (3.08)	113 (28.97)	31 (7.95)	86 (22.05)	136 (34.87)	12 (3.08)	19 (0.42)	1120 (25.05)	461 (10.31)	1119 (25.03)	1696 (37.93)	56 (1.25)
1991	20 (5.24)	98 (25.65)	38 (9.95)	77 (20.16)	140 (36.65)	9 (2.36)	18 (0.37)	1311 (26.90)	448 (9.19)	1240 (25.45)	1791 (36.75)	65 (1.33)
1992	17 (4.12)	104 (25.18)	25 (6.05)	101 (24.46)	150 (36.32)	16 (3.87)	13 (0.27)	1285 (26.71)	394 (8.19)	1236 (25.69)	1821 (37.85)	62 (1.29)
1993	25 (5.41)	119 (25.76)	29 (6.28)	89 (19.26)	186 (40.26)	14 (3.03)	26 (0.52)	1281 (25.86)	401 (8.09)	1328 (26.81)	1853 (37.40)	65 (1.31)
1994	17 (4.15)	108 (26.34)	25 (6.10)	79 (19.27)	170 (41.46)	11 (2.68)	21 (0.40)	1391 (26.71)	429 (8.24)	1333 (25.60)	1971 (37.85)	62 (1.19)
1995	21 (4.86)	117 (27.08)	27 (6.25)	72 (16.67)	182 (42.13)	13 (3.01)	17 (0.31)	1504 (27.77)	424 (7.83)	1320 (24.37)	2066 (38.15)	85 (1.57)
1996	19 (4.08)	114 (24.46)	24 (5.15)	115 (24.68)	183 (39.27)	11 (2.36)	31 (0.55)	1629 (28.74)	362 (6.39)	1410 (24.88)	2146 (37.86)	90 (1.59)
1997	18 (4.11)	110 (25.11)	24 (5.48)	96 (21.92)	173 (39.50)	17 (3.88)	25 (0.43)	1676 (29.12)	389 (6.76)	1399 (24.31)	2199 (38.20)	68 (1.18)
1998	18 (3.93)	117 (25.55)	33 (7.21)	104 (22.71)	170 (37.12)	16 (3.49)	21 (0.37)	1629 (28.48)	382 (6.68)	1404 (24.55)	2203 (38.52)	80 (1.40)
1999	10 (2.23)	124 (27.62)	23 (5.12)	91 (20.27)	179 (39.87)	22 (4.90)	22 (0.37)	1795 (30.22)	362 (6.10)	1414 (23.81)	2259 (38.04)	87 (1.46)
2000	38 (7.47)	108 (21.22)	23 (4.52)	107 (21.02)	214 (42.04)	19 (3.73)	35 (0.54)	1946 (30.22)	371 (5.76)	1565 (24.31)	2433 (37.79)	89 (1.38)

2001	32 (6.56)	116 (23.77)	18 (3.69)	93 (19.06)	214 (43.85)	15 (3.07)	40 (0.61)	1950 (29.78)	374 (5.71)	1650 (25.20)	2439 (37.25)	95 (1.45)
2002	29 (6.33)	116 (25.33)	18 (3.93)	86 (18.78)	188 (41.05)	21 (4.59)	36 (0.55)	2003 (30.59)	319 (4.87)	1624 (24.80)	2472 (37.75)	94 (1.44)
2003	23 (5.10)	99 (21.95)	22 (4.88)	69 (15.30)	219 (48.56)	19 (4.21)	27 (0.41)	2016 (30.80)	363 (5.55)	1578 (24.11)	2471 (37.75)	90 (1.38)
2004	24 (4.99)	120 (24.95)	14 (2.91)	96 (19.96)	211 (43.87)	16 (3.33)	39 (0.57)	2124 (31.01)	332 (4.85)	1648 (24.06)	2597 (37.91)	110 (1.61)
2005	33 (6.53)	104 (20.59)	13 (2.57)	109 (21.58)	219 (43.37)	27 (5.35)	50 (0.73)	2148 (31.25)	332 (4.83)	1640 (23.86)	2583 (37.58)	121 (1.76)
2006	34 (6.61)	119 (23.15)	11 (2.14)	111 (21.60)	220 (42.80)	19 (3.70)	61 (0.86)	2234 (31.42)	348 (4.90)	1692 (23.80)	2668 (37.53)	106 (1.49)
2007	41 (7.78)	124 (23.53)	8 (1.52)	108 (20.49)	218 (41.37)	28 (5.31)	48 (0.64)	2284 (30.40)	306 (4.07)	1885 (25.09)	2883 (38.37)	107 (1.42)
2008	35 (6.28)	136 (24.42)	15 (2.69)	119 (21.36)	230 (41.29)	22 (3.95)	65 (0.87)	2349 (31.38)	282 (3.77)	1923 (25.69)	2756 (36.82)	110 (1.47)
2009	43 (6.95)	137 (22.13)	19 (3.07)	123 (19.87)	278 (44.91)	19 (3.07)	53 (0.71)	2408 (32.32)	279 (3.74)	1855 (24.90)	2727 (36.60)	129 (1.73)
2010	44 (6.92)	144 (22.64)	14 (2.20)	137 (21.54)	273 (42.92)	24 (3.77)	67 (0.85)	2467 (31.27)	254 (3.22)	2007 (25.44)	2975 (37.71)	119 (1.51)
2011	64 (9.41)	144 (21.18)	4 (0.59)	129 (18.97)	312 (45.88)	27 (3.97)	77 (0.99)	2554 (32.97)	242 (3.12)	1919 (24.77)	2832 (36.56)	123 (1.59)
2012	73 (11.76)	125 (20.13)	16 (2.58)	117 (18.84)	263 (42.35)	27 (4.35)	84 (1.09)	2613 (33.82)	279 (3.61)	1893 (24.50)	2702 (34.97)	155 (2.01)
2013	89 (12.55)	163 (22.99)	14 (1.97)	159 (22.43)	261 (36.81)	23 (3.24)	106 (1.44)	2452 (33.24)	288 (3.90)	1706 (23.13)	2675 (36.26)	150 (2.03)
2014	91 (13.09)	146 (21.01)	9 (1.29)	155 (22.30)	279 (40.14)	15 (2.16)	117 (1.52)	2573 (33.51)	240 (3.13)	1812 (23.60)	2763 (35.98)	174 (2.27)
2015	100 (13.68)	163 (22.30)	13 (1.78)	131 (17.92)	302 (41.31)	22 (3.01)	123 (1.55)	2725 (34.24)	275 (3.46)	1850 (23.24)	2804 (35.23)	182 (2.29)
2016	101 (13.27)	164 (21.55)	17 (2.23)	157 (20.63)	301 (39.55)	21 (2.76)	124 (1.58)	2628 (33.41)	267 (3.39)	1885 (23.96)	2787 (35.43)	175 (2.22)
2017	103 (13.81)	162 (21.72)	8 (1.07)	153 (20.51)	301 (40.35)	19 (2.55)	136 (1.78)	2689 (35.17)	241 (3.15)	1822 (23.83)	2585 (33.81)	172 (2.25)
2018	105 (13.00)	178 (22.03)	11 (1.36)	167 (20.67)	332 (41.09)	15 (1.86)	160 (2.05)	2705 (34.74)	222 (2.85)	1769 (22.72)	2748 (35.29)	182 (2.34)
2019	111 (13.20)	152 (18.07)	17 (2.02)	200 (23.78)	336 (39.95)	25 (2.97)	142 (1.80)	2752 (34.95)	219 (2.78)	1841 (23.38)	2749 (34.91)	172 (2.18)
2020	100 (11.86)	182 (21.59)	21 (2.49)	170 (20.17)	345 (40.93)	25 (2.97)	143 (2.00)	2621 (36.66)	231 (3.23)	1535 (21.47)	2455 (34.34)	165 (2.31)
Total	1490 (8.52)	4026 (23.03)	584 (3.34)	3606 (20.63)	7185 (41.10)	589 (3.37)	1946 (0.94)	64862 (31.35)	10116 (4.89)	50302 (24.32)	76109 (36.79)	3540 (1.71)
**Females**												
1990	24 (6.08)	91 (23.04)	51 (12.91)	95 (24.05)	123 (31.14)	11 (2.78)	17 (0.45)	1258 (33.04)	449 (11.79)	912 (23.96)	1079 (28.34)	92 (2.42)
1991	27 (6.72)	96 (23.88)	33 (8.21)	111 (27.61)	122 (30.35)	13 (3.23)	26 (0.63)	1430 (34.47)	423 (10.20)	1021 (24.61)	1174 (28.30)	74 (1.78)
1992	38 (9.22)	104 (25.24)	38 (9.22)	107 (25.97)	114 (27.67)	11 (2.67)	20 (0.47)	1493 (34.99)	433 (10.15)	984 (23.06)	1251 (29.32)	86 (2.02)
1993	25 (6.70)	81 (21.72)	23 (6.17)	87 (23.32)	143 (38.34)	14 (3.75)	27 (0.63)	1581 (36.94)	430 (10.05)	975 (22.78)	1194 (27.90)	73 (1.71)
1994	26 (6.45)	86 (21.34)	28 (6.95)	111 (27.54)	135 (33.50)	17 (4.22)	31 (0.69)	1608 (35.77)	428 (9.52)	1017 (22.63)	1316 (29.28)	95 (2.11)
1995	32 (8.14)	96 (24.43)	27 (6.87)	87 (22.14)	140 (35.62)	11 (2.80)	24 (0.53)	1693 (37.44)	413 (9.13)	1004 (22.20)	1300 (28.75)	88 (1.95)
1996	33 (7.95)	115 (27.71)	18 (4.34)	94 (22.65)	142 (34.22)	13 (3.13)	27 (0.59)	1794 (39.01)	386 (8.39)	987 (21.46)	1310 (28.48)	95 (2.07)
1997	51 (10.74)	112 (23.58)	22 (4.63)	111 (23.37)	161 (33.89)	18 (3.79)	40 (0.85)	1859 (39.66)	377 (8.04)	997 (21.27)	1331 (28.40)	83 (1.77)
1998	39 (9.13)	76 (17.80)	21 (4.92)	115 (26.93)	164 (38.41)	12 (2.81)	36 (0.75)	1989 (41.20)	389 (8.06)	978 (20.26)	1318 (27.30)	118 (2.44)
1999	41 (8.28)	131 (26.46)	19 (3.84)	122 (24.65)	158 (31.92)	24 (4.85)	42 (0.82)	2071 (40.62)	353 (6.92)	1096 (21.49)	1416 (27.77)	121 (2.37)
2000	42 (9.05)	106 (22.84)	20 (4.31)	116 (25.00)	162 (34.91)	18 (3.88)	43 (0.83)	2091 (40.53)	365 (7.08)	1123 (21.77)	1432 (27.76)	105 (2.04)
2001	38 (7.71)	110 (22.31)	27 (5.48)	118 (23.94)	177 (35.90)	23 (4.67)	65 (1.19)	2270 (41.45)	425 (7.76)	1081 (19.74)	1532 (27.97)	104 (1.90)
2002	35 (7.17)	142 (29.10)	15 (3.07)	103 (21.11)	165 (33.81)	28 (5.74)	50 (0.95)	2179 (41.23)	371 (7.02)	1073 (20.30)	1503 (28.44)	109 (2.06)
2003	41 (8.27)	101 (20.36)	20 (4.03)	114 (22.98)	195 (39.31)	25 (5.04)	50 (0.92)	2256 (41.69)	360 (6.65)	1083 (20.01)	1538 (28.42)	125 (2.31)
2004	32 (6.72)	119 (25.00)	18 (3.78)	110 (23.11)	173 (36.34)	24 (5.04)	66 (1.19)	2372 (42.86)	352 (6.36)	1142 (20.64)	1489 (26.91)	113 (2.04)
2005	49 (10.02)	108 (22.09)	10 (2.04)	125 (25.56)	168 (34.36)	29 (5.93)	73 (1.29)	2399 (42.44)	346 (6.12)	1129 (19.97)	1551 (27.44)	155 (2.74)
2006	60 (11.30)	126 (23.73)	9 (1.69)	111 (20.90)	196 (36.91)	29 (5.46)	59 (1.00)	2512 (42.51)	320 (5.42)	1244 (21.05)	1632 (27.62)	142 (2.40)
2007	59 (10.09)	133 (22.74)	17 (2.91)	141 (24.10)	208 (35.56)	27 (4.62)	66 (1.08)	2599 (42.47)	348 (5.69)	1277 (20.87)	1688 (27.59)	141 (2.30)
2008	44 (8.53)	128 (24.81)	13 (2.52)	126 (24.42)	178 (34.50)	27 (5.23)	72 (1.17)	2696 (43.87)	311 (5.06)	1266 (20.60)	1642 (26.72)	158 (2.57)
2009	64 (11.03)	103 (17.76)	11 (1.90)	151 (26.03)	207 (35.69)	44 (7.59)	74 (1.22)	2759 (45.43)	309 (5.09)	1187 (19.55)	1576 (25.95)	168 (2.77)
2010	75 (12.56)	114 (19.10)	11 (1.84)	159 (26.63)	203 (34.00)	35 (5.86)	99 (1.57)	2778 (44.08)	304 (4.82)	1344 (21.33)	1581 (25.09)	196 (3.11)
2011	92 (14.51)	118 (18.61)	14 (2.21)	161 (25.39)	218 (34.38)	31 (4.89)	103 (1.60)	2853 (44.26)	312 (4.84)	1299 (20.15)	1685 (26.14)	194 (3.01)
2012	117 (17.81)	127 (19.33)	14 (2.13)	144 (21.92)	219 (33.33)	36 (5.48)	127 (2.01)	2797 (44.21)	332 (5.25)	1248 (19.73)	1598 (25.26)	224 (3.54)
2013	121 (17.04)	128 (18.03)	15 (2.11)	150 (21.13)	263 (37.04)	33 (4.65)	108 (1.73)	2818 (45.25)	292 (4.69)	1211 (19.44)	1586 (25.47)	213 (3.42)
2014	117 (17.84)	135 (20.58)	11 (1.68)	151 (23.02)	211 (32.16)	31 (4.73)	124 (1.94)	2946 (46.00)	297 (4.64)	1232 (19.24)	1556 (24.30)	249 (3.89)
2015	133 (17.57)	151 (19.95)	11 (1.45)	154 (20.34)	271 (35.80)	37 (4.89)	140 (2.16)	2956 (45.51)	301 (4.63)	1248 (19.21)	1614 (24.85)	236 (3.63)

2016	148 (18.93)	162 (20.72)	22 (2.81)	168 (21.48)	252 (32.23)	30 (3.84)	130 (2.00)	2974 (45.77)	322 (4.96)	1215 (18.70)	1610 (24.78)	246 (3.79)
2017	131 (16.58)	153 (19.37)	9 (1.14)	190 (24.05)	269 (34.05)	38 (4.81)	143 (2.19)	3017 (46.10)	281 (4.29)	1243 (18.99)	1605 (24.53)	255 (3.90)
2018	164 (19.69)	173 (20.77)	4 (0.48)	202 (24.25)	252 (30.25)	38 (4.56)	141 (2.17)	3063 (47.05)	275 (4.22)	1196 (18.37)	1561 (23.98)	274 (4.21)
2019	149 (17.01)	190 (21.69)	10 (1.14)	203 (23.17)	286 (32.65)	38 (4.34)	158 (2.43)	3040 (46.70)	263 (4.04)	1195 (18.36)	1560 (23.97)	293 (4.50)
2020	167 (20.19)	161 (19.47)	12 (1.45)	213 (25.76)	259 (31.32)	15 (1.81)	168 (2.73)	2835 (46.11)	283 (4.60)	1061 (17.25)	1494 (24.30)	308 (5.01)
Total	2214 (12.70)	3776 (21.67)	573 (3.29)	4150 (23.81)	5934 (34.05)	780 (4.48)	2349 (1.37)	72986 (42.46)	10850 (6.31)	35068 (20.40)	45722 (26.60)	4933 (2.87)

Note: Data present number of incident cases, with crude percentage contribution of each anatomical site in parentheses relative to total number of cases within each age stratum.

Table S3: Temporal trends in tumour incidence rates by age, anatomical site, and calendar period of diagnosis for all histological subtypes combined.

**Age, years**	***n***	**Trend 1**		**Trend 2**		**Trend 3**		**Trend 4**	
		**Period**	**APC (95% CI)**	**Period**	**APC (95% CI)**	**Period**	**APC (95% CI)**	**Period**	**APC (95% CI)**
**Appendix**									
20-29	1,290	1990-2004	0.8 (-10.9, 4.1)	2004-2020	9.5 (6.6, 21.8)				
30-39	1,123	1990-2007	2.9 (-0.1, 5.0)	2007-2013	17.5 (7.6, 36.2)	2013-2020	1.1 (-11.8, 7.2)		
40-49	1,291	1990-2020	6.6 (5.5, 7.8)						
50-59	1,309	1990-2020	6.9 (6.0, 7.8)						
60-69	1,351	1990-2020	4.6 (3.8, 5.5)						
70-79	1,066	1990-2020	4.8 (3.7, 5.9)						
80-89	508	1990-2020	4.6 (3.0, 6.3)						
90+	61	1990-2020							
**Proximal colon**									
20-29	495	1990-1997	-5.4 (-26.6, 3.0)	1997-2011	9.4 (6.1, 36.2)	2011-2020	-4.3 (-15.9, 1.7)		
30-39	1,901	1990-2010	1.9 (-5.0, 3.1)	2010-2020	6.5 (3.3, 25.1)				
40-49	5,406	1990-2011	-0.9 (-4.2, -0.1)	2011-2020	1.6 (-0.4, 10.2)				
50-59	15,251	1990-2020	-1.5 (-1.8, -1.2)						
60-69	33,326	1990-2001	2.4 (1.6, 3.4)	2001-2020	-2.4 (-2.8, -2.0)				
70-79	48,690	1990-1999	3.6 (2.6, 6.3)	1999-2007	1.3 (-0.6, 3.5)	2007-2016	-0.9 (-2.7, 0.3)	2016-2020	-7.3 (-10.6, -5.4)
80-89	35,239	1990-1992	7.2 (1.9, 11.2)	1992-2011	1.8 (-2.4, 2.1)	2011-2020	-0.9 (-2.3, 0.9)		
90+	5,342	1990-2020	0.9 (0.4, 1.4)						
**Distal colon**									
20-29	389	1990-2010	8.7 (6.1, 21.5)	2010-2020	-2.9 (-25.9, 3.9)				
30-39	1,681	1990-2008	1.2 (-2.0, 2.5)	2008-2020	7.0 (4.5, 15.4)				
40-49	5,686	1990-2003	-1.2 (-4.4, 0.0)	2003-2020	2.0 (1.2, 4.1)				

50-59	15,110	1990-2004	-2.4 (-3.6, -1.8)	2004-2010	2.4 (0.1, 7.8)	2010-2015	-5.7 (-10.8, -3.1)	2015-2020	2.8 (0.0, 10.0)
60-69	25,737	1990-2008	-0.5 (-1.0, 0.2)	2008-2020	-4.2 (-5.5, -3.2)				
70-79	27,668	1990-2008	0.4 (0.0, 0.9)	2008-2017	-3.2 (-4.7, -1.8)	2017-2020	-13.4 (-19.5, -9.2)		
80-89	14,884	1990-2011	0.0 (-0.4, 0.4)	2011-2020	-3.1 (-5.2, -2.0)				
90+	1,972	1990-2020	-1.3 (-1.8, -0.7)						
**Rectum**									
20-29	622	1990-2013	7.2 (5.7, 12.4)	2013-2020	-4.3 (-25.6, 4.1)				
30-39	3,027	1990-2015	3.0 (-0.1, 3.9)	2015-2020	8.6 (3.4, 21.9)				
40-49	9,470	1990-2020	0.9 (0.6, 1.3)						
50-59	24,329	1990-2003	-0.9 (-1.9, -0.5)	2003-2007	2.9 (0.3, 5.6)	2007-2013	-4.3 (-7.7, -2.9)	2013-2020	0.8 (-0.4, 3.0)
60-69	37,618	1990-1994	4.1 (1.3, 9.5)	1994-2007	-0.4 (-1.4, 0.2)	2007-2020	-3.5 (-4.2, -3.0)		
70-79	37,284	1990-2000	1.3 (-2.2, 5.3)	2000-2011	-1.3 (-4.6, 4.7)	2011-2016	-3.8 (-8.9, 1.3)	2016-2020	-8.3 (-12.7, -4.8)
80-89	19,564	1990-2007	-0.1 (-0.6, 0.8)	2007-2020	-2.8 (-4.1, -2.0)				
90+	3,036	1990-2020	-1.1 (-1.6, -0.6)						
**Anus**									
20-29	36	1990-2020							
30-39	258	1990-2020	-0.1 (-1.7, 1.5)						
40-49	1,075	1990-2009	4.9 (3.7, 6.8)	2009-2020	-2.0 (-6.6, 0.4)				
50-59	2,179	1990-2020	3.2 (2.4, 4.1)						
60-69	2,604	1990-2020	2.6 (2.2, 3.0)						
70-79	2,205	1990-1995	7.5 (0.5, 22.7)	1995-2003	-4.5 (-14.9, -0.4)	2003-2020	3.1 (1.7, 5.5)		
80-89	1,217	1990-1992	-19.3 (-29.1, 0.5)	1992-2020	0.6 (-1.8, 4.8)				
90+	268	1990-2020	-0.6 (-2.5, 1.3)						

APC, annual percentage change; CI, confidence interval; *n*, number of cases.

Table S4: Temporal trends in cancer incidence rates by age, anatomical site, sex, and calendar period of diagnosis after excluding neuroendocrine neoplasms.

**Age, years**	***n***	**Trend 1**		**Trend 2**		**Trend 3**		**Trend 4**	
		**Period**	**APC (95% CI)**	**Period**	**APC (95% CI)**	**Period**	**APC (95% CI)**	**Period**	**APC (95% CI)**
**Male appendiceal cancer**									
20-29	35	2000-2017	-0.68 (-8.59, 7.89)						
30-39	111	2000-2020	5.76 (-3.76, 15.751)						

40-49	298	1990-2020	7.16 (4.61, 9.82)						
50-59	375	1993-2020	7.47 (5.25, 9.80)						
60-69	434	1990-2020	5.05 (3.16, 7.06)						
70-79	363	1990-2020	4.07 (2.24, 5.97)						
80-89	154	1990-2020	4.03 (2.05, 6.09)						
90+	17	1990-2020							
**Female appendiceal cancer**									
20-29	38	1990-2020							
30-39	112	1998-2020	7.06 (-0.21, 14.81)						
40-49	313	1991-2020	5.12 (3.09, 7.31)						
50-59	418	1990-2020	5.18 (3.44, 7.03)						
60-69	476	1991-2020	3.76 (2.56, 5.02)						
70-79	425	1990-2020	4.87 (3.86, 5.90)						
80-89	214	1990-2020	4.30 (1.32, 7.52)						
90+	33	1990-2020							
**Male proximal colon cancer**									
20-29	236	1990-2020	3.25 (0.65, 5.92)						
30-39	927	1990-2020	2.50 (1.76, 3.28)						
40-49	2,787	1990-2003	-2.11 (-6.22, -0.75)	2003-2020	1.06 (0.17, 4.20)				
50-59	7,836	1990-2020	-1.37 (-1.71, -1.01)						
60-69	17,116	1990-2000	2.34 (1.26, 3.98)	2000-2020	-2.10 (-2.57, -1.71)				
70-79	23,220	1990-2002	3.21 (2.52, 4.24)	2002-2016	-0.80 (-1.41, -0.11)	2016-2020	-7.28 (-12.39, -4.46)		
80-89	14,284	1990-2011	1.64 (1.26, 2.18)	2011-2020	-1.62 (-3.78, -0.37)				
90+	1,736	1990-2020	0.64 (-0.28, 1.61)						
**Female proximal colon cancer**									
20-29	246	1990-2014	7.77 (5.08, 53.11)	2014-2020	-12.77 (-55.92, 4.17)				
30-39	922	1990-2009	1.51 (-9.76, 3.56)	2009-2020	8.06 (3.77, 34.05)				
40-49	2,527	1990-2020	-0.58 (-1.25, 0.11)						
50-59	7,147	1990-2020	-1.80 (-2.20, -1.39)						
60-69	15,820	1990-2001	2.81 (1.70, 4.41)	2001-2020	-2.68 (-3.26, -2.20)				
70-79	25,029	1990-1996	4.45 (3.02, 8.07)	1996-2007	1.92 (0.32, 2.70)	2007-2016	-0.73 (-2.63, 0.31)	2016-2020	-8.10 (-11.70, -5.98)
80-89	20,731	1990-2010	2.19 (1.85, 2.65)	2010-2020	-0.40 (-1.82, 0.46)				

90+	3,575	1990-2020	0.96 (0.50, 1.44)						
**Male distal colon cancer**									
20-29	181	1994-1999	37.38 (10.17, 138.95)	1999-2020	2.45 (-5.24, 5.27)				
30-39	724	1990-2003	0.54 (-16.11, 4.13)	2003-2020	6.05 (3.42, 24.58)				
40-49	2,652	1990-2003	-1.64 (-9.80, 0.26)	2003-2020	2.26 (0.96, 8.72)				
50-59	8,462	1990-2020	-1.11 (-1.60, -0.61)						
60-69	16,130	1990-2007	-0.12 (-0.77, 0.77)	2007-2020	-4.01 (-5.37, -3.07)				
70-79	16,957	1990-2008	0.46 (-0.04, 1.31)	2008-2017	-3.42 (-5.20, -1.48)	2017-2020	-14.72 (-22.50, -9.56)		
80-89	7,839	1990-2010	-0.03 (-0.68, 3.53)	2010-2020	-3.20 (-9.60, -1.31)				
90+	747	1990-2020	-1.39 (-2.38, -0.38)						
**Female distal colon cancer**									
20-29	196	1990-2020	4.94 (2.44, 7.55)						
30-39	933	1990-2008	0.54 (-7.13, 2.36)	2008-2020	6.81 (3.41, 24.52)				
40-49	2,981	1990-2003	-0.91 (-6.94, 0.44)	2003-2020	1.71 (0.83, 6.90)				
50-59	6,557	1990-2020	-1.50 (-2.03, -0.94)						
60-69	9,513	1990-2008	-0.91 (-1.65, 1.05)	2008-2020	-4.01 (-7.24, -2.66)				
70-79	10,636	1990-2008	-0.12 (-0.93, 2.64)	2008-2017	-3.24 (-4.81, 0.40)	2017-2020	-11.17 (-18.85, -5.06)		
80-89	7,019	1990-2016	-0.51 (-0.80, -0.13)	2016-2020	-8.07 (-15.23, -4.12)				
90+	1,224	1990-2020	-1.38 (-2.20, -0.55)						
**Male rectal cancer**									
20-29	275	1990-1997	26.50 (9.82, 89.02)	1997-2020	2.63 (-3.71, 5.08)				
30-39	1,345	1990-2020	3.75 (2.90, 4.63)						
40-49	4,876	1990-2020	0.46 (0.06, 0.88)						
50-59	14,758	1990-2010	-0.47 (-0.80, -0.05)	2010-2013	-6.71 (-8.70, -2.40)	2013-2020	0.55 (-0.99, 5.28)		
60-69	24,631	1990-1994	5.53 (2.24, 12.15)	1994-2007	-0.63 (-1.57, 0.10)	2007-2020	-3.72 (-4.67, -3.10)		
70-79	23,495	1990-2000	1.36 (-2.94, 5.96)	2000-2011	-1.48 (-6.56, 5.79)	2011-2018	-4.85 (-6.50, 2.86)	2018-2020	-12.50 (-17.77, -5.30)
80-89	10,454	1990-2011	-0.41 (-0.97, 0.98)	2011-2020	-3.92 (-9.53, -1.97)				
90+	1,208	1990-2020	-1.07 (-1.93, -0.18)						
**Female rectal cancer**									
20-29	244	1990-1995	-12.86 (-43.00, 4.75)	1995-2013	8.99 (5.60, 46.65)	2013-2020	-11.91 (-32.24, 0.71)		
30-39	1,273	1990-2020	2.32 (1.47, 3.20)						
40-49	3,810	1990-2020	0.56 (0.13, 1.00)						

50-59	8,503	1990-2020	-0.98 (-1.34, -0.61)						
60-69	11,990	1990-2007	-0.27 (-0.79, 0.38)	2007-2020	-3.73 (-4.74, -2.98)				
70-79	13,175	1990-2001	0.73 (-0.10, 2.51)	2001-2015	-2.07 (-2.99, -1.34)	2015-2020	-8.69 (-12.75, -6.33)		
80-89	8,960	1990-2001	0.61 (-0.40, 2.45)	2001-2020	-2.68 (-3.35, -2.23)				
90+	1,812	1990-2020	-1.32 (-1.92, -0.68)						
**Male anal cancer**									
20-29	17	1990-2020							
30-39	125	1990-2020	-1.67 (-4.53, 1.42)						
40-49	436	1990-2020	1.71 (0.37, 3.08)						
50-59	896	1990-2020	2.21 (1.06, 3.42)						
60-69	1,138	1990-2020	1.19 (0.47, 1.94)						
70-79	935	1990-2020	0.52 (-0.49, 1.56)						
80-89	462	1990-2020	0.21 (-0.66, 1.14)						
90+	66	1990-2020							
**Female anal cancer**									
20-29	19	1990-2020							
30-39	93	2001-2020	-1.49 (-5.09, 2.25)						
40-49	619	1990-2009	5.95 (4.20, 8.97)	2009-2020	-2.85 (-10.28, 0.66)				
50-59	1,257	1990-2014	5.12 (3.79, 16.50)	2014-2020	-3.92 (-26.09, 3.91)				
60-69	1,436	1990-1992	-14.63 (-21.77, 3.10)	1992-2020	4.12 (3.57, 5.32)				
70-79	1,251	1990-1994	10.92 (2.95, 26.64)	1994-2008	-3.76 (-7.43, -2.32)	2008-2011	18.19 (-1.21, 24.67)	2011-2020	3.19 (-4.10, 5.87)
80-89	736	1990-1992	-25.27 (-37.83, 1.45)	1992-2020	0.81 (-20.21, 10.88)				
90+	201	1990-2020							

APC, annual percentage change; CI, confidence interval; *n*, number of cases.

Table S5: Temporal trends in tumour incidence rates by age, anatomical site, sex, and calendar period of diagnosis for all histological subtypes combined.

Age, years	*n*	Trend 1		Trend 2		Trend 3		Trend 4	
		Period	APC (95% CI)	Period	APC (95% CI)	Period	APC (95% CI)	Period	APC (95% CI)
**Male appendiceal tumour**									
20-29	479	1990-1997	-6.78 (-38.27, 5.55)	1997-2020	7.88 (5.30, 22.86)				
30-39	453	1990-2020	7.74 (5.60, 9.87)						
40-49	558	1990-2020	7.80 (5.79, 9.83)						

50-59	592	1990-2020	7.47 (5.70, 9.23)						
60-69	627	1990-2003	0.90 (-16.95, 4.60)	2003-2020	7.73 (5.19, 25.26)				
70-79	495	1990-2020	4.44 (2.97, 6.00)						
80-89	210	1990-1998	-6.36 (-36.79, 4.13)	1998-2020	8.25 (5.52, 21.91)				
90+	22	1990-2020							
**Female appendiceal tumour**									
20-29	811	1990-2004	0.04 (-15.61, 4.37)	2004-2020	10.35 (6.71, 29.00)				
30-39	670	1990-2002	0.77 (-11.12, 4.35)	2002-2020	8.53 (6.48, 15.68)				
40-49	733	1990-2020	6.10 (4.69, 7.62)						
50-59	717	1990-2020	6.09 (4.96, 7.29)						
60-69	724	1990-1992	54.81 (5.00, 116.58)	1992-2020	3.99 (-8.78, 5.38)				
70-79	571	1990-2020	5.04 (3.70, 6.44)						
80-89	298	1990-2020	4.59 (2.18, 7.19)						
90+	39	1990-2020							
**Male proximal colon tumour**									
20-29	243	1990-2020	3.51 (0.93, 6.22)						
30-39	944	1990-2020	2.63 (1.85, 3.43)						
40-49	2,839	1990-2003	-1.87 (-7.44, -0.51)	2003-2020	0.98 (0.09, 5.60)				
50-59	7,967	1990-2020	-1.30 (-1.64, -0.94)						
60-69	17,323	1990-2000	2.30 (1.22, 3.95)	2000-2020	-2.02 (-2.50, -1.64)				
70-79	23,442	1990-2002	3.23 (2.53, 4.26)	2002-2016	-0.74 (-1.35, -0.05)	2016-2020	-7.12 (-12.33, -4.21)		
80-89	14,384	1990-2011	1.65 (1.28, 2.18)	2011-2020	-1.58 (-3.77, -0.35)				
90+	1,746	1990-2020	0.65 (-0.24, 1.57)						
**Female proximal colon tumour**									
20-29	252	1990-2014	7.62 (4.76, 65.88)	2014-2020	-12.64 (-56.75, 4.38)				
30-39	957	1990-2012	2.17 (-7.68, 3.76)	2012-2020	10.30 (3.87, 42.67)				
40-49	2,567	1990-2020	-0.49 (-1.15, 0.19)						
50-59	7,284	1990-2020	-1.72 (-2.09, -1.35)						
60-69	16,003	1990-2001	2.83 (1.71, 4.44)	2001-2020	-2.62 (-3.22, -2.15)				
70-79	25,248	1990-1996	4.48 (2.84, 8.71)	1996-2007	1.92 (-0.30, 2.72)	2007-2016	-0.69 (-2.63, 0.62)	2016-2020	-7.98 (-11.86, -5.70)
80-89	20,855	1990-2010	2.19 (1.86, 2.62)	2010-2020	-0.37 (-1.71, 0.47)				
90+	3596	1990-2020	0.95 (0.51, 1.40)						

**Male distal colon tumour**									
20-29	185	1994-1999	37.37 (12.03, 131.92)	1999-2020	2.65 (-3.11, 5.32)				
30-39	734	1990-2003	0.59 (-15.99, 4.25)	2003-2020	6.20 (3.45, 23.93)				
40-49	2,680	1990-2003	-1.60 (-10.23, 0.37)	2003-2020	2.31 (0.98, 9.63)				
50-59	8,514	1990-2020	-1.08 (-1.58, -0.57)						
60-69	16,193	1990-2007	-0.11 (-0.77, 0.82)	2007-2020	-3.95 (-5.40, -2.97)				
70-79	16,997	1990-2008	0.47 (-0.03, 1.25)	2008-2017	-3.39 (-5.21, -1.56)	2017-2020	-14.79 (-22.47, -9.70)		
80-89	7,850	1990-2010	-0.02 (-0.68, 3.11)	2010-2020	-3.21 (-9.57, -1.34)				
90+	748	1990-2020	-1.38 (-2.38, -0.38)						
**Female distal colon tumour**									
20-29	197	1990-2020	5.01 (2.57, 7.55)						
30-39	947	1990-2008	0.56 (-10.19, 2.92)	2008-2020	6.96 (2.71, 29.27)				
40-49	3,006	1990-2003	-0.93 (-7.95, 0.53)	2003-2020	1.78 (0.80, 7.81)				
50-59	6,596	1990-2020	-1.47 (-2.00, -0.91)						
60-69	9,543	1990-2008	-0.90 (-1.65, 1.30)	2008-2020	-3.96 (-7.51, -2.57)				
70-79	10,671	1990-2008	-0.10 (-1.25, 3.27)	2008-2017	-3.19 (-4.77, 0.72)	2017-2020	-10.98 (-18.90, -4.80)		
80-89	7,034	1990-2016	-0.50 (-0.79, -0.12)	2016-2020	-7.92 (-14.87, -3.98)				
90+	1,224	1990-2020	-1.38 (-2.20, -0.55)						
**Male rectal tumour**									
20-29	332	1990-1997	25.07 (9.72, 92.29)	1997-2020	3.95 (-3.37, 6.30)				
30-39	1,570	1990-2020	4.22 (3.60, 4.88)						
40-49	5,283	1990-2020	0.90 (0.47, 1.34)						
50-59	15,327	1990-2010	-0.24 (-1.30, 0.37)	2010-2013	-6.32 (-8.72, 1.70)	2013-2020	0.74 (-1.62, 6.80)		
60-69	25,165	1990-1994	5.43 (2.30, 11.81)	1994-2007	-0.53 (-1.43, 0.19)	2007-2020	-3.49 (-4.40, -2.90)		
70-79	23,867	1990-2000	1.38 (-2.88, 6.02)	2000-2011	-1.43 (-6.46, 5.97)	2011-2018	-4.53 (-6.21, 3.04)	2018-2020	-12.15 (-17.33, -5.00)
80-89	10,539	1990-2011	-0.37 (-0.93, 1.02)	2011-2020	-3.87 (-9.26, -1.92)				
90+	1,211	1990-2020	-1.07 (-1.94, -0.18)						
**Female rectal tumour**									
20-29	290	1990-2018	5.77 (4.16, 9.36)	2018-2020	-46.85 (-65.20, 1.45)				
30-39	1,457	1990-2020	2.77 (1.87, 3.69)						
40-49	4,187	1990-2020	0.97 (0.55, 1.41)						
50-59	9,002	1990-2020	-0.70 (-1.07, -0.33)						

60-69	12,453	1990-2007	-0.13 (-0.64, 0.53)	2007-2020	-3.32 (-4.33, -2.58)				
70-79	13,417	1990-2001	0.68 (-0.14, 2.80)	2001-2014	-1.74 (-2.96, -0.94)	2014-2020	-7.26 (-10.66, -5.49)		
80-89	9,025	1990-2001	0.48 (-2.62, 3.93)	2001-2018	-2.40 (-2.96, 3.58)	2018-2020	-9.15 (-14.92, -2.43)		
90+	1,825	1990-2020	-1.32 (-1.91, -0.68)						
**Male anal tumour**									
20-29	17	1990-2020							
30-39	128	1990-2020	-1.28 (-4.23, 1.88)						
40-49	444	1990-2020	1.86 (0.62, 3.14)						
50-59	908	1990-2020	2.23 (0.97, 3.56)						
60-69	1,154	1990-2020	1.26 (0.57, 1.97)						
70-79	942	1990-2020	0.55 (-0.45, 1.58)						
80-89	470	1990-2020	0.20 (-0.63, 1.07)						
90+	66	1990-2020							
**Female anal tumour**									
20-29	19	1990-2020							
30-39	96	2001-2020	-1.74 (-5.48, 2.07)						
40-49	631	1990-2009	6.03 (4.23, 9.33)	2009-2020	-2.83 (-10.65, 0.76)				
50-59	1,271	1990-2014	5.16 (3.73, 20.65)	2014-2020	-3.75 (-27.10, 4.11)				
60-69	1,450	1990-1992	-14.77 (-21.96, 3.14)	1992-2020	4.19 (3.65, 5.35)				
70-79	1,263	1990-1994	10.83 (3.51, 25.07)	1994-2008	-3.60 (-6.39, -2.32)	2008-2011	17.57 (-0.89, 23.50)	2011-2020	3.12 (-3.21, 5.49)
80-89	747	1990-2020	0.33 (-0.77, 1.49)						
90+	202	1990-2020	-0.80 (-3.24, 1.73)						

APC, annual percentage change; CI, confidence interval; *n*, number of cases.

Table S6: Temporal trends in appendiceal tumour incidence rates by age, histology, and calendar period of diagnosis.

**Age, years**	***n***	**Trend 1**		**Trend 2**		**Trend 3**	
		**Period**	**APC (95% CI)**	**Period**	**APC (95% CI)**	**Period**	**APC (95% CI)**
**Neuroendocrine neoplasm**							
20-29	1,209	1990-2004	0.50 (-10.32, 3.92)	2004-2020	10.02 (7.09, 20.11)		
30-39	879	1990-2008	1.81 (-0.69, 3.62)	2008-2011	34.39 (10.40, 45.99)	2011-2020	3.42 (-6.70, 7.95)
40-49	680	1990-2020	6.88 (5.40, 8.43)				
50-59	511	1990-2020	8.31 (5.94, 10.76)				

60-69	441	1990-2020	4.77 (2.97, 6.67)				
70-79	278	1990-2020	6.62 (3.68, 9.66)				
80-89	133	2000-2020	6.26 (1.60, 10.98)				
90+	11	1990-2020					
**Mucinous adenocarcinoma**							
20-29	39	1990-2020					
30-39	118	1999-2020	6.28 (2.27, 10.63)				
40-49	297	1990-2020	6.11 (3.99, 8.36)				
50-59	418	1990-1996	20.59 (7.06, 86.90)	1996-2020	4.74 (-16.38, 6.57)		
60-69	467	1990-2020	6.97 (4.57, 9.47)				
70-79	411	1990-2020	5.10 (3.48, 6.81)				
80-89	184	1992-2020	2.75 (0.39, 5.31)				
90+	21	1990-2020					
**Adenocarcinoma, NOS**							
20-29	32	1990-2020					
30-39	92	1997-2020	5.64 (1.75, 9.58)				
40-49	253	1991-2020	6.28 (3.86, 8.88)				
50-59	291	1990-2020	6.15 (4.06, 8.36)				
60-69	344	1990-2020	3.13 (1.44, 4.89)				
70-79	292	1990-2020	3.40 (1.68, 5.16)				
80-89	119	1999-2020	6.08 (1.45, 11.13)				
90+	16	1990-2020					

APC, annual percentage change; CI, confidence interval; *n*, number of cases; NOS, not otherwise specified.

Table S7: Temporal trends in proximal colon tumour incidence rates by age, histology, and calendar period of diagnosis.

**Age, years**	***n***	**Trend 1**		**Trend 2**		**Trend 3**	
		**Period**	**APC (95% CI)**	**Period**	**APC (95% CI)**	**Period**	**APC (95% CI)**
**Adenocarcinoma, NOS**							
20-29	341	1990-2020	4.50 (2.77, 6.32)				
30-39	1,339	1990-2009	1.90 (-8.38, 3.89)	2009-2020	6.77 (3.01, 27.17)		
40-49	3,862	1990-2011	-1.44 (-2.99, -0.80)	2011-2020	2.51 (0.19, 11.07)		
50-59	11,079	1990-2013	-2.10 (-3.78, -1.66)	2013-2020	1.07 (-1.39, 9.57)		

60-69	23,951	1990-2000	2.02 (0.95, 3.62)	2000-2020	-2.17 (-2.65, -1.80)		
70-79	34,870	1990-2001	2.89 (2.25, 3.90)	2001-2016	-0.12 (-0.64, 0.37)	2016-2020	-6.90 (-10.62, -4.39)
80-89	24,899	1990-2011	1.69 (1.38, 2.24)	2011-2020	-0.47 (-2.86, 0.61)		
90+	3,174	1990-2020	0.68 (0.20, 1.17)				
**Mucinous adenocarcinoma**							
20-29	79	1990-2020					
30-39	288	1990-2020	1.32 (-0.13, 2.85)				
40-49	809	1990-2020	-1.21 (-2.27, -0.08)				
50-59	1,940	1990-2020	-2.95 (-3.58, -2.29)				
60-69	4,481	1990-2003	1.64 (0.41, 3.30)	2003-2020	-5.35 (-6.32, -4.56)		
70-79	6,541	1990-2004	4.11 (3.16, 5.55)	2004-2016	-2.97 (-4.30, -1.25)	2016-2020	-12.88 (-21.35, -8.22)
80-89	4,481	1990-2008	2.55 (1.55, 4.57)	2008-2020	-2.12 (-5.77, -0.36)		
90+	519	1990-2020	0.90 (-0.75, 2.62)				
**Adenocarcinoma in a polyp**							
20-29	30	1990-2020					
30-39	158	1992-2020	3.52 (0.75, 6.38)				
40-49	471	1990-2000	9.97 (3.70, 68.86)	2000-2020	0.88 (-21.64, 3.17)		
50-59	1,543	1990-1995	16.72 (5.38, 53.86)	1995-2010	2.86 (-10.24, 5.83)	2010-2020	-5.04 (-20.18, 1.93)
60-69	3,521	1990-1994	24.81 (14.90, 44.94)	1994-2011	4.07 (2.69, 5.51)	2011-2020	-6.01 (-9.78, -3.39)
70-79	4,942	1990-2000	9.64 (7.74, 13.58)	2000-2013	3.15 (1.24, 4.80)	2013-2020	-7.47 (-11.90, -4.63)
80-89	2,879	1990-1994	24.24 (9.82, 56.40)	1994-2010	5.15 (1.63, 7.70)	2010-2020	-3.71 (-13.54, -0.18)
90+	273	1992-2020	1.29 (-1.19, 3.88)				

APC, annual percentage change; CI, confidence interval; *n*, number of cases; NOS, not otherwise specified.

Table S8: Temporal trends in distal colon tumour incidence rates by age, histology, and calendar period of diagnosis.

Age	*n*	Trend 1		Trend 2		Trend 3		Trend 4		Trend 5	
		Period	APC (95% CI)	Period	APC (95% CI)	Period	APC (95% CI)	Period	APC (95% CI)	Period	APC (95% CI)
**Adenocarcinoma, NOS**											
20-29	277	1990-2011	8.47 (6.38, 13.09)	2011-2020	-3.82 (-20.83, 2.87)						
30-39	1,247	1990-1995	-8.28 (-23.63, -0.63)	1995-2015	3.29 (0.97, 5.16)	2015-2020	13.92 (5.32, 36.87)				
40-49	4,313	1990-2003	-2.39 (-4.66, -1.08)	2003-2020	2.64 (1.71, 4.09)						
50-59	11,121	1990-2001	-3.42 (-6.00, -2.30)	2001-2020	-0.87 (-1.37, 0.11)						

60-69	18,782	1990-2002	-0.65 (-1.70, 2.67)	2002-2020	-3.04 (-4.47, -2.45)						
70-79	20,473	1990-2010	-0.31 (-0.77, 0.22)	2010-2013	-9.34 (-12.37, -0.53)	2013-2016	5.12 (-2.52, 9.32)	2016-2020	-12.54 (-17.39, -9.98)		
80-89	10,739	1990-2017	-0.78 (-1.11, 2.80)	2017-2020	-7.42 (-16.42, -1.16)						
90+	1,202	1990-2020	-2.16 (-2.82, -1.47)								
**Mucinous adenocarcinoma**											
20-29	33	2005-2020	-6.54 (-12.15, -0.59)								
30-39	121	1990-2020	1.08 (-1.32, 3.62)								
40-49	361	1990-2020	-1.26 (-2.20, -0.30)								
50-59	897	1990-2020	-3.76 (-4.66, -2.81)								
60-69	1,636	1990-2006	-1.40 (-2.88, 1.49)	2006-2020	-7.16 (-10.45, -5.38)						
70-79	1,831	1990-2005	1.60 (-0.26, 4.89)	2005-2020	-5.86 (-8.55, -4.07)						
80-89	1,001	1990-1992	45.61 (4.37, 91.40)	1992-2020	-1.69 (-2.98, -0.93)						
90+	82	2000-2002	-51.37 (-67.72, -3.28)	2002-2005	67.15 (6.58, 110.89)	2005-2020	-5.66 (-10.34, -1.58)				
**Adenocarcinoma in a polyp**											
20-29	36	2007-2020	-5.21 (-17.00, 7.66)								
30-39	252	1990-2020	5.41 (3.00, 7.93)								
40-49	843	1990-2000	8.99 (3.27, 54.82)	2000-2020	0.31 (-16.46, 2.32)						
50-59	2,693	1990-1992	38.24 (10.85, 64.21)	1992-2006	1.71 (-8.31, 2.92)	2006-2010	16.75 (6.34, 29.16)	2010-2016	-14.10 (-25.92, -9.57)	2016-2020	12.72 (2.40, 37.52)
60-69	4,656	1990-2011	4.42 (3.16, 5.94)	2011-2020	-9.48 (-14.68, -5.77)						
70-79	4,378	1990-2011	4.67 (3.80, 5.63)	2011-2020	-9.68 (-12.80, -7.08)						
80-89	1,859	1990-1992	74.83 (34.42, 111.40)	1992-2011	2.90 (1.54, 4.25)	2011-2020	-7.85 (-12.67, -4.73)				
90+	144	1990-1994	32.78 (0.36, 148.07)	1994-2020	-3.26 (-13.34, -1.21)						

APC, annual percentage change; CI, confidence interval; *n*, number of cases; NOS, not otherwise specified.

Table S9: Temporal trends in rectal tumour incidence rates by age, histology, and calendar period of diagnosis.

Age, years	*n*	Trend 1		Trend 2		Trend 3	
		Period	APC (95% CI)	Period	APC (95% CI)	Period	APC (95% CI)
**Adenocarcinoma, NOS**							
20-29	390	1990-2013	7.25 (5.59, 16.01)	2013-2020	-5.01 (-30.60, 3.98)		

30-39	2,062	1990-2020	3.47 (2.72, 4.23)				
40-49	6,854	1990-2020	0.53 (0.24, 0.84)				
50-59	18,089	1990-2008	-1.04 (-1.36, 0.06)	2008-2013	-3.75 (-7.37, -1.70)	2013-2020	1.83 (0.39, 5.79)
60-69	28,104	1990-1994	3.76 (-0.12, 11.06)	1994-2007	-1.09 (-3.66, -0.28)	2007-2020	-3.42 (-5.96, -2.58)
70-79	27,654	1990-2001	0.78 (0.06, 2.01)	2001-2016	-2.10 (-2.73, -1.54)	2016-2020	-9.00 (-13.94, -6.35)
80-89	14,033	1990-2001	0.17 (-1.13, 5.73)	2001-2018	-1.55 (-2.31, 0.55)	2018-2020	-11.64 (-17.89, -2.18)
90+	1,745	1990-2020	-0.99 (-1.77, -0.16)				
**Mucinous adenocarcinoma**							
20-29	39	1990-2020					
30-39	147	1990-2020	-0.20 (-2.52, 2.27)				
40-49	451	1990-2020	-2.95 (-4.41, -1.46)				
50-59	1,179	1990-2010	-1.82 (-3.30, -0.02)	2010-2013	-23.06 (-29.37, -3.61)	2013-2020	1.25 (-5.77, 23.98)
60-69	1,969	1990-2004	0.48 (-1.72, 4.87)	2004-2020	-7.10 (-9.92, -5.32)		
70-79	2,056	1990-2012	-1.75 (-2.64, -0.61)	2012-2020	-12.75 (-17.65, -9.24)		
80-89	1,011	1990-2007	-1.29 (-2.93, 2.74)	2007-2020	-7.64 (-12.81, -5.24)		
90+	108	1990-2007	1.70 (-3.53, 52.40)	2007-2020	-12.62 (-46.00, -5.49)		
**Adenocarcinoma in a polyp**							
20-29	57	1997-2019	-0.22 (-6.52, 6.35)				
30-39	311	1990-1997	18.40 (6.74, 59.46)	1997-2020	-0.05 (-3.66, 1.81)		
40-49	1,047	1990-2011	3.76 (2.17, 9.84)	2011-2020	-5.22 (-21.54, 0.03)		
50-59	3,206	1990-2010	3.18 (1.90, 4.92)	2010-2020	-6.92 (-11.62, -3.75)		
60-69	5,251	1990-1992	25.79 (4.03, 42.33)	1992-2010	2.45 (-2.29, 3.44)	2010-2020	-6.95 (-10.08, -4.57)
70-79	5,206	1990-1997	9.87 (6.96, 15.66)	1997-2011	1.03 (-0.32, 2.35)	2011-2020	-8.61 (-11.02, -6.74)
80-89	2,407	1990-2000	5.68 (2.50, 19.46)	2000-2012	0.06 (-15.88, 2.47)	2012-2020	-10.27 (-19.62, -2.71)
90+	269	1991-2010	0.61 (-2.14, 22.25)	2010-2020	-10.79 (-38.20, -4.06)		
**Neuroendocrine neoplasm**							
20-29	94	2002-2020	11.14 (3.79, 19.07)				
30-39	409	1990-2020	7.54 (6.01, 9.13)				
40-49	784	1990-1993	79.09 (22.67, 237.48)	1993-2020	6.22 (3.69, 8.02)		
50-59	1,068	1990-2008	10.07 (6.94, 98.98)	2008-2020	1.71 (-42.91, 7.05)		
60-69	997	1990-1996	-7.59 (-23.27, -0.15)	1996-1999	43.73 (16.13, 59.24)	1999-2020	4.15 (2.29, 5.44)
70-79	614	1990-2020	5.89 (4.37, 7.47)				

80-89	150	1990-2020	4.34 (1.63, 7.33)
90+	16	1990-2020	

APC, annual percentage change; CI, confidence interval; *n*, number of cases; NOS, not otherwise specified.

Table S10: Temporal trends in anal tumour incidence rates by age, histology, and calendar period of diagnosis.

Age, years	*n*	Trend 1		Trend 2	
		Period	APC (95% CI)	Period	APC (95% CI)
**Squamous cell carcinoma**					
20-29	22	1990-2020			
30-39	197	1990-2020	-0.83 (-2.85, 1.29)		
40-49	903	1990-2010	5.64 (4.14, 8.13)	2010-2020	-3.71 (-10.54, 0.14)
50-59	1,787	1990-2020	4.40 (3.39, 5.41)		
60-69	1,937	1990-1994	-4.70 (-18.46, 3.87)	1994-2020	4.34 (3.38, 7.93)
70-79	1,493	1990-2008	-0.26 (-3.02, 1.16)	2008-2020	6.57 (3.89, 13.62)
80-89	682	1990-2020	1.64 (0.54, 2.77)		
90+	138	1992-2020	0.52 (-1.78, 2.96)		
**Adenocarcinoma, NOS**					
20-29	9	1990-2020			
30-39	29	1990-2020			
40-49	83	1990-2020			
50-59	229	1990-2020	-1.35 (-3.75, 1.16)		
60-69	364	1990-1993	46.13 (11.43, 135.71)	1993-2020	-1.65 (-3.31, -0.45)
70-79	417	1990-2020	-2.63 (-4.23, -0.97)		
80-89	307	1990-1993	-24.40 (-45.66, -1.57)	1993-2020	-0.69 (-2.45, 5.79)
90+	74	1990-2020			

APC, annual percentage change; CI, confidence interval; *n*, number of cases; NOS, not otherwise specified.

Table S11: Cumulative excess early-onset cancers attributable to rising incidence rates between 1990 and 2020, by sex and after excluding neuroendocrine neoplasms.

Age, years	Males						Females					
	AAPC (95% CI)	Recorded cases	Expected cases (95% UI)[a]	Excess cases (95% UI)	AER (95% UI)[b]	P-score (%, 95% UI)	AAPC (95% CI)	Recorded cases	Expected cases (95% UI)[a]	Excess cases (95% UI)	AER (95% UI)[b]	P-score (%, 95% UI)

**Appendix**												
20-29[c]	-0.68 (-8.59, 7.89)	35	37 (0, 52)	-2 (-17, 35)	-0.01 (-0.06, 0.12)	-5.39 (-32.7, Inf)	–	–	–	–	–	–
30-39[d]	5.76 (-3.76, 15.75)	111	69 (0, 126)	42 (-15, 111)	0.13 (-0.04, 0.33)	60.89 (-11.56, Inf)	7.06 (-0.21, 14.81)	112	55 (0, 113)	57 (-1, 112)	0.16 (-0.002, 0.31)	104.20 (-0.83, Inf)
40-49[e]	7.16 (4.61, 9.82)	298	102 (0, 206)	196 (92, 298)	0.44 (0.21, 0.67)	190.88 (44.56, Inf)	5.12 (3.09, 7.31)	313	146 (2, 233)	167 (80, 311)	0.38 (0.18, 0.70)	114.73 (34.62, 12,744.60)
Total	–	444	208 (0, 384)	236 (60, 444)	0.22 (0.06, 0.42)	113.46 (15.63, Inf)	–	425	201 (2, 346)	224 (79, 423)	0.28 (0.10, 0.69)	111.44 (22.83, 21,150.00)
**Colon**												
20-29	3.36 (1.69, 5.14)	459	266 (94, 378)	193 (81, 365)	0.41 (0.17, 0.77)	72.58 (21.34, 388.74)	3.92 (2.59, 5.31)	480	250 (112, 349)	230 (131, 368)	0.50 (0.28, 0.79)	92.09 (37.40, 328.60)
30-39	2.67 (2.01, 3.35)	1,755	1,135 (915, 1,320)	620 (435, 840)	1.31 (0.92, 1.77)	54.63 (32.91, 91.89)	2.56 (1.52, 3.66)	1,970	1,310 (903, 1,619)	660 (351, 1,067)	1.38 (0.73, 2.23)	50.40 (21.70, 118.05)
40-49	-0.18 (-0.76, 0.41)	5,863	6,036 (5,459, 6,536)	-173 (-673, 404)	-0.39 (-1.50, 0.90)	-2.86 (-10.30, 7.39)	-0.38 (-0.87, 0.13)	5,915	6,280 (5,787, 6,713)	-365 (-798, 128)	-0.81 (-1.76, 0.28)	-5.81 (-11.89, 2.21)
Total	–	8,077	7,437 (6,468, 8,234)	640 (-157, 1,609)	0.46 (-0.11, 1.15)	8.61 (-1.91, 24.88)	–	8,365	7,840 (6,802, 8,681)	525 (-316, 1,563)	0.38 (-0.23, 1.12)	6.70 (-3.64, 22.98)
**Rectum**												
20-29	5.83 (3.70, 8.04)	275	105 (0, 192)	170 (83, 275)	0.36 (0.18, 0.58)	163.00 (43.54, Inf)	3.70 (1.41, 6.13)	244	138 (9, 213)	106 (31, 235)	0.23 (0.07, 0.51)	76.30 (14.70, 2,650.28)
30-39	3.75 (2.90, 4.63)	1,345	726 (497, 909)	619 (436, 848)	1.31 (0.92, 1.79)	85.39 (47.91, 170.63)	2.32 (1.47, 3.20)	1,273	875 (668, 1,042)	398 (231, 605)	0.83 (0.48, 1.27)	45.41 (22.13, 90.66)
40-49	0.46 (0.05, 0.88)	4,876	4,531 (4,192, 4,837)	345 (39, 684)	0.77 (0.09, 1.53)	7.61 (0.81, 16.32)	0.56 (0.13, 1.00)	3,810	3,483 (3,199, 3,736)	327 (74, 611)	0.72 (0.16, 1.35)	9.38 (1.98, 19.11)
Total	–	6,496	5,362 (4,689, 5,938)	1,134 (558, 1,807)	0.81 (0.40, 1.32)	21.15 (9.40, 38.54)	–	5,327	4,496 (3,876, 4,991)	831 (336, 1,451)	0.59 (0.24, 1.04)	18.48 (6.73, 37.44)
**Anus**												
30-39[f]	-1.67 (-4.53, 1.42)	125	156 (90, 190)	-31 (-65, 35)	-0.06 (-0.14, 0.07)	-19.71 (-34.26, 39.39)	-1.48 (-5.09, 2.25)	93	106 (67, 130)	-13 (-37, 26)	-0.04 (-0.12, 0.08)	-12.59 (-28.72, 37.98)
40-49	1.71 (0.37, 3.08)	436	333 (221, 417)	103 (19, 215)	0.23 (0.04, 0.48)	30.79 (4.64, 97.56)	3.13 (1.81, 4.49)	619	373 (206, 496)	246 (123, 413)	0.54 (0.27, 0.91)	66.12 (24.72, 200.94)
Total	–	561	489 (311, 607)	72 (-46, 250)	0.08 (-0.05, 0.27)	14.72 (-7.58, 80.39)	–	712	479 (273, 626)	233 (86, 439)	0.30 (0.11, 0.57)	48.64 (13.74, 160.81)

CI, confidence interval; AAPC, average annual percentage change; UI, uncertainty interval; AER, absolute excess risk.

[a]Expected cases with an AAPC of 0% from 1990.
[b]Per 100,000 persons-years.
[c]Restricted to 2000-2017 period to exclude runs of zeros.
[d]Restricted to 2000-2020 period in males and 1998-2020 period in females to exclude runs of zeros.
[e]Restricted to 1991-2020 period in females to exclude runs of zeros.
[f]Restricted to 2001-2020 period in females to exclude runs of zeros.

Table S12: Cumulative excess early-onset tumours attributable to rising incidence rates between 1990 and 2020, for all histological subtypes combined.

Age, years	AAPC (95% CI)	Recorded cases	Expected cases (95% UI)[a]	Excess cases (95% UI)	AER (95% UI)[b]	P-score (%, 95% UI)
**Appendix**						
20-29	5.5 (4.3, 6.7)	1,290	537 (212, 775)	753 (515, 1,078)	0.8 (0.6, 1.2)	140.3 (66.5, 507.9)
30-39	6.2 (5.1, 7.3)	1,123	411 (154, 608)	712 (515, 969)	0.7 (0.5, 1.0)	173.1 (84.6, 628.8)
40-49	6.6 (5.5, 7.8)	1,291	408 (82, 654)	883 (637, 1,209)	1.0 (0.7, 1.3)	216.5 (97.5, 1,477.5)
Total	–	3,704	1,356 (448, 2,037)	2,348 (1,667, 3,256)	0.8 (0.6, 1.2)	173.2 (81.8, 726.8)
**Colon**						
20-29	3.6 (2.4, 4.8)	958	524 (283, 700)	434 (258, 675)	0.5 (0.3, 0.7)	82.9 (36.8, 238.1)
30-39	2.6 (2.0, 3.4)	3,808	2,481 (1,978, 2,898)	1,327 (910, 1,830)	1.4 (1.0, 1.9)	53.5 (31.4, 92.5)
40-49	-0.2 (-0.7, 0.2)	11,949	12,405 (11,487, 13,207)	-456 (-1,258, 462)	-0.5 (-1.4, 0.5)	-3.7 (-9.5, 4.0)
Total	–	16,715	15,410 (13,748, 16,805)	1,305 (-90, 2,967)	0.5 (0.0, 1.1)	8.5 (-0.5, 21.6)
**Rectum**						
20-29	5.5 (4.1, 7.0)	622	246 (42, 385)	376 (237, 580)	0.4 (0.2, 0.6)	152.6 (61.4, 1,363.9)
30-39	3.5 (3.0, 3.9)	3,027	1,701 (1,443, 1,931)	1,326 (1,096, 1,584)	1.4 (1.1, 1.7)	79.1 (65.4, 94.4)
40-49	0.9 (0.6, 1.2)	9,470	8,162 (7,631, 8,630)	1,308 (840, 1,839)	1.5 (0.9, 2.0)	16.0 (9.7, 24.1)
Total	–	13,119	10,109 (9,116, 10,946)	3,010 (2,173, 4,003)	1.1 (0.8, 1.4)	29.8 (19.9, 43.9)
**Anus**						
30-39	-0.1 (-1.7, 1.5)	258	262 (188, 313)	-4 (-55, 70)	0.0 (-0.1, 0.1)	-1.5 (-17.6, 37.3)
40-49	2.7 (1.7, 3.8)	1,075	686 (469, 855)	389 (220, 606)	0.4 (0.2, 0.7)	57.6 (32.5, 89.8)
Total	–	1,333	984 (693, 1,204)	385 (165, 676)	0.2 (0.1, 0.4)	39.1 (13.7, 56.1)

CI, confidence interval; AAPC, average annual percentage change; UI, uncertainty interval; AER, absolute excess risk.
[a]Expected cases with an AAPC of 0% from 1990.
[b]Per 100,000 persons-years.

Table S13: Cumulative excess early-onset tumours attributable to rising incidence rates between 1990 and 2020, by sex and for all histological subtypes combined.

Age, years	Males						Females					
	AAPC (95% CI)	Recorded cases	Expected cases (95% UI)[a]	Excess cases (95% UI)	AER (95% UI)[b]	P-score (%, 95% UI)	AAPC (95% CI)	Recorded cases	Expected cases (95% UI)[a]	Excess cases (95% UI)	AER (95% UI)[b]	P-score (%, 95% UI)
**Appendix**												
20-29	5.59 (3.59, 7.65)	479	201 (0, 339)	278 (140, 479)	0.58 (0.29, 1.01)	137.83 (41.22, Inf)	5.57 (4.07, 7.18)	811	343 (71, 526)	468 (285, 740)	1.01 (0.61, 1.59)	136.09 (54.04, 1,045.42)
30-39	7.73 (5.61, 9.87)	453	127 (0, 226)	326 (180, 453)	0.69 (0.38, 0.95)	256.56 (65.91, Inf)	5.69 (4.29, 7.15)	670	265 (57, 413)	405 (257, 613)	0.85 (0.54, 1.28)	152.78 (62.42, 1,070.31)
40-49	7.80 (5.79, 9.83)	558	163 (0, 331)	395 (227, 558)	0.88 (0.51, 1.24)	242.88 (68.62, Inf)	6.10 (4.69, 7.62)	733	261 (15, 427)	472 (306, 718)	1.04 (0.68, 1.59)	181.35 (71.53, 4,701.9)
Total	–	1,490	491 (0, 896)	999 (547, 1,490)	0.71 (0.39, 1.07)	203.46 (61.05, Inf)	–	2,214	869 (143, 1,366)	1,345 (848, 2,071)	0.96 (0.61, 1.48)	154.78 (62.08, 1,448.25)
**Colon**												
20-29	3.50 (2.01, 5.08)	471	266 (112, 372)	205 (99, 359)	0.43 (0.21, 0.75)	76.92 (26.76, 320.16)	3.90 (2.62, 5.24)	487	254 (120, 352)	233 (135, 367)	0.50 (0.29, 0.79)	91.54 (38.20, 306.34)
30-39	2.77 (2.05, 3.53)	1,787	1,138 (887, 1,345)	649 (442, 900)	1.37 (0.93, 1.90)	56.96 (32.83, 101.47)	2.64 (1.55, 3.78)	2,021	1,327 (889, 1,660)	694 (361, 1,132)	1.45 (0.76, 2.37)	52.30 (21.78, 127.28)
40-49	-0.14 (-0.73, 0.47)	5,958	6,184 (5,729, 6,581)	-226 (-623, 229)	-0.50 (-1.39, 0.51)	-3.65 (-9.47, 3.99)	-0.32 (-0.80, 0.18)	5,991	6,300 (5,810, 6,736)	-309 (-745, 181)	-0.7 (-1.6, 0.4)	-4.9 (-11.1, 3.1)
Total	–	8,216	7,589 (6,728, 8,298)	628 (-82, 1,488)	0.45 (-0.06, 1.06)	8.27 (-0.99, 22.11)	–	8,499	7,882 (6,820, 8,748)	617 (-249, 1,679)	0.44 (-0.18, 1.20)	7.83 (-2.85, 24.62)
**Rectum**												
20-29	6.81 (4.77, 8.92)	332	106 (0, 210)	226 (122, 332)	0.47 (0.26, 0.70)	212.66 (58.43, Inf)	4.49 (2.58, 6.51)	290	144 (18, 223)	146 (67, 272)	0.31 (0.15, 0.59)	101.62 (30.26, 1,514.50)
30-39	4.22 (3.60, 4.88)	1,570	773 (574, 938)	797 (632, 996)	1.68 (1.33, 2.10)	103.0 (67.44, 173.75)	2.77 (1.87, 3.69)	1,457	931 (676, 1,134)	526 (323, 781)	1.10 (0.67, 1.63)	56.58 (28.44, 115.57)
40-49	0.90 (0.47, 1.34)	5,283	4,573 (4,176, 4,927)	710 (356, 1,107)	1.58 (0.79, 2.47)	15.52 (7.22, 26.50)	0.97 (0.55, 1.41)	4,187	3,579 (3,261, 3,856)	608 (331, 926)	1.34 (0.73, 2.05)	16.99 (8.57, 28.39)
Total	–	7,185	5,452 (4,750, 6,075)	1,733 (1,110, 2,135)	1.24 (0.79, 1.78)	31.79 (18.27, 44.95)	–	5,934	4,654 (3,955, 5,213)	1,280 (721, 1,979)	0.92 (0.52, 1.42)	27.50 (13.83, 50.04)
**Anus**												
30-39[c]	-1.28 (-4.23, 1.88)	128	152 (80, 188)	-24 (-60, 48)	-0.05 (-0.13, 0.10)	-15.55 (-31.96, 59.27)	-1.74 (-5.48, 2.07)	96	112 (71, 138)	-16 (-42, 25)	-0.05 (-0.13, 0.08)	-14.64 (-30.46, 34.83)
40-49	1.86 (0.62, 3.14)	444	332 (227, 411)	112 (33, 217)	0.25 (0.07, 0.48)	33.75 (8.03, 95.69)	3.20 (1.85, 4.58)	631	375 (201, 503)	256 (128, 430)	0.56 (0.28, 0.95)	68.36 (25.50, 214.63)
Total	–	572	484 (307, 599)	88 (-27, 265)	0.10 (-0.03, 0.29)	18.18 (-4.51, 86.32)	–	727	487 (272, 641)	240 (86, 455)	0.26 (0.09, 0.49)	49.28 (13.42, 167.28)

CI, confidence interval; AAPC, average annual percentage change; UI, uncertainty interval; AER, absolute excess risk.

[a]Expected cases with an AAPC of 0% from 1990.

[b]Per 100,000 persons-years.

[c]Restricted to 2001-2020 period in females to exclude runs of zeros.

Table S14: Ratio of age-specific cancer incidence rates between 1990 and 2019, by site, sex, and birth cohort after excluding neuroendocrine neoplasms.

Birth cohort	IRR (95% CI)				
	Appendix	Proximal colon	Distal colon	Rectum	Anus
**Persons**					
1900	0.18 (0.08-0.4)	0.87 (0.75-1.01)	1.96 (1.6-2.4)	1.97 (1.79-2.17)	0.73 (0.49-1.09)
1910	0.19 (0.14-0.27)	0.94 (0.89-1)	1.56 (1.45-1.69)	1.68 (1.62-1.75)	0.59 (0.49-0.71)
1920	0.23 (0.19-0.29)	1.07 (1.02-1.12)	1.47 (1.39-1.55)	1.51 (1.47-1.55)	0.62 (0.54-0.7)
1930	0.39 (0.33-0.46)	1.23 (1.18-1.28)	1.42 (1.35-1.48)	1.37 (1.34-1.4)	0.58 (0.52-0.65)
1940	0.61 (0.54-0.69)	1.21 (1.17-1.26)	1.22 (1.17-1.27)	1.18 (1.16-1.2)	0.74 (0.68-0.81)
1950	Ref	Ref	Ref	Ref	Ref
1960	1.82 (1.59-2.08)	0.88 (0.83-0.93)	0.95 (0.9-1)	0.94 (0.91-0.96)	1.42 (1.29-1.57)
1970	3.36 (2.77-4.07)	0.93 (0.84-1.02)	1.15 (1.04-1.26)	1.12 (1.07-1.17)	1.63 (1.38-1.91)
1980	7.04 (5.19-9.55)	1.52 (1.28-1.81)	1.88 (1.57-2.25)	1.7 (1.56-1.85)	1.4 (1-1.96)
1990	8.87 (5.29-14.89)	2.26 (1.59-3.2)	2.77 (1.89-4.07)	2.76 (2.29-3.33)	1.95 (0.85-4.48)
P_{wald}	1×10^{-85}	2×10^{-79}	1×10^{-71}	0	2×10^{-33}
**Males**					
1900	0.06 (0.01-0.49)	0.88 (0.68-1.15)	1.94 (1.42-2.64)	1.99 (1.63-2.43)	0.67 (0.32-1.37)
1910	0.15 (0.09-0.25)	1 (0.92-1.1)	1.59 (1.44-1.75)	1.67 (1.56-1.78)	0.68 (0.52-0.87)
1920	0.2 (0.15-0.28)	1.1 (1.03-1.17)	1.44 (1.35-1.53)	1.51 (1.45-1.58)	0.78 (0.66-0.92)
1930	0.35 (0.28-0.44)	1.22 (1.16-1.29)	1.38 (1.31-1.45)	1.37 (1.32-1.42)	0.76 (0.67-0.86)
1940	0.48 (0.4-0.58)	1.18 (1.12-1.24)	1.19 (1.14-1.25)	1.17 (1.13-1.2)	0.83 (0.75-0.93)
1950	Ref	Ref	Ref	Ref	Ref
1960	1.88 (1.54-2.29)	0.88 (0.81-0.95)	0.95 (0.88-1.01)	0.93 (0.89-0.97)	1.3 (1.15-1.47)
1970	4.05 (3.05-5.37)	0.98 (0.86-1.12)	1.17 (1.03-1.33)	1.13 (1.04-1.21)	1.42 (1.16-1.73)
1980	9.12 (5.86-14.19)	1.39 (1.09-1.77)	2.09 (1.63-2.67)	1.87 (1.61-2.17)	1 (0.67-1.51)
1990	8.33 (4.03-17.23)	2.01 (1.23-3.28)	3.31 (1.99-5.49)	2.83 (2.03-3.95)	1.36 (0.5-3.71)
P_{wald}	2×10^{-46}	8×10^{-25}	2×10^{-45}	4×10^{-123}	3×10^{-8}
**Females**					
1900	0.26 (0.1-0.67)	0.87 (0.78-0.98)	2.2 (1.9-2.54)	2.21 (1.93-2.52)	0.65 (0.36-1.19)
1910	0.24 (0.16-0.36)	0.91 (0.87-0.96)	1.69 (1.59-1.81)	1.87 (1.76-1.99)	0.51 (0.37-0.69)
1920	0.26 (0.2-0.34)	1.05 (1.01-1.1)	1.59 (1.51-1.67)	1.6 (1.52-1.68)	0.51 (0.4-0.64)
1930	0.43 (0.35-0.53)	1.24 (1.19-1.29)	1.49 (1.43-1.55)	1.39 (1.34-1.45)	0.47 (0.39-0.57)
1940	0.74 (0.63-0.88)	1.25 (1.21-1.29)	1.25 (1.2-1.29)	1.18 (1.14-1.22)	0.68 (0.58-0.79)
1950	Ref	Ref	Ref	Ref	Ref
1960	1.76 (1.47-2.11)	0.88 (0.84-0.93)	0.95 (0.91-1)	0.95 (0.91-1)	1.5 (1.26-1.78)
1970	2.81 (2.16-3.66)	0.87 (0.8-0.96)	1.13 (1.05-1.22)	1.12 (1.04-1.2)	1.78 (1.34-2.36)
1980	5.42 (3.53-8.31)	1.66 (1.41-1.95)	1.72 (1.5-1.98)	1.55 (1.36-1.76)	1.87 (1.02-3.41)
1990	9.54 (4.47-20.34)	2.52 (1.82-3.47)	2.37 (1.75-3.21)	2.72 (2.03-3.65)	2.43 (0.54-10.82)
P_{wald}	1×10^{-37}	3×10^{-130}	7×10^{-115}	6×10^{-123}	8×10^{-16}

IRR, incidence rate ratio; CI, confidence interval; Ref, reference.

Table S15: Ratio of age-specific tumour incidence rates between 1990 and 2019, by site, sex, and birth cohort for all histological subtypes combined.

Birth cohort	IRR (95% CI)

	Appendix	Proximal colon	Distal colon	Rectum	Anus
**Persons**					
1900	0.12 (0.02-0.6)	0.86 (0.75-0.98)	1.95 (1.59-2.38)	1.89 (1.72-2.07)	0.74 (0.5-1.1)
1910	0.15 (0.09-0.27)	0.93 (0.88-0.98)	1.55 (1.44-1.68)	1.61 (1.55-1.67)	0.59 (0.49-0.71)
1920	0.22 (0.15-0.31)	1.05 (1.01-1.1)	1.46 (1.38-1.54)	1.45 (1.41-1.49)	0.62 (0.54-0.7)
1930	0.4 (0.31-0.52)	1.22 (1.17-1.26)	1.41 (1.35-1.47)	1.32 (1.29-1.35)	0.58 (0.52-0.65)
1940	0.59 (0.48-0.73)	1.21 (1.17-1.25)	1.22 (1.17-1.27)	1.15 (1.13-1.17)	0.74 (0.68-0.81)
1950	Ref	Ref	Ref	Ref	Ref
1960	1.84 (1.5-2.26)	0.89 (0.84-0.93)	0.95 (0.9-1)	0.96 (0.94-0.99)	1.42 (1.28-1.57)
1970	3.51 (2.71-4.56)	0.94 (0.86-1.03)	1.16 (1.06-1.28)	1.21 (1.16-1.26)	1.66 (1.41-1.95)
1980	6.44 (4.71-8.81)	1.55 (1.32-1.83)	1.91 (1.59-2.29)	1.94 (1.79-2.09)	1.45 (1.04-2.03)
1990	12.59 (8.64-18.34)	2.31 (1.67-3.19)	2.85 (1.94-4.18)	3.53 (2.97-4.2)	2.01 (0.87-4.64)
P_{wald}	1×10^{-51}	3×10^{-89}	2×10^{-69}	7×10^{-313}	4×10^{-33}
**Males**					
1900	0.04 (0.01-0.33)	0.87 (0.67-1.12)	1.92 (1.4-2.63)	1.93 (1.6-2.31)	0.68 (0.33-1.39)
1910	0.11 (0.07-0.18)	0.99 (0.9-1.08)	1.58 (1.43-1.75)	1.6 (1.51-1.7)	0.69 (0.53-0.89)
1920	0.19 (0.15-0.25)	1.08 (1.01-1.15)	1.43 (1.34-1.53)	1.46 (1.4-1.52)	0.78 (0.67-0.92)
1930	0.37 (0.31-0.45)	1.21 (1.15-1.28)	1.37 (1.3-1.44)	1.33 (1.29-1.37)	0.76 (0.67-0.86)
1940	0.49 (0.42-0.57)	1.17 (1.12-1.23)	1.19 (1.14-1.25)	1.15 (1.11-1.18)	0.83 (0.75-0.93)
1950	Ref	Ref	Ref	Ref	Ref
1960	1.95 (1.67-2.28)	0.88 (0.82-0.95)	0.95 (0.89-1.02)	0.96 (0.92-1)	1.29 (1.14-1.46)
1970	4.45 (3.63-5.46)	0.99 (0.87-1.12)	1.19 (1.04-1.35)	1.23 (1.15-1.31)	1.42 (1.16-1.73)
1980	9.02 (7.03-11.58)	1.42 (1.13-1.79)	2.13 (1.66-2.73)	2.13 (1.88-2.42)	1.07 (0.72-1.6)
1990	15.93 (11.72-21.64)	2.1 (1.31-3.35)	3.43 (2.06-5.7)	3.72 (2.8-4.94)	1.42 (0.52-3.86)
P_{wald}	5×10^{-109}	2×10^{-26}	6×10^{-44}	7×10^{-141}	8×10^{-8}
**Females**					
1900	0.17 (0.02-1.33)	0.86 (0.77-0.97)	2.18 (1.89-2.52)	2.09 (1.83-2.38)	0.66 (0.36-1.18)
1910	0.19 (0.08-0.41)	0.9 (0.86-0.95)	1.68 (1.57-1.8)	1.77 (1.66-1.88)	0.5 (0.37-0.69)
1920	0.24 (0.14-0.41)	1.04 (1-1.08)	1.58 (1.5-1.66)	1.51 (1.44-1.58)	0.5 (0.4-0.63)
1930	0.42 (0.28-0.62)	1.22 (1.18-1.27)	1.48 (1.42-1.54)	1.32 (1.27-1.38)	0.47 (0.38-0.57)
1940	0.69 (0.51-0.94)	1.24 (1.2-1.28)	1.24 (1.2-1.29)	1.15 (1.11-1.19)	0.68 (0.58-0.79)
1950	Ref	Ref	Ref	Ref	Ref
1960	1.76 (1.3-2.38)	0.89 (0.85-0.94)	0.96 (0.91-1)	0.98 (0.94-1.02)	1.5 (1.27-1.78)
1970	2.96 (2.03-4.33)	0.89 (0.81-0.98)	1.14 (1.06-1.23)	1.2 (1.12-1.28)	1.83 (1.39-2.42)
1980	5.13 (3.28-8.04)	1.69 (1.45-1.98)	1.75 (1.52-2.01)	1.75 (1.55-1.98)	1.87 (1.03-3.38)
1990	10.65 (6.25-18.15)	2.52 (1.84-3.44)	2.4 (1.77-3.25)	3.38 (2.59-4.41)	2.49 (0.56-10.95)
P_{wald}	1×10^{-19}	2×10^{-133}	4×10^{-113}	7×10^{-119}	1×10^{-16}

IRR, incidence rate ratio; CI, confidence interval; Ref, reference.

Table S16: Ratio of age-specific tumour incidence rates between 1990 and 2019, by site, histology, and birth cohort.

Birth cohort	IRR (95% CI)				
	Appendix	Proximal colon	Distal colon	Rectum	Anus
**Adenocarcinoma NOS**					
1900	0.23 (0.05-1.04)	0.92 (0.76-1.12)	2.54 (2.01-3.21)	2.01 (1.78-2.27)	3.51 (1.93-6.38)
1910	0.37 (0.23-0.59)	0.99 (0.92-1.06)	1.86 (1.71-2.03)	1.75 (1.68-1.83)	2.25 (1.62-3.13)
1920	0.34 (0.25-0.48)	1.11 (1.05-1.18)	1.68 (1.58-1.79)	1.58 (1.53-1.63)	2.18 (1.69-2.82)

1930	0.57 (0.45-0.73)	1.26 (1.2-1.33)	1.56 (1.49-1.64)	1.43 (1.39-1.46)	1.5 (1.21-1.87)
1940	0.7 (0.56-0.86)	1.23 (1.18-1.29)	1.29 (1.23-1.35)	1.21 (1.18-1.23)	1.29 (1.06-1.56)
1950	Ref	Ref	Ref	Ref	Ref
1960	1.68 (1.35-2.09)	0.86 (0.81-0.92)	0.91 (0.86-0.97)	0.92 (0.9-0.95)	0.96 (0.74-1.25)
1970	3.69 (2.73-4.97)	0.91 (0.8-1.03)	1.12 (1.01-1.24)	1.12 (1.06-1.17)	0.64 (0.4-1.03)
1980	5.94 (3.71-9.51)	1.6 (1.29-1.98)	1.87 (1.53-2.28)	1.83 (1.67-2.01)	0.71 (0.31-1.61)
1990	6.68 (2.96-15.07)	2.27 (1.47-3.51)	2.8 (1.82-4.3)	3.2 (2.57-3.97)	3.72 (0.68-20.47)
P_{wald}	6×10^{-23}	6×10^{-54}	1×10^{-99}	5×10^{-311}	3×10^{-8}
**Mucinous adenocarcinoma**					
1900	0.09 (0.02-0.4)	1.14 (0.87-1.48)	3.82 (2.35-6.21)	5.22 (3.22-8.46)	–
1910	0.14 (0.09-0.22)	1.4 (1.27-1.54)	2.99 (2.51-3.56)	4.71 (3.99-5.55)	2.35 (0.96-5.74)
1920	0.18 (0.13-0.24)	1.49 (1.39-1.6)	2.4 (2.12-2.72)	3.1 (2.77-3.48)	2.07 (1.08-3.97)
1930	0.28 (0.22-0.36)	1.67 (1.57-1.77)	2.26 (2.04-2.51)	2.17 (1.97-2.38)	1.54 (0.9-2.62)
1940	0.55 (0.46-0.65)	1.44 (1.37-1.53)	1.63 (1.49-1.79)	1.57 (1.45-1.71)	1.46 (0.91-2.34)
1950	Ref	Ref	Ref	Ref	Ref
1960	1.87 (1.55-2.24)	0.81 (0.75-0.89)	0.74 (0.65-0.85)	0.69 (0.62-0.78)	0.94 (0.49-1.8)
1970	3.12 (2.37-4.12)	0.73 (0.63-0.85)	0.71 (0.57-0.89)	0.57 (0.46-0.69)	1.43 (0.49-4.16)
1980	7.83 (4.96-12.35)	0.95 (0.74-1.21)	0.9 (0.61-1.31)	0.53 (0.37-0.77)	2.1 (0.39-11.22)
1990	15.92 (7.45-33.99)	1.2 (0.74-1.94)	1.37 (0.71-2.67)	0.91 (0.46-1.82)	–
P_{wald}	3×10^{-48}	1×10^{-71}	4×10^{-65}	7×10^{-114}	0.42
**Adenocarcinoma in a polyp**					
1900	–	0.25 (0.17-0.37)	0.66 (0.41-1.09)	1.15 (0.79-1.69)	–
1910	–	0.29 (0.25-0.33)	0.53 (0.46-0.62)	0.88 (0.77-1.01)	–
1920	–	0.4 (0.37-0.44)	0.58 (0.52-0.63)	0.89 (0.82-0.98)	–
1930	–	0.57 (0.53-0.62)	0.71 (0.66-0.77)	0.92 (0.86-0.99)	–
1940	–	0.79 (0.74-0.84)	0.84 (0.79-0.89)	0.95 (0.89-1.01)	–
1950	–	Ref	Ref	Ref	–
1960	–	1.2 (1.09-1.33)	1.29 (1.19-1.41)	1.1 (1.01-1.19)	–
1970	–	1.73 (1.43-2.1)	1.75 (1.49-2.06)	1.47 (1.26-1.71)	–
1980	–	2.78 (1.95-3.95)	3.2 (2.35-4.35)	1.9 (1.42-2.54)	–
1990	–	5.32 (2.4-11.81)	4.16 (2.12-8.19)	2.61 (1.36-5.03)	–
P_{wald}	–	5×10^{-118}	4×10^{-46}	3×10^{-6}	–
**Squamous cell carcinoma**					
1900	–	–	–	0.29 (0.09-0.97)	0.37 (0.18-0.76)
1910	–	–	–	0.17 (0.1-0.31)	0.34 (0.25-0.47)
1920	–	–	–	0.2 (0.14-0.3)	0.4 (0.33-0.49)
1930	–	–	–	0.3 (0.23-0.41)	0.43 (0.36-0.5)
1940	–	–	–	0.53 (0.42-0.67)	0.65 (0.57-0.74)
1950	–	–	–	Ref	Ref
1960	–	–	–	1.51 (1.18-1.95)	1.54 (1.35-1.77)
1970	–	–	–	1.79 (1.15-2.8)	1.85 (1.48-2.3)
1980	–	–	–	1.47 (0.42-5.17)	1.36 (0.84-2.21)
1990	–	–	–	–	1.76 (0.46-6.64)
P_{wald}	–	–	–	3×10^{-17}	3×10^{-33}
**Signet ring cell carcinoma**[a]					
1900	–	0.38 (0.14-1.01)		3.44 (0.83-14.24)	–
1910	0.08 (0.01-0.48)	0.65 (0.47-0.91)		0.86 (0.39-1.9)	–
1920	0.14 (0.04-0.48)	0.8 (0.63-1.02)		1.26 (0.78-2.02)	–

1930	0.16 (0.07-0.41)	1.1 (0.9-1.36)	1.19 (0.81-1.76)	–
1940	0.57 (0.3-1.09)	1.23 (1.02-1.47)	1.16 (0.84-1.6)	–
1950	Ref	Ref	Ref	–
1960	3.06 (1.53-6.1)	1.09 (0.85-1.39)	1.31 (0.89-1.92)	–
1970	6.27 (2.4-16.37)	1.56 (1.09-2.22)	1.43 (0.82-2.5)	–
1980	8.15 (1.7-39.06)	2.72 (1.58-4.7)	1.49 (0.69-3.26)	–
1990	–	3.34 (1.42-7.88)	1.26 (0.31-5.14)	–
P_{wald}	2×10^{-5}	5×10^{-8}	0.66	–
**Neuroendocrine neoplasm**[a]				
1900	–	0.24 (0.09-0.64)	0.21 (0.07-0.68)	–
1910	0.05 (0.01-0.18)	0.28 (0.2-0.4)	0.1 (0.06-0.17)	0.22 (0.04-1.13)
1920	0.17 (0.1-0.3)	0.29 (0.23-0.38)	0.15 (0.12-0.2)	0.25 (0.07-0.92)
1930	0.39 (0.27-0.56)	0.49 (0.41-0.6)	0.25 (0.21-0.29)	0.34 (0.11-1.04)
1940	0.55 (0.4-0.74)	0.68 (0.58-0.8)	0.5 (0.44-0.56)	0.52 (0.26-1.05)
1950	Ref	Ref	Ref	Ref
1960	1.95 (1.51-2.52)	1.32 (1.09-1.61)	1.72 (1.53-1.94)	2.06 (0.91-4.63)
1970	3.74 (2.79-5)	2.09 (1.51-2.89)	3.62 (3.05-4.3)	6.81 (2.06-22.52)
1980	6.72 (4.86-9.29)	4.44 (2.62-7.51)	8.07 (6.2-10.5)	7.68 (1.12-52.62)
1990	12.54 (8.63-18.24)	6.94 (2.39-20.19)	29.97 (17.86-50.31)	–
P_{wald}	2×10^{-49}	2×10^{-25}	1×10^{-127}	0.04

IRR, incidence rate ratio; CI, confidence interval; Ref, reference.

[a]Distal, proximal, and overlapping colon sites combined due to small numbers.

Table S17: Average annual percentage change of expected age-specific cancer incidence rates between 1990 and 2019, by sex and site after excluding neuroendocrine neoplasms.

Age, years	AAPC (95% CI)				
	Appendix	Proximal colon	Distal colon	Rectum	Anus
**Persons**					
20-29	4.97 (2.49, 7.52)	4.55 (2.79, 6.34)	4.51 (2.57, 6.49)	4.64 (3.68, 5.61)	0.91 (-3.13, 5.12)
30-39	6.99 (5.5, 8.51)	2.77 (1.9, 3.64)	3.47 (2.57, 4.38)	3.03 (2.62, 3.45)	-0.06 (-1.66, 1.57)
40-49	6.25 (5.23, 7.27)	-0.38 (-0.87, 0.11)	0.69 (0.22, 1.17)	0.55 (0.33, 0.77)	2.46 (1.64, 3.29)
50-59	5.64 (4.76, 6.54)	-1.6 (-1.89, -1.31)	-1.26 (-1.55, -0.96)	-1.15 (-1.29, -1.01)	3.28 (2.67, 3.89)
60-69	4.81 (3.98, 5.63)	-1.03 (-1.23, -0.83)	-1.73 (-1.95, -1.51)	-1.57 (-1.68, -1.47)	2.72 (2.18, 3.28)
70-79	4.91 (4, 5.84)	0.64 (0.47, 0.82)	-0.91 (-1.13, -0.69)	-1.23 (-1.34, -1.12)	0.95 (0.39, 1.51)
80-89	3.54 (2.12, 4.98)	1.34 (1.1, 1.58)	-0.49 (-0.82, -0.16)	-1.02 (-1.18, -0.85)	-0.04 (-0.86, 0.77)
90-99	1.42 (-2.6, 5.61)	1.03 (0.34, 1.73)	-1.44 (-2.39, -0.48)	-1.32 (-1.77, -0.86)	-0.87 (-2.72, 1.02)
Net drift[a]	5.09 (4.51, 5.67)	0.33 (0.12, 0.55)	-0.08 (-0.30, 0.15)	-0.19 (-0.29, -0.08)	1.57 (1.12, 2.03)
P_{wald}	0.02	5×10^{-79}	3×10^{-36}	3×10^{-131}	3×10^{-11}
**Males**					
20-29	3.67 (0.3, 7.17)	3.67 (1.23, 6.17)	5.32 (2.73, 7.98)	4.71 (3.01, 6.44)	-0.19 (-4.99, 4.85)
30-39	8.21 (6.05, 10.42)	2.33 (1.14, 3.54)	4.03 (2.78, 5.29)	3.55 (2.8, 4.3)	-1.3 (-3.21, 0.66)
40-49	7.24 (5.74, 8.77)	-0.11 (-0.78, 0.57)	0.8 (0.17, 1.43)	0.6 (0.21, 0.98)	1.76 (0.75, 2.78)
50-59	7.07 (5.7, 8.46)	-1.48 (-1.88, -1.08)	-1.15 (-1.51, -0.79)	-1.13 (-1.35, -0.91)	2.26 (1.52, 2.99)
60-69	5.34 (4.15, 6.55)	-1 (-1.27, -0.72)	-1.58 (-1.84, -1.33)	-1.56 (-1.73, -1.39)	1.41 (0.77, 2.05)
70-79	4.36 (2.99, 5.75)	0.37 (0.12, 0.63)	-0.92 (-1.18, -0.66)	-1.29 (-1.47, -1.11)	0.35 (-0.34, 1.06)

80-89	4.47 (2.01, 7)	0.99 (0.61, 1.38)	-0.72 (-1.15, -0.29)	-0.97 (-1.27, -0.68)	0.56 (-0.58, 1.72)
90-99	6.01 (-4.2, 17.31)	1.11 (-0.18, 2.42)	-1.48 (-2.98, 0.04)	-1.37 (-2.33, -0.4)	0.77 (-2.73, 4.4)
Net drift[a]	5.93 (4.91, 7.00)	0.24 (-0.06, 0.54)	0.06 (-0.24, 0.36)	-0.13 (-0.32, 0.06)	0.94 (0.35, 1.52)
P_{wald}	7×10^{-3}	3×10^{-25}	1×10^{-20}	3×10^{-46}	6×10^{-3}
**Females**					
20-29	6.31 (2.6, 10.15)	5.43 (3.78, 7.1)	3.78 (2.25, 5.34)	4.56 (3.08, 6.07)	1.57 (-5.62, 9.32)
30-39	5.78 (3.71, 7.9)	3.21 (2.4, 4.02)	3 (2.31, 3.7)	2.46 (1.81, 3.1)	1.1 (-1.8, 4.09)
40-49	5.3 (3.92, 6.7)	-0.67 (-1.14, -0.2)	0.6 (0.22, 0.98)	0.55 (0.19, 0.91)	2.92 (1.46, 4.39)
50-59	4.41 (3.24, 5.59)	-1.71 (-1.98, -1.44)	-1.34 (-1.58, -1.08)	-1.07 (-1.31, -0.83)	4.05 (2.96, 5.15)
60-69	4.31 (3.18, 5.46)	-1.06 (-1.25, -0.87)	-1.97 (-2.17, -1.77)	-1.63 (-1.83, -1.44)	3.86 (2.85, 4.88)
70-79	5.4 (4.16, 6.65)	0.86 (0.7, 1.02)	-1.21 (-1.4, -1.01)	-1.5 (-1.69, -1.3)	1.46 (0.49, 2.43)
80-89	2.91 (1.15, 4.7)	1.53 (1.33, 1.73)	-0.64 (-0.91, -0.37)	-1.48 (-1.73, -1.23)	-0.38 (-1.69, 0.96)
90-99	-0.1 (-4.52, 4.53)	0.93 (0.4, 1.47)	-1.6 (-2.28, -0.92)	-1.6 (-2.21, -0.98)	-1.26 (-3.98, 1.53)
Net drift[a]	4.50 (3.75, 5.26)	0.40 (0.21, 0.60)	-0.31 (-0.49, -0.14)	-0.36 (-0.52, -0.18)	2.15 (1.35, 2.96)
P_{wald}	0.08	4×10^{-123}	6×10^{-55}	2×10^{-46}	1×10^{-5}

AAPC, average annual percentage change; CI, confidence interval.

[a]AAPC of expected age-standardised incidence rates.

Table S18: Average annual percentage change of expected age-specific tumour incidence rates between 1990 and 2019, by sex and site for all histological subtypes combined.

Age, years	AAPC (95% CI)				
	Appendix	Proximal colon	Distal colon	Rectum	Anus
**Persons**					
20-29	6.59 (5.14, 8.06)	4.59 (2.95, 6.26)	4.59 (2.63, 6.58)	5.51 (4.62, 6.4)	0.96 (-3.13, 5.21)
30-39	6.46 (5.07, 7.86)	2.85 (2.04, 3.66)	3.55 (2.64, 4.46)	3.55 (3.16, 3.94)	0.12 (-1.48, 1.75)
40-49	6.48 (5.1, 7.88)	-0.31 (-0.77, 0.15)	0.75 (0.27, 1.23)	0.96 (0.74, 1.17)	2.56 (1.73, 3.39)
50-59	5.84 (4.43, 7.27)	-1.53 (-1.8, -1.26)	-1.23 (-1.52, -0.93)	-0.89 (-1.02, -0.75)	3.28 (2.67, 3.9)
60-69	4.72 (3.36, 6.09)	-0.97 (-1.16, -0.79)	-1.7 (-1.92, -1.48)	-1.39 (-1.49, -1.28)	2.73 (2.17, 3.28)
70-79	5.07 (3.47, 6.69)	0.67 (0.51, 0.84)	-0.9 (-1.12, -0.67)	-1.13 (-1.24, -1.02)	0.93 (0.37, 1.5)
80-89	4.85 (2.21, 7.57)	1.35 (1.13, 1.58)	-0.49 (-0.82, -0.15)	-0.98 (-1.14, -0.81)	-0.06 (-0.87, 0.76)
90-99	3.18 (-4.78, 11.81)	1.03 (0.37, 1.68)	-1.44 (-2.4, -0.47)	-1.32 (-1.77, -0.87)	-0.91 (-2.78, 1)
Net drift[a]	5.47 (4.56, 6.38)	0.39 (0.19, 0.58)	-0.04 (-0.27, 0.18)	0.08 (-0.02, 0.18)	1.61 (1.15, 2.07)
P_{wald}	0.62	7×10^{-88}	2×10^{-36}	9×10^{-178}	4×10^{-11}
**Males**					
20-29	6.58 (5.38, 7.8)	3.85 (1.51, 6.24)	5.46 (2.85, 8.12)	5.69 (4.23, 7.17)	-0.01 (-4.82, 5.04)
30-39	7.97 (6.83, 9.12)	2.41 (1.27, 3.57)	4.13 (2.87, 5.4)	4.07 (3.42, 4.72)	-0.95 (-2.84, 0.98)
40-49	7.75 (6.66, 8.85)	-0.07 (-0.71, 0.58)	0.85 (0.22, 1.49)	1.03 (0.7, 1.37)	1.77 (0.77, 2.78)
50-59	7.17 (6.08, 8.28)	-1.41 (-1.79, -1.02)	-1.12 (-1.48, -0.76)	-0.88 (-1.08, -0.68)	2.23 (1.5, 2.96)
60-69	5.04 (4.05, 6.05)	-0.94 (-1.21, -0.68)	-1.56 (-1.82, -1.3)	-1.41 (-1.56, -1.25)	1.39 (0.75, 2.03)
70-79	4.8 (3.59, 6.02)	0.41 (0.17, 0.66)	-0.91 (-1.18, -0.65)	-1.21 (-1.37, -1.04)	0.3 (-0.4, 1)
80-89	6.17 (3.81, 8.57)	1.02 (0.65, 1.39)	-0.72 (-1.15, -0.28)	-0.94 (-1.21, -0.67)	0.49 (-0.64, 1.64)
90-99	7.7 (-2.6, 19.08)	1.11 (-0.13, 2.38)	-1.47 (-2.98, 0.06)	-1.38 (-2.26, -0.49)	0.74 (-2.76, 4.37)
Net drift[a]	6.5 (5.61, 7.39)	0.3 (0.02, 0.58)	0.1 (-0.2, 0.4)	0.14 (-0.03, 0.31)	0.96 (0.38, 1.54)
P_{wald}	2×10^{-4}	4×10^{-27}	9×10^{-21}	1×10^{-75}	0.01
**Females**					

20-29	6.6 (4.58, 8.66)	5.31 (3.72, 6.93)	3.79 (2.26, 5.35)	5.32 (3.96, 6.7)	1.54 (-5.6, 9.22)
30-39	5.49 (3.56, 7.47)	3.27 (2.48, 4.06)	3.06 (2.37, 3.75)	2.96 (2.37, 3.56)	1.1 (-1.74, 4.03)
40-49	5.58 (3.6, 7.6)	-0.56 (-1.02, -0.1)	0.65 (0.28, 1.03)	0.91 (0.57, 1.25)	3.08 (1.64, 4.53)
50-59	4.79 (2.75, 6.87)	-1.64 (-1.9, -1.37)	-1.31 (-1.56, -1.06)	-0.79 (-1.02, -0.55)	4.08 (3, 5.17)
60-69	4.48 (2.42, 6.58)	-1.01 (-1.19, -0.82)	-1.95 (-2.15, -1.74)	-1.39 (-1.58, -1.2)	3.88 (2.88, 4.89)
70-79	5.34 (2.97, 7.77)	0.89 (0.73, 1.04)	-1.18 (-1.38, -0.98)	-1.38 (-1.56, -1.19)	1.47 (0.51, 2.44)
80-89	4.08 (0.49, 7.8)	1.53 (1.34, 1.73)	-0.63 (-0.9, -0.36)	-1.44 (-1.68, -1.19)	-0.36 (-1.67, 0.95)
90-99	1.79 (-7.9, 12.49)	0.93 (0.4, 1.46)	-1.6 (-2.28, -0.92)	-1.6 (-2.2, -0.99)	-1.3 (-4.01, 1.47)
Net drift[a]	4.87 (3.63, 6.13)	0.45 (0.26, 0.64)	-0.28 (-0.46, -0.11)	-0.08 (-0.24, 0.08)	2.19 (1.39, 2.99)
P_{wald}	0.89	2×10^{-124}	6×10^{-56}	1×10^{-64}	7×10^{-6}

AAPC, average annual percentage change; CI, confidence interval.
[a]AAPC of expected age-standardised incidence rates.

Table S19: Average annual percentage change of expected age-specific tumour incidence rates between 1990 and 2019, by histology and site.

**Age, years**	**AAPC (95% CI)**				
	**Appendix**	**Proximal colon**	**Distal colon**	**Rectum**	**Anus**
**Adenocarcinoma, NOS**					
20-29	3.02 (-0.8, 6.98)	4.69 (2.49, 6.94)	4.68 (2.49, 6.92)	5.4 (4.29, 6.53)	9.21 (0.48, 18.69)
30-39	6.52 (4.24, 8.85)	3.12 (2.05, 4.2)	3.67 (2.66, 4.68)	3.49 (3.02, 3.97)	-1.51 (-5.32, 2.45)
40-49	6.74 (5.15, 8.35)	-0.48 (-1.09, 0.12)	0.57 (0.05, 1.09)	0.55 (0.3, 0.8)	-2.22 (-4.53, 0.15)
50-59	4.51 (3.07, 5.96)	-1.77 (-2.13, -1.42)	-1.73 (-2.05, -1.41)	-1.34 (-1.49, -1.18)	-1.45 (-2.79, -0.09)
60-69	2.82 (1.57, 4.08)	-1.16 (-1.4, -0.92)	-2.21 (-2.45, -1.96)	-1.76 (-1.88, -1.64)	-2.02 (-3.09, -0.95)
70-79	3.59 (2.15, 5.06)	0.53 (0.31, 0.74)	-1.32 (-1.56, -1.08)	-1.33 (-1.46, -1.21)	-2.6 (-3.59, -1.6)
80-89	2.26 (0.14, 4.43)	1.24 (0.94, 1.53)	-0.87 (-1.24, -0.5)	-1.02 (-1.22, -0.83)	-2 (-3.28, -0.71)
90-99	2.1 (-5.24, 10.02)	0.93 (0.003, 1.87)	-2.04 (-3.15, -0.91)	-1.2 (-1.79, -0.6)	-2.34 (-4.98, 0.37)
Net drift[a]	4.16 (3.25, 5.08)	0.27 (0.01, 0.53)	-0.38 (-0.63, -0.13)	-0.17 (-0.3, 0.05)	-1.36 (-2.33, -0.38)
P_{wald}	5×10^{-3}	1×10^{-56}	7×10^{-38}	2×10^{-130}	0.13
**Mucinous adenocarcinoma**					
20-29	8.49 (4.76, 12.34)	2.52 (0.17, 4.94)	3.34 (0.08, 6.7)	2.41 (-0.97, 5.91)	–
30-39	7.43 (5.18, 9.74)	0.75 (-0.45, 1.97)	0.95 (-0.89, 2.83)	-1.32 (-3.07, 0.47)	4.08 (-3.89, 12.72)
40-49	5.86 (4.4, 7.33)	-1.56 (-2.27, -0.84)	-1.68 (-2.75, -0.6)	-2.8 (-3.77, -1.82)	1.81 (-3.49, 7.39)
50-59	6.32 (5.07, 7.58)	-2.83 (-3.27, -2.38)	-3.87 (-4.55, -3.19)	-4.03 (-4.63, -3.43)	-2.17 (-5.52, 1.3)
60-69	6.56 (5.31, 7.81)	-2.52 (-2.82, -2.22)	-4.01 (-4.5, -3.51)	-3.79 (-4.24, -3.34)	-2.13 (-4.71, 0.52)
70-79	5.81 (4.51, 7.14)	-0.16 (-0.42, 0.11)	-1.91 (-2.4, -1.42)	-3.34 (-3.79, -2.88)	-1.74 (-4.29, 0.88)
80-89	3.59 (1.48, 5.75)	0.88 (0.51, 1.26)	-1.38 (-2.1, -0.67)	-3.8 (-4.48, -3.12)	-2.1 (-5.69, 1.63)
90-99	3.46 (-3.92, 11.4)	1.37 (0.08, 2.68)	-2.3 (-4.57, 0.03)	-2.56 (-4.83, -0.24)	–
Net drift[a]	5.98 (5.06, 6.91)	-0.81 (-1.11, -0.51)	-1.95 (-2.41, -1.49)	-2.89 (-3.33, -2.45)	-0.78 (-2.78, 1.27)
P_{wald}	0.29	1×10^{-56}	2×10^{-14}	2×10^{-3}	0.74
**Adenocarcinoma in a polyp**					
20-29	–	5.77 (1.73, 9.98)	4.43 (1.03, 7.94)	2.93 (-0.32, 6.29)	–
30-39	–	4.28 (2.51, 6.07)	4.63 (3.08, 6.2)	2.78 (1.33, 4.24)	–
40-49	–	2.78 (1.8, 3.78)	2.84 (2.01, 3.68)	1.93 (1.15, 2.71)	–
50-59	–	2.14 (1.56, 2.71)	2.21 (1.71, 2.7)	0.72 (0.25, 1.2)	–
60-69	–	2.85 (2.46, 3.26)	1.71 (1.34, 2.09)	0.42 (0.06, 0.79)	–
70-79	–	3.43 (3.07, 3.79)	1.89 (1.49, 2.3)	0.31 (-0.06, 0.69)	–
80-89	–	3.44 (2.87, 4.01)	1.45 (0.76, 2.15)	0.2 (-0.41, 0.82)	–

90-99	–	2.41 (0.46, 4.4)	-0.72 (-3.12, 1.73)	-1.27 (-3.09, 0.59)	–
Net drift[a]	–	3.17 (2.7, 3.63)	2.29 (1.87, 2.70)	0.97 (0.58, 1.35)	–
P_{wald}	–	0.02	3×10^{-3}	3×10^{-3}	–
**Squamous cell carcinoma**					
20-29	–	–	–	–	-0.25 (-6.58, 6.52)
30-39	–	–	–	-0.14 (-6.12, 6.22)	-0.63 (-2.93, 1.72)
40-49	–	–	–	2.96 (0.68, 5.29)	3.11 (1.98, 4.26)
50-59	–	–	–	5.37 (3.76, 7)	4.43 (3.55, 5.33)
60-69	–	–	–	6.14 (4.59, 7.71)	4.35 (3.5, 5.21)
70-79	–	–	–	4.95 (3.15, 6.79)	2.4 (1.52, 3.3)
80-89	–	–	–	2.8 (0.22, 5.44)	1.12 (-0.29, 2.55)
90-99	–	–	–	-1.82 (-7.33, 4.01)	0.46 (-2.99, 4.04)
Net drift[a]	–	–	–	3.75 (2.59, 4.92)	2.46 (1.75, 3.18)
P_{wald}	–	–	–	0.04	2×10^{-5}
**Signet ring cell carcinoma[b]**					
20-29	–	3.89 (-0.21, 8.15)		-0.63 (-6.92, 6.08)	–
30-39	5.02 (-2.12, 12.69)	4.69 (2.03, 7.42)		0.67 (-2.79, 4.26)	–
40-49	9.61 (4.47, 15)	2.25 (0.45, 4.08)		1.8 (-0.99, 4.68)	–
50-59	8.78 (4.37, 13.36)	-0.59 (-1.9, 0.74)		0.61 (-1.61, 2.89)	–
60-69	9.48 (4.61, 14.56)	-0.49 (-1.52, 0.54)		-0.87 (-2.78, 1.07)	–
70-79	7.37 (1.57, 13.5)	2.13 (1.2, 3.08)		-0.41 (-2.34, 1.55)	–
80-89	3.44 (-4.27, 11.78)	2.69 (1.31, 4.08)		1.65 (-1.92, 5.34)	–
90-99	–	3.83 (-0.99, 8.9)		-4.92 (-11.14, 1.73)	–
Net drift[a]	7.92 (5, 10.93)	1.74 (0.96, 2.52)		0.04 (-1.22, 1.31)	–
P_{wald}	0.65	1×10^{-4}		0.56	–
**Neuroendocrine neoplasm[b]**					
20-29	6.24 (4.99, 7.51)	6.19 (0.88, 11.79)		11.15 (8.49, 13.88)	–
30-39	6.38 (5.08, 7.69)	6.23 (3.62, 8.9)		8.02 (6.76, 9.3)	6.81 (-2.02, 16.43)
40-49	6.81 (5.26, 8.38)	3.75 (2.08, 5.44)		6.64 (5.73, 7.56)	10.07 (3.68, 16.85)
50-59	6.58 (4.69, 8.5)	3.4 (2.2, 4.62)		6.39 (5.54, 7.25)	7.09 (1.88, 12.57)
60-69	4.88 (2.96, 6.85)	3.59 (2.6, 4.59)		7.28 (6.39, 8.19)	5.48 (-0.22, 11.5)
70-79	5.91 (3.27, 8.61)	4.29 (3.23, 5.35)		6.04 (4.87, 7.22)	3.83 (-1.69, 9.65)
80-89	10.67 (4.01, 17.76)	2.85 (1.32, 4.41)		4.35 (1.96, 6.79)	2.25 (-4.13, 9.05)
90-99	–	1.01 (-3.72, 5.97)		-1.59 (-7.04, 4.17)	–
Net drift[a]	6.47 (5.41, 7.55)	3.89 (3.07, 4.71)		6.25 (5.57, 6.94)	6.08 (2.55, 9.72)
P_{wald}	0.60	0.24		3×10^{-4}	0.72

AAPC, average annual percentage change; CI, confidence interval.

[a]AAPC of expected age-standardised incidence rates.

[b]Distal, proximal, and overlapping colon sites combined due to small numbers.
